# Non-coding RNAs profiling in head and neck cancers

**DOI:** 10.1038/npjgenmed.2015.4

**Published:** 2016-01-13

**Authors:** Daria Salyakina, Nicholas F Tsinoremas

**Affiliations:** 1 Center for Computational Science, University of Miami, Coral Gables, FL, USA; 2 Department of Medicine, Miller School of Medicine, University of Miami, Coral Gables, FL, USA

## Abstract

The majority of studies on human cancers published to date focus on coding genes. More recently, however, non-coding RNAs (ncRNAs) are gaining growing recognition as important regulatory components. Here we characterise the ncRNA landscape in 442 head and neck squamous cell carcinomas (HNSCs) from the cancer genome atlas (TCGA). HNSCs represent an intriguing case to study the potential role of ncRNA as a function of viral presence, especially as HPV is potentially oncogenic. Thus, we identify HPV16-positive (HPV16^+^) and HPV-negative (HPV^−^) tumours and study the expression of ncRNAs on both groups. Overall, the ncRNAs comprise 36% of all differentially expressed genes, with antisense RNAs being the most represented ncRNA type (12.6%). Protein-coding genes appear to be more frequently downregulated in tumours compared with controls, whereas ncRNAs show significant upregulation in tumours, especially in HPV16^+^ tumours. Overall, expression of pseudogenes, antisense and short RNAs is elevated in HPV16^+^ tumours, while the remaining long non-coding RNA types are more active in all HNSC tumours independent of HPV status. In addition, we identify putative regulatory targets of differentially expressed ncRNAs. Among these ‘targets’ we find several well-established oncogenes, tumour suppressors, cytokines, growth factors and cell differentiation genes, which indicates the potential involvement of ncRNA in the control of these key regulators as a direct consequence of HPV oncogenic activity. In conclusion, our findings establish the ncRNAs as crucial transcriptional components in HNSCs. Our results display the great potential for the study of ncRNAs and the role they have in human cancers.

## Introduction

The expression of protein-coding genes (messenger RNAs (mRNAs)) has been the focus of pathophysiological studies for decades. However, in recent years this concept has been challenged by the discovery of non-coding RNAs (ncRNAs) and their interactions with proteins or other mRNAs. More specifically, according to Ensemble^1^ (v76), only 34% of human transcriptomes are protein-coding genes. The remaining 66% are non-coding, with the largest group represented by pseudogenes (24%), followed by long intergenic non-coding RNAs (lincRNAs; 13%), antisense RNAs (asRNAs; 9%), and micro RNAs (miRNAs; 6.6%). However, it is not clear in what proportion coding and non-coding genes are expressed in various tissues and under various disease conditions, or whether they have any role. Thus, understanding the function of ncRNAs provides an opportunity to formulate new paradigms involved in biological systems and to devise novel therapies and diagnostic tools.

There have been a number of reports describing how the transcription of ncRNAs can affect almost all stages of the gene expression process, although the specific molecular mechanisms by which ncRNAs contribute to gene expression regulation are complex and not fully understood. One of the proposed mechanisms of long ncRNAs (lncRNAs) and pseudogene-mediated regulation is the competition for shared miRNA between protein-coding mRNA and ncRNA.^2^ Such ncRNAs are also known as competing endogenous RNAs (ceRNAs).^2^ In addition, linc- and asRNA can also mediate gene regulation by guiding chromatin modifiers to specific genomic loci or modulating translational control.^3,4^ asRNA can be involved in transcriptional interference by (1) engaging in promoter competition with the genes in the *cis* position; (2) blocking elongation in *trans*; (3) masking specific splice sites; or (4) mediating exon skipping.^5^ Furthermore, asRNA can have a positive or negative impact on post-translational control of a sense mRNA.^6^ Scarce examples of the regulatory importance of ncRNA emphasise the emerging need for studying its role more systematically.

In this report, we profile both ncRNAs and mRNAs in head and neck squamous cell carcinomas (HNSCs). This type of cancer is comprised of malignancies arising from the mucosal lining of the oral cavity, oropharynx, hypopharynx, larynx, sinonasal tract and nasopharynx, and is frequently associated with such risk factors as human papilloma virus (HPV) infection, tobacco use and poor oral hygiene.^7,8^ HPV-associated HNSC more often arises from the tonsil and base of tongue, and are less likely to have an associated history of tobacco or alcohol use.^9^ Multiple oncogenic HPV types (16, 18, 31, 33, 35, 39, 51 and 66) are associated with HNSC.^10,11^ Overall, HPV16 and 33 are the most frequently detected types.^10,11^ These viruses express oncogenes E6, E7 and E5, which promote cancer cell proliferation, migration and invasion.^12,13^ The HPV-associated HNSC prevalence may vary by geographic region from 20 to 90% and has increased over the past decades.^14,15^ These changes can be attributed to differences in sexual culture (i.e., more oral sex partners or oral sex at an earlier age in recent generations) combined with a decrease in tobacco use.

HPV-positive (HPV^+^) and HPV-negative (HPV^−^) HNSCs are considered to be two distinct cancer forms. Both mutational landscape^16^ and gene expression^11^ profiles differ significantly between HPV^+^ and HPV^−^ HNSCs. Previous studies using large-scale sequencing technologies have clearly demonstrated that HPV^+^ versus HPV^−^ HNSCs express different sets of transcription factors and cell cycle regulators, and that the differential expression of host mRNAs is a direct consequence of HPV oncogenic activity.^5,11,17,18^ Furthermore, certain canonical cancer genes (*TP53, CDKN2A, PIC3CA, CASP8, NOTCH1, RB1, TP63* and *FAT1*) are though to be impaired in both subtypes through mutations in HPV^−^ cases, or through direct or indirect interaction with HPV oncogenes in virus-positive cases.^16,19^

The majority of the studies on HNSCs published to date focus on coding genes. At the same time, the non-coding portion of the transcriptome has gained growing recognition as an important regulatory component in various biological processes.^3^ Several studies have already demonstrated compelling evidence of the utility of miRNAs as biomarkers for HNSC,^20,21^ but only a few focus on lncRNAs. Among the few examples, expression of such non-coding genes as *HOTAIR, CDKN2B-AS (ANRIL), MALAT1, MEG3, NEAT1* and *UCA1* has been shown to be associated with HNSC.^22,23^ Three recent transcriptome studies demonstrated pervasive deregulation of ncRNAs in HNSC.^24–26^ Shen *et al.* ^25^ reported two uncharacterised transcripts, *AC026166.2* and *RP11-169D4.1*, to be differentially expressed in laryngeal squamous cell carcinoma and associated with poorer prognosis for survival. In another study by Zou *et al.*,^24^ suppressed expression of two antisense transcripts, *RP1-91G5.3* and *RP11-475O23.3*, was correlated with poorer survival.

To our knowledge, no comprehensive reports studying the expression of ncRNAs and their potential regulatory targets in HNSC have been published. Also, there are no reports on effects of HPV on the ‘dark matter’ of the transcriptome. To bridge this gap, we evaluated profiles of ncRNAs from both HPV^+^ and HPV^−^ HNSC specimens in the RNA-seq data from nearly 500 HNSC samples from the cancer genome atlas (TCGA).^27^ This data provides a unique opportunity to study the potential role of ncRNA as a function of viral presence, especially as HPV is potentially oncogenic.^28^ In this study, we report that 35–40% of differentially expressed genes in HNSC are coming from the ncRNAs. In addition, we find that HPV^+^ tumours have a unique non-coding expression landscape compared to HPV^−^ tumours. Furthermore, we identify the putative targets of various ncRNA types enriched in HPV^+^ and HPV^−^ tumours and evaluate their function and role in oncogenesis. The regulatory potential of ncRNAs makes these molecules promising biomarkers for prediction, diagnosis, prognosis and management of HNSC.

## Results

### HPV detection in HNSC TCGA cohort

Utilising the pipeline outlined in the materials and methods, we detected four HPV types in 33.5% of the HNSC tumours and 14.0% in the control samples. The most common type was HPV16 (25.2%), followed by HPV18 (3.4%) and HPV33 (3.8%). HPV35 was the least common type in this cohort (1%; Supplementary Tables 1 and 2).

The presence of HPV in HNSC samples varied from a single read to up to 548,539 reads per transcriptome (Supplementary Table 2). We identified two clearly distinct groups of HNSC samples, namely low (<200 reads) and high (>2000 reads) viral expression (Supplementary Figure 3, Supplementary Table 2). Typically, only one HPV type per sample was detected with HPV16 being the dominant type in the ‘high-expression’ group. HPV18 was detected at low levels only (3.4%) while HPV33 at both low (2.2%) and high (1.6%) expression levels. Because of the small sample size, we have not considered tumours with high HPV33 expression as a separate comparison group for differential gene expression (DE) testing. Further, we compared tumours with high expressions of HPV16, 33 and 35 to determine whether they can be combined. As a result, we found that 218 genes were differentially expressed between types 16 and 35; and 21 genes were differentially expressed between types 16 and 33. In order to reduce heterogeneity, we decided to focus primarily on the HPV16^+^ group and discard high HPV33 and 35 tumours in this study.

Further, we evaluated whether high HPV16 expression is associated with certain anatomic sites and whether it would confound our ncRNA interrogation. As presented in Supplementary Table 3, HPV16^+^ tumours were more often located in the base of tongue or tonsil, whereas HPV^−/low^ and control samples were more often located in the floor of mouth, larynx, oral cavity and other locations. In HPV16^+^ tumours, no single gene was significantly differentially expressed between combined HNSC from the tonsil (TON, *N*=28) and HNSC located in the base of tongue (BOT, *N*=13) versus remaining sites (OTHER, *N*=13) enriched in HPV^−^ group and controls (Supplementary Table 3).

In HPV^−^ tumours, 9 out of 19,716 tested genes showed significant differential expression between TON (*N*=5)+BOT (*N*=8) and OTHER (*N*=276) tumour sites (Supplementary Table 4). Five out of these nine genes were not associated with HPV16 status in our study and thus were not relevant. From the remaining genes, three showed independent effect of HPV16 status on gene expression according to our follow-up ANOVA, showing no confounding effect of the tumour site over all. The expression of only one gene (HMGN2P18) was confounded by tumour anatomical location. In summary, we have shown that the tumour site does not introduce systematic bias in our findings (1 gene only) and does not need to be corrected for.

To elucidate whether the differences in HPV expression could be explained owing to lymphocyte, monocyte and neutrophil infiltration or the percentage of tumour cells, tumour nuclei, stromal cells, normal cells or necrosis, we evaluated the association of these variables with HPV levels. None of the above was associated with HPV levels (Supplementary Table 5).

The presence of two clearly distinct groups of tumours, namely low and high viral expression tumours (Supplementary Figure 3, Supplementary Table 3), raised the question of whether low HPV expression has the same impact on the tumour transcriptome as high HPV expression, or whether it is similar to the virus-free group instead. To answer this question, we compared gene expression profiles in the following four groups: (1) HPV16^+^ tumours with high viral expression; (2) HPV^low^ expressing tumours; (3) HPV^−^ tumours; and (4) controls (adjacent normal tissue). Supplementary Material contains detailed explanations of sample grouping.

### Gene expression analysis in HPV16^+^, HPV^low^ and HPV^−^ HNSC

As described in Supplementary Materials and Methods, we used a pipeline based on the Cufflinks workflow to detect expressions of transcripts from 537 TCGA samples for HSCN. For DE testing, we used 17,757 annotated genes expressed with FPKM>1 in at least 50% of samples in at least one of the four groups.

**Figure 1:**
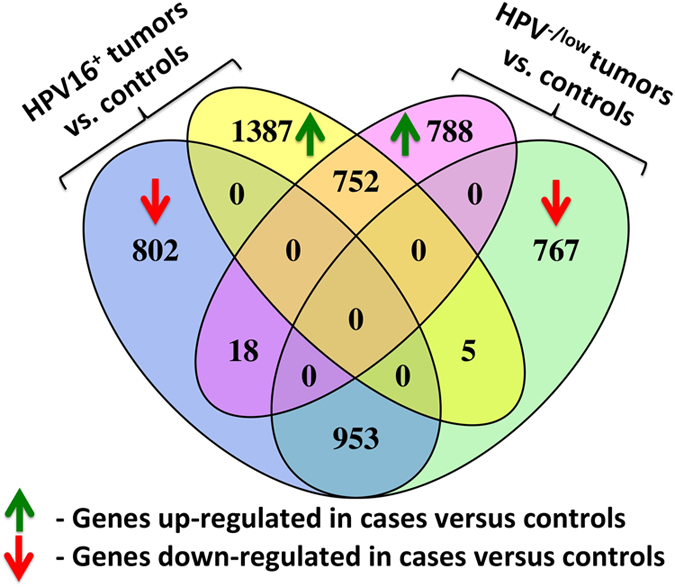
Venn diagram of differentially expressed genes in tumours versus control samples. Blue and yellow ovals show the numbers of down- and upregulated genes in 54 HPV16^+^ tumours versus 24 controls, correspondingly. Pink and green ovals show the numbers of down- and upregulated genes in 341 HPV^−/low^ tumours versus 24 controls. Controls include three low HPV33 expressing samples and one low HPV16 expressing sample (<200 reads). The majority of differentially expressed genes between cases and controls were specific to the HPV16^+^ or HPV^−/low^ tumours. Only about a third of the genes were significantly up- or downregulated in both HPV16^+^ and HPV^−/low^ tumours compared with controls.

**Figure 2:**
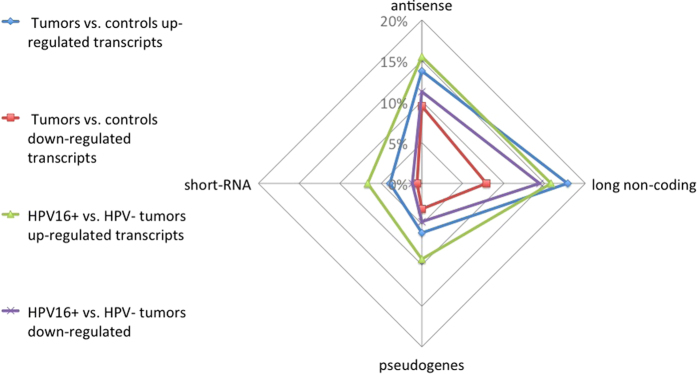
Proportions of differentially expressed ncRNA by biotype in tumours versus normal and HPV16^+^ versus HPV^−^ comparisons. Here we separated differentially expressed (DE) genes into two categories: (1) commonly up- and downregulated genes between cases and controls (shown in intersections on Figure 1) independent of HPV status, denoted ‘tumours vs controls’; (2) remaining DE genes specific to HPV16 status, called ‘HPV16^+^ vs HPV^−^ ’ DE genes (see Materials and Methods). In this diagram, 100% corresponds to the total number of DE annotated genes per comparison, including coding genes. Only non-coding gene proportions are shown here. Pseudogenes, antisense RNA, and short RNA appear to be more up-regulated in HPV16^+^ tumours compared with HPV^−^ tumours. The remaining long non-coding RNA types are more upregulated in tumours compared with controls, independent of HPV status. Overall, ncRNA is more often upregulated in tumours.

The overall differences in gene expression profiles for HPV16^+^ tumours versus HPV^−^ tumours were at least as significant as the differences between tumours compared with control samples (Table 1). In contrast, gene expression profiles between HPV^low^ and HPV^−^ tumours did not show any significant differences (Table 1). The similarity in gene expression between HPV^low^ and HPV^−^ tumours suggests the functional insignificance of HPV at such low levels in a tumour’s makeup, although it does not exclude past involvement of the virus in tumorigenesis. As there was no notable difference between these two groups, and five control samples also had a low expression level of HPV, we combined HPV^low^ and HPV^−^ tumours into one group when comparing with controls, namely HPVl6^−/low^. As Figure 1 shows, roughly half of the differentially expressed genes were attributed to differences between tumour and control tissue, independent from HPV status. The remaining half of the differentially expressed genes were regulated based on HPV16 presence or absence (Figure 1). HPV16^+^ tumours had more significant expression changes than HPVl6^−/low^ tumours, compared with the controls (|fold change|>2 and *P*<0.05); in particular more genes were upregulated (Figure 2). The differential expression of a few genes (*n*=23) showed changes in the opposite direction in tumour versus control comparisons, depending on the HPV16 expression level (Figure 1). These genes could be of interest, as they could serve as biomarkers for virus-associated HNSC. Supplementary Table 6 shows that 15 out of these 23 genes were previously reported to be associated with various cancers or alternated response to the chemotherapy.

### Identification of differentially expressed ncRNA biotypes

In this report, we evaluate the composition of DE non-coding genes in HNSC. Ensembl^1^ annotation (v76) distinguishes >20 different types of transcripts (biotypes) with 31% being protein coding and the rest non-coding RNAs.^1^ Overall, 71% of isoforms and 60% of genes detected in this study were novel and not annotated by Ensembl (Supplementary Figure 2). Among the 17,757 annotated genes expressed in HNSC samples and used for DE testing, protein-coding genes constituted the majority (68%), followed by asRNAs (11%), lincRNAs (9%) and pseudogenes (5%; Table 2). The median expression level (FPKM) of ncRNAs was ~4.5 times lower than that of protein-coding RNAs (Supplementary Figure 4).

**Figure 3:**
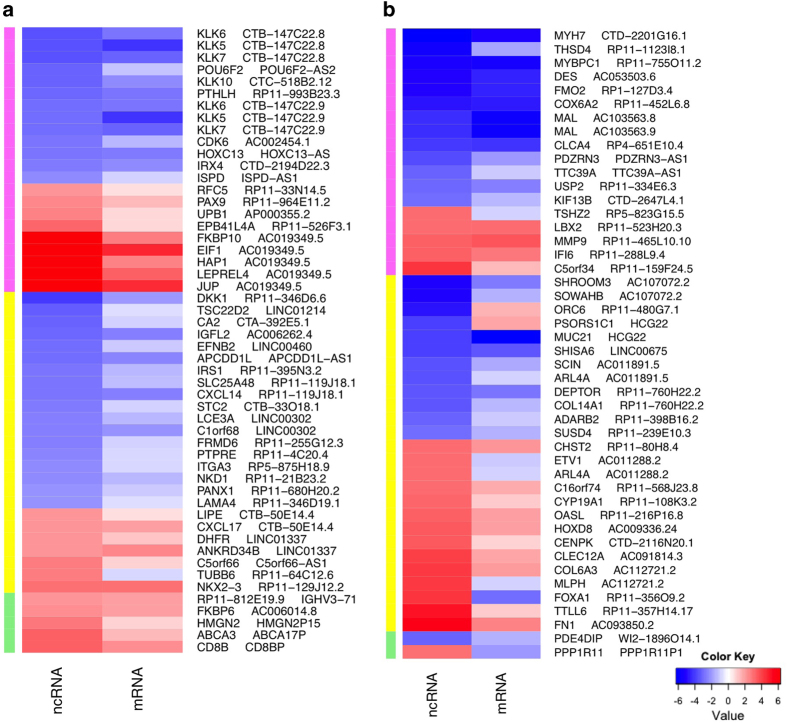
Gene expression changes (log2) heat maps for the top ninety-eight differentially expressed ncRNA and their potential regulatory targets. (**a**) HPV16^+^ versus HPV^−^ comparison; (**b**) Tumours versus controls. All ncRNAs shown in this figure have >10-fold expression changes. All shown ‘target’ mRNAs are at least twofold differentially expressed. Colours on the left side of each heat map depict different groups of ncRNAs: pink—asRNA, yellow—other lncRNA, green—pseudogenes.

The proportion of differentially regulated genes by biotype was unique to tumour/control and HPV16^+^/HPV^−^ comparisons (Figure 3). Protein-coding genes were more frequently downregulated in tumours compared with controls, whereas ncRNAs were more frequently upregulated in tumours and even substantially more in HPV16^+^ tumours (Figure 2, Supplementary Table 7). The proportions of significantly upregulated ncRNAs in HPV16^+^ tumours were 1.5 to 5 times higher contrasted to tumour/control test (Figure 2).

### Identification of putative targets for ncRNAs

After potential targets for significantly DE ncRNAs were identified as described in Materials and Methods, we have catalogued 1398 ncRNA-‘target’ pairs (Supplementary Table 8). The expression of 30% of the identified potential target genes varied more than twofold. The most prominent differences in expression (over 10-fold) were observed in 98 pairs of ncRNAs and their DE potential targets (Figure 3). In 91% of these pairs, positive expression correlation of ncRNAs and their targets was observed.

Finally, the majority of detected putative target genes were associated with only one type of ncRNA (Supplementary Figure 9), suggesting very little or no redundancy in this type of expression co-regulation. These findings suggest the important role of ncRNAs in HNSC, particularly in virus-positive cases.

### Long non-coding RNA (asRNA, lincRNA, processed transcript, sense intronic and sense overlapping)

The most represented biotype among expressed non-coding genes in our study was lncRNA (including asRNA), accounting for 26.7% of the significant difference in gene expression (Supplementary Table 7). asRNA genes were the largest DE group of long non-coding biotypes, comprising 12.7% of all DE genes (Supplementary Table 7 and Table 2). The expression of 179 asRNAs varied uniquely in tumours compared to controls, whereas 353 asRNAs were HPV16^+^ tumour specific (Supplementary Table 7). On the basis of the known mechanisms of asRNAs controlling the expression of adjacent genes, we identified all potential regulatory targets for DE asRNAs as described in the Materials and Methods section. Expression analysis revealed a strong trend towards positive correlation between asRNAs and their putative regulatory targets (Supplementary Figure 5). Out of 751 potential asRNA target genes co-located in the same locus, 548 (73%) were protein coding and 40% of them were DE (Supplementary Tables 8 and 9). The proportion of differentially expressed protein coding targets was twofold higher (40%) than the proportion of DE protein coding genes overall (22%).

**Figure 4:**
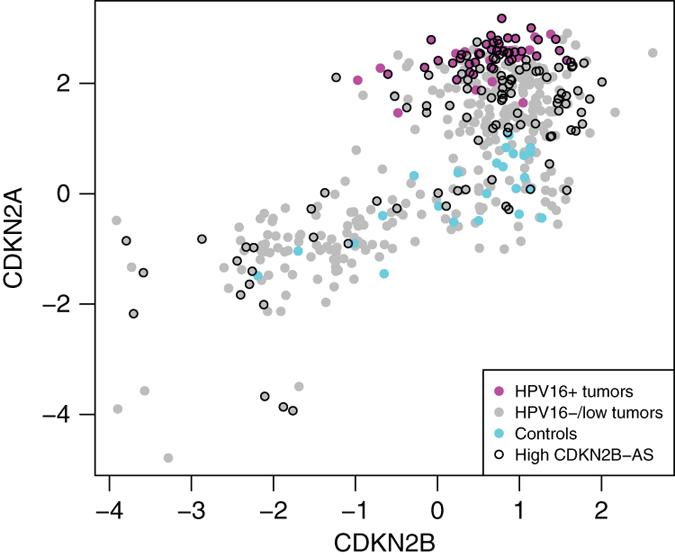
RNA expression levels (z-scores) of *CDKN2A* and *CDKN2B* protein coding genes stratified by their antisense transcript, *CDKN2B-AS,* expression. All HPV16^+^ tumours show elevated *CDKN2A* (also known as *p16* or *INK4A*) and *CDKN2B* expression. Controls, in contrast, have low *CDKN2A* and variable *CDKN2B* expression. There is visible positive correlation between *CDKN2A, CDKN2B* and *CDKN2B-AS*.

One of the most upregulated protein-coding genes in HPV^+^ cancers was *CDKN2A*. Its antisense transcript, *CDKN2B-AS*, was 44-fold induced in HPV16^+^ tumours and 15-fold induced in HPV^−/low^ tumours compared with control tissue. Expression of all three genes (*CDKN2A, CDKN2B* and *CDKN2B-AS*) was positively correlated in HNSC samples (Figure 4). Interestingly, expression of *CDKN2B-AS* was elevated to a greater extent in the HNSC tumours compared with controls rather than in HPV16^+^ tumours. In addition, another lncRNA located within *CDKN2B-AS* intron, *RP11-149I2.4,* also showed more prominent expression in HPV16^+^ tumours and might be a novel regulatory lncRNA for HPV-associated *CDKN2A* over-expression (Figure 4).

**Figure 5:**
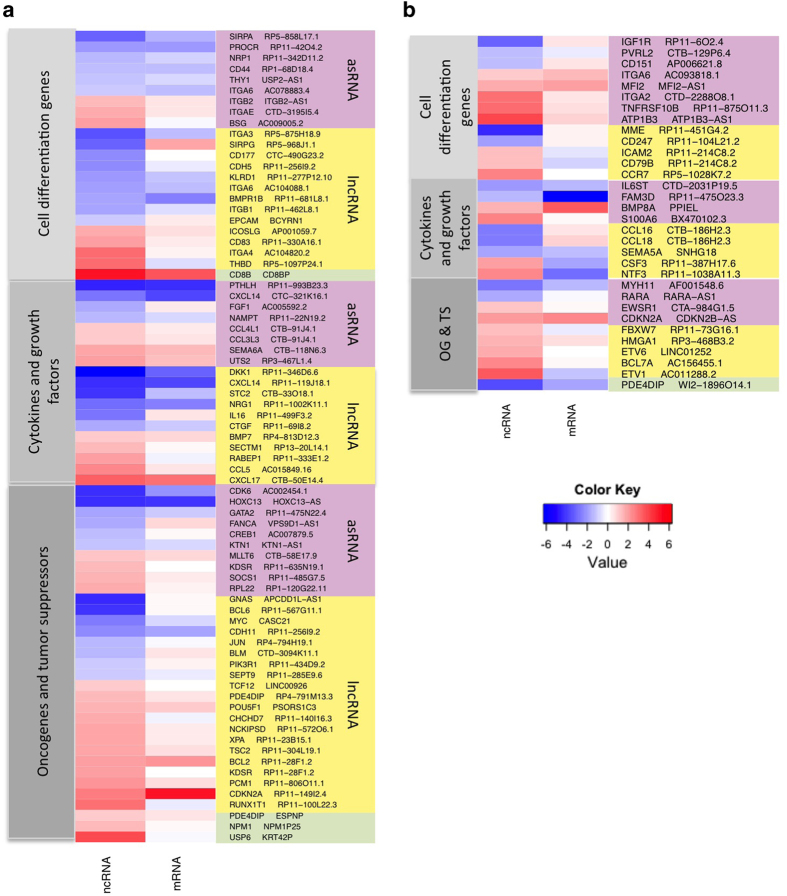
Gene expression change (log2) for pairs of non-coding RNA (ncRNA) and corresponding potential target mRNA. Genes are organised by gene family and ncRNA type. Pink-shaded pairs correspond to asRNA and their potential targets, yellow-shaded to remaining lncRNA and their potential targets and green-shaded to the pseudogenes and their potential targets. (**a**) HPV16^+^ versus HPV^−^ comparison; (**b**) Tumours versus controls.

In addition to *CDKN2A*, a number of well-established tumour suppressors and oncogenes were identified as potentially regulated by asRNA. A full list of the most interesting targets is presented in the Table 3. Corresponding gene expression changes can be found in the Figure 5 and Supplementary Table 8. Interestingly, transcription factors and RNA-binding proteins^29^ were most frequently found to be potential targets for ncRNAs (Table 3), suggesting the significant role of ncRNA in the regulation of transcription and translation. Network analyses of putative asRNA targets using GeneGo Metacore has shown significant over-representation (*P*-value=8.03e-285) of genes regulated by the CREB1 transcription factor in HPV16^+^ tumours. CREB1 RNA was not DE between virus-positive and virus-negative tumours, however, its asRNA, *AC007879.5*, was significantly downregulated in HPV16^+^ tumours. CDK6, HOXC13 and MYH11 were among the downregulated transcripts co-expressed with and potentially regulated by their asRNAs (Figure 5).

The remaining four types of lncRNAs (‘lincRNA’, ‘processed transcript’, ‘sense intronic’ and ‘sense overlapping’) were accountable for 14.04% of the DE genes. Expression levels of these 397 lncRNA genes varied significantly between HPV16^+^ and HPV^−^ tumours, and 193 lncRNA genes were consistently DE between tumours and controls independent from the presence of HPV (Table 2, Supplementary Table 7). Potential targets for these 590 DE lncRNAs were assigned by physical proximity on the chromosome, based on current scientific understanding of how lincRNAs affect the expression of neighboring genes. However, this approach is a simplistic way to capture potential lncRNA targets.

Similarly to asRNA-‘target’ pairs, expression levels in the majority of lncRNA-‘target’ pairs were positively correlated, although we do find some examples of negative correlation (Supplementary Figure 6). The strength of the correlation decreased over genomic distances. At short distances (25–1,000 nucleotides), average correlation in pairs was 0.34. Dependency in expression levels in ‘head-to-tail‘ oriented lncRNA-‘target’ pairs was the greatest at short distances (average *r*=0.54 between 25 and 100 nucleotides; and *r*=0.44 between 25 and1,000 nucleotides) compared with alternately oriented gene pairs.

### Pseudogenes

The third largest group of differentially expressed ncRNAs was comprised of various pseudogene biotypes (Table 2). Out of 872 expressed pseudogenes, 257 (29%) were significantly differentially regulated (|fold change|>2, *P*<0.05). Seventy pseudogenes were attributable to the tumour versus control differences, and 187 pseudogenes to the presence of HPV16. Using BLAST, we were able to identify 119 protein-coding parent genes for the 257 differentially expressed pseudogenes (Supplementary Figure 8). Nearly 22% (*N*=26) of the pseudogenes’ putative targets (in this case their parent genes) were RNA-binding proteins (Table 3). Unlike lncRNAs and asRNAs, the expression of pseudogenes did not show a tendency towards positive correlation with the expression of their putative targets (Supplementary Figure 7). Two groups of parent genes had more than one significantly DE pseudogene for tumours/control (*N*=5) and HPV16^+^/HPV^−^ (*N*=9) comparisons, respectively: *DDX11, FAM98B, RPS27, BMS11, C2CD3* and *GUSB, HMGN2, IFITM3, IFNLR1, PPIA, RNF181*, *RP11-812E19.9, UBA52* and *UNG* (Supplementary Table 10).

Network analyses using MetaCore (https://portal.genego.com/) of parent genes from the DE pseudogenes in HPV16^+^ and HPV^−^ tumours showed significant overrepresentation of networks associated with the cell cycle, the fibroblast growth factor pathway, Notch receptor signaling, and the viral life cycle (Supplementary Table 11). In addition, GO processes involving viral infection were significantly overrepresented among parent genes associated with pseudogenes DE in HPV16^+^ tumours (Supplementary Table 12 and Table 4). Expression changes in 15 out of 18 parent genes involved in viral infection were in the same direction as the expression of corresponding pseudogenes in HPV16^+^ tumours (Table 4). Interestingly, in tumour/control comparison this concordance in expression of pseudogene–parent gene pairs either reversed direction (*N*=7) or disappeared (*N*=6). Our findings suggest that pseudogenes of parent genes involved in viral infection may have a significant role in regulating these processes themselves.

## Discussion

HPV^+^ and HPV^−^ HNSC are considered to be two distinct cancer forms. HPV^+^ HNSC are more frequently associated with the tonsil or base of tongue, and are less likely to have an associated history of tobacco or alcohol use.^9^ In this paper, we demonstrate the great magnitude of changes in the non-coding fraction of the HPV16^+^ and HPV^−^ HNSC transcriptome. The expression profile of coding genes was previously reported by Tang *et al.* ^11^ in a subset (*N*=304) of the HNSC cohort discussed here (*N*=442). In our study, we observed very similar changes in the expression landscape of protein-coding genes to those described earlier (data not shown). Here we do not focus on the protein-coding genes; instead, we use them as a reference point for studying the ncRNAs expression.

Our results show that only 40% of transcripts detectable by NGS are ‘known’ genes annotated by Ensembl (v76).^1^ Among annotated DE genes, about a third were non-coding. These findings are consistent with the recently reported study that showed ~25% of lncRNAs being DE between HNSC and controls.^24^ This fact alone suggests that the ncRNAs changes in expression are a widespread global phenomenon and should have a critical role in HNSC. We also demonstrate here that the median expression of ncRNAs was ~4.5 times lower than of protein coding genes, consistent with the findings described by Cabili *et al*.^30^ A new study published during our manuscript review by Yan *et al.* 2015^26^ demonstrated that ncRNA had higher cancer subtype specificity than protein coding genes. Yan with colleagues, however, did not stratify HNSC by HPV status, neither attempted to identify putative regulatory targets for DE nsRNA.

Strikingly, our data show more frequent downregulation of protein-coding genes in tumours compared with controls and more frequent upregulation of ncRNAs in tumours, especially in HPV16^+^, compared with controls. Pseudogenes, asRNAs and short RNAs are more upregulated in HPV16^+^ tumours compared with HPV^−^ tumours, whereas remaining long non-coding RNA types are more upregulated in tumours compared with controls independent from HPV status. These findings infer the importance of non-coding transcripts in rapidly dividing cells. Viruses, in turn, might contribute to the increased activation or deregulation of non-coding machinery in the tumour. On the other hand, the elevated number of differentially expressed ncRNAs may result from the recruitment of additional tumour-infiltrating cells with differing ncRNA makeup. Tumours are complex entities comprised of cancer cells, immune cells, fibroblasts, stromal cells, extracellular matrix, blood vessels and so on. Cancer cells may constitute as little as 30% of a tumour.^31^ In fact, TCGA selection criteria declares 60% of tumour nuclei in the samples to be sufficient. This means that potentially up to 40% of TCGA samples are composed of the various cell types. For instance, elevated expression of *CD8B* gene and its pseudogene *CD8BP* in HPV16^+^ HNSC tumours compared with HPV^−^ tumours can indicate increased lymphocyte infiltration of the HPV16^+^ tumour. The CD8 antigen is a cell-surface glycoprotein found on most cytotoxic T lymphocytes that mediates the killing of cancer cells.^32^ At the same time, the presence of the CD8^+^ lymphocytes has a positive effect on survival.^31^ This coincides with the fact that patients with HPV^+^ profiles have a six times higher probability to respond to conventional (chemo-) radiotherapy compared with those with HPV^−^ HNSC profiles.^33^

lncRNA operates on many levels; in particular it has an important role in regulation of genome organisation, methylation and gene expression.^4^ Increasing understanding of ncRNA functions has uncovered its essential role in cell differentiation and development. In this study, in addition to profiling the ncRNA expression landscape, we attempted to identify the potential regulatory protein-coding targets of ncRNAs. The underlying mechanisms of ncRNA-mediated regulation are far from being understood, especially for *trans*-acting ncRNAs. At the same time, multiple examples exist of *ci*s-regulation through potential mRNA expression level control of genes located in the vicinity of lncRNA transcription sites.^3,4,6^ In this manuscript, we focus on the putative *cis*-acting lncRNAs. In the case of pseudogenes, we restricted our search to the corresponding parent genes. We understand that our approach will discover only a fraction of potential regulatory targets. Nevertheless, it sets solid ground for the future functional studies. Once knowledge about the exact mechanisms by which ncRNA contributes to cellular processes advances, this approach can be refined.

Multiple mechanisms of lncRNA regulation describing both positive and negative regulation by lncRNAs have been reviewed recently.^3,4,6^ However, it is still unclear which mechanisms are more prevalent in cancer. In our study, we have shown that the majority of lncRNAs (including asRNAs) show an overall trend towards positive correlation in expression with their potential targets in the same loci. This co-expression could result from either the active enhancement of neighbouring gene expression by lncRNA or the involvement of co-regulatory mechanisms of adjacent genes mediated by a third party, for both mRNA and ncRNA. Also, in the case of asRNA partially overlapping with protein-coding genes, accurate expression estimates from unstranded libraries could be misinterpreted and lead to artificial positive correlations. To disambiguate our findings and better understand the regulatory mechanisms involved, future studies are necessary.

In this paper, we identified several well-established oncogenes, tumour suppressors, cytokines, growth factors and cell differentiation genes that may serve as potential targets of differentially expressed ncRNAs. Transcription factors and RNA-binding proteins were most represented among the ncRNA ‘targets’. Interestingly, lncRNAs and asRNAs were primarily co-expressed with the transcription factors, whereas pseudogenes most frequently had RNA-binding proteins^29^ as a parent gene. RNA-binding proteins^29^ are known to regulate various processes such as gene transcription, RNA processing, splicing and degradation. They are especially important because of their potential role in downstream ncRNA regulation.

The most prominent differentially expressed ncRNAs between HPV16^+^ and HPV^−^ were associated with protein coding ‘targets’ involved in the cell cycle, cell–cell signaling and epidermis, ectoderm and endothelium development. For instance, kallikrein genes *KLK5, KLK6* and *KLK7* were downregulated in HPV16^+^ tumours and co-expressed with two overlapping antisense transcripts: *CTB-147C22.8* and *CTB147C22-9*, as well as *KLK10* with its antisense *CTC-518B2.12*. Kallikreins are a subgroup of serine proteases having diverse physiological functions. Apart from the epithelium differentiation, growing evidence suggests that many kallikreins are implicated in carcinogenesis and some have potential as novel cancer biomarkers. *KLK5*, for instance, was reported to be downregulated in breast cancer,^34^ whereas elevated expression of *KLK6, KLK7* and *KLK10* has been associated with multiple cancers and poor prognosis in gastric and colorectal cancer.^35–39^

Among all non-coding biotypes, asRNA was the largest group with significant differences in expression. The most prominently induced asRNA in HPV16^+^ tumours was *AC019349.5,* with a fold change over 752. This asRNA is located in the locus containing genes *JUP, FKBP10, HAP1, LEPREL4* and *EIF1* upregulated in HPV16^+^ tumours. *EIF1* is a eukaryotic translation initiation factor 1, required for maintaining the fidelity of canonical transcription initiation sites at the AUG codon.^40^ *JUP* is a tumour suppressor gene and its expression is associated with oesophageal squamous cell carcinoma aggresiveness.^41,42^ Downregulation of *FKBP10* was previously linked to unfavourable outcomes in ovarian carcinoma patients.^43^ *HAP1* gene expression is associated with radiosensitivity in breast cancer.^44^ And finally, *LEPREL4* is a promising tumour-associated autoantigen for prostate cancer.^45^ All putative targets of *AC019349.5* described above have been previously associated with some aspects of carcinogenesis and are potentially interesting biomarkers for HPV16^+^ tumours. However, before any conclusions about the *AC019349.5* ability to regulate expression of neighbouring genes can be made, the ‘guilt by association’ needs to be confirmed in additional experiments. Interestingly, based on the literature from previous studies referenced above, the expression changes of genes potentially regulated by *AC019349.5* and kallikreins in the HPV16^+^ HNSC tumours align with evidence of HPV^+^ HNSC’s better response to conventional terapy.^33^

Following asRNAs, pseudogenes comprised the second largest group of DE ncRNAs in our study. Pseudogenes are known to regulate the mRNA level of their parent genes trough competition for common miRNAs.^2^ In our study, we found a number of DE pseudogenes with corresponding parent genes significantly overrepresented in processes consistent with HNSC and HPV16 infection. The most significant pathways included the cell cycle, fibroblast growth factor, Notch signaling and viral life cycle pathways. These findings indicate the potential involvement of pseudogenes in control of ribosomal activation and in the increase of protein synthesis during HPV16-alternated cell cycle.

Previous studies have identified several ncRNAs showing differential regulation in various HNSC anatomical sites. The most studied ncRNAs in HNSC include *ANRIL*, *HOTAIR, SOX2OT, NEAT-1, MALAT-1* and *UCA-1* associated with nasopharyngeal, pharyngeal, laryngeal, esophageal, oral or tongue squamous cell carcinomas or metastatic tumours.^22,46–48^ We were not able to detect expressions of *HOTAIR, SOX2OT* and *MEG-3.* Previous reports on these three genes were produced using low throughput PCR-based methods involving cDNA amplification and it is possible that the expression of these transcripts is too low to be detected in the TCGA NGS data.

Transcripts for *UCA-1, NEAT-1* and *MALAT-1* were detected in TCGA cohort, but did not show differences larger than twofold. Similarly, two non-coding genes previously associated with laryngeal squamous cell carcinoma and poor prognosis for survival,^25^ *AC026166.2* and *RP11-169D4.1*, did not appear DE in our study. One possible explanation for the lack of association of these genes with *HPV16*^*+*^ *or HNSC* in our study could be the heterogeneous nature of HNSC. Most previous studies were done on a small number of samples from a single anatomic site or on metastatic tumours. In contrast, the findings reported here were not associated with any particular anatomical site and originate from primary tumours only in the largest cohort of HNSC tumours studied so far. For instance, *UCA-1, HOTAIR* and *NEAT-1* were previously shown to be primarily associated with metastasised HNSC^47^ while *MALAT-1* was upregulated in laryngeal HNSC.^22^ At the same time, multiple research groups have failed to show associations of these genes with other anatomical locations.^22,23^ In addition, most previous studies do not report HPV status and there is no consensus so far on the significance of these ncRNAs for HNSC.

From all previously reported genes above, only *ANRIL* was 4.6-fold upregulated in HNSC tumours. This gene, also known as *CDKN2B-AS,* was previously reported to mediate specific repression of the tumour suppressors locus *CDKN2A*–*CDKN2B*.^49^ However, the HNSC tumours studied here seem to express all three transcripts at substantially higher levels, particularly in HPV16^+^ tumours. Elevated expression of *CDKN2A* (also known as tumour suppressor *INK4A* or *p16*) is often used as a surrogate marker for HPV infection.^50^ Not surprisingly, expression of *CDKN2A* was 114 times higher in HPV16^+^ HNSC tumours compared with the controls and 34 times higher compared with HPV^−^ tumours. Recent studies demonstrated that *CDKN2B-AS* inhibits cell cycle checkpoints and promotes cell cycle progression. This asRNA is upregulated by *E2F1* in an *ATM*-dependent manner after DNA damage, and epigenetically represses the expression of *CDKN2A–CDKN2B* locus due to methylation of histone H3 at lysine 27 (H3K27me) at the late-stage of DNA damage response, which allows the cell to return to normal at the completion of the DNA repair.^49,51^ However, this mechanism described in a cell line does not explain observed positive correlations between these three genes in HNSC. Apparently, in HPV16^+^ HNSC tumours the *ATM–E2F1* signalling pathway is altered so that elevated expression of *CDKN2B-AS* no longer controls transcriptional silencing of *CDKN2A*–*CDKN2B* locus, but in contrary is associated with elevated expression of these tumour-suppressor genes. According to the orientation of *CDKN2B-AS* and *CDKN2A* on the chromosome, this sense–antisense pair can be classified as ‘head-to-head’ oriented. Two *CDKN2A* isoforms have 5′-UTR overlapping the 5′ end of *CDKN2B-AS* gene sequence, whereas other isoforms have alternative promoters at various distances from the *CDKN2B-AS*. As a result, different isoforms can potentially be regulated by alternative mechanisms at the post-transcriptional level due to complementarity to *CDKN2B-AS* RNA, or by *CDKN2B-AS*’s act of transcription at the transcription initiation level.

In addition, another lncRNA located in the *CDKN2A* intron*, RP11-149I2.4*, was also 6.6 times (*P*<0.05) upregulated in HPV16^+^ tumours compared with HPV^−^ tumours. The function of this poorly characterised ncRNA is not known and we suggest that it may induce HPV16-dependent *CDKN2A* expression. In this study we refrain from speculations regarding the exact mechanisms by which these two asRNA may control expression of *CDKN2A*. However, we are convinced that the scientific community will follow-up on these findings and shed more light on their molecular function in cancer.

A recent lncRNA profiling study by Zou *et al.* ^24^ on 31 HNSC tumour–normal pairs has identified 2,808 significantly differentially expressed lncRNAs and 33 small nucleolar RNAs. Two asRNAs, *RP1-91G5.3* (lnc-LCE5A-1) and *RP11-475O23.3* (lnc-KCTD6-3), were consistently downregulated in HNSC tumours compared with controls in Zou *et al.*^24^ cohort. We were not able to detect the expression of *RP1-91G5.3* in TCGA cohort, but have observed 2.8-fold downregulation of *RP11-475O23.3* in HNSC tumours versus control tissue. Two potential targets of this asRNA, *FAM3D* and *FAM107A*, were also downregulated in TCGA tumours (77.1 and 25.8-fold, respectively). *FAM107A* has been previously associated with various cancers, including neuroblastoma,^52^ renal cell carcinoma^53,54^ and fibrosarcoma.^55^ At the same time, the expression of *FAM3D* in patients’ blood was proposed as an early biomarker for colon cancer.^56^ Our findings extend the growing body of evidence on the roles of ncRNAs in HNSCs. To our knowledge, this is the first large-scale study profiling ncRNAs in HPV16^+^ HNSC and comparing it with HPV^−^ tumours.

Despite the overwhelming evidence of HPV’s association with HNSC,^8,10,11^ no critical threshold of HPV expression in the tumour affecting host-gene expression was established. In this study we clearly show that only higher expression levels of HPV (>2,000 detected reads) impact both coding and non-coding host-gene expression. The differences in HPV expression could not be explained by lymphocyte, monocyte and neutrophil infiltration, or by the percentage of tumour cells, tumour nuclei, stromal cells, normal cells or necrosis. The causes for low HPV expression remain unclear and need further investigation. Lower levels might reflect either sample contamination as outlined in the paper by Cantalupo *et al.*^57^ or the infection of a small fraction of the tumour cells. However, the lack of impact of HPV^low^ on the tumour transcriptome does not exclude its involvement in oncogenesis at early pre-cancerous stages.

In conclusion, we demonstrated that HPV16 presence contributes markedly to the ncRNA expression profile of the HNSC. We also established ncRNAs as important transcriptional RNA populations in HPV16-associated HNSCs and move forward with completing HNSC transcriptome profiling through detailed cataloguing of significant non-coding genes and their potential regulatory targets. Although we do not offer complete annotation of all possible regulatory targets of ncRNAs, we provide a solid foundation for future efforts to elucidate the roles of these transcripts. Our follow-up pathway analysis of potential targets is designed to aid in prioritisation of hypothetical targets. This kind of precise information is very valuable in the age of big data, when mountains of results can overwhelm scientists. Detailed experimental validation of the effects of hundreds of ncRNA on their targets will require tremendous resources and the involvement of multiple research groups over many years.

We strongly believe that studying ncRNA in virus-positive and virus-negative HNSCs provides opportunities to formulate new paradigms that govern biological systems, and to devise novel therapies and diagnostic tools. The tissue-specific nature of ncRNAs makes them promising biomarkers.^30,58^ Owing to novel discoveries of ncRNA role in cancer, the well-known hallmarks that have previously governed the study of tumorigenesis must be redefined to take into account aspects of ncRNA regulation. According to the GENCODE consortium,^58^ a majority of ncRNAs are yet to be discovered and may outnumber protein-coding genes.

## Materials and Methods

RNA-seq data in BAM format was downloaded for 537 HNSC samples from TCGA CGHub repository (https://tcga-data.nci.nih.gov/tcga/; October 2014). HNSC cohort included 495 HNSC patients. Informed consent was obtained from all subjects by TCGA centers (http://cancergenome.nih.gov/abouttcga/policies/informedconsent). Normal adjacent tissue from 42 patients was available as control. Anatomical sites for the tumours and control samples are presented in Supplementary Table 3. To detect HPV sequences in the RNA-seq data we employed the computational subtraction procedure, previously described by Salyakina and Tsinoremas.^59^ Reference sequences were downloaded from Ensemble^1^ for human (v76) and from PaVe^60^ (Papilloma Virus Database, pave.niaid.nih.gov) for 161 annotated HPV genomes in October 2014. No screening for other human viruses was done in this study. Tophat2^61^/Cufflinks^62^ pipeline was utilised for human transcript assembly and quantification (for details see Supplementary Methods, Supplementary Figure 1).

After data quality assessment, 71 samples (13%) had either low mapping rate (<85%) or elevated number of identified distinct isoforms (outside of one s.d.) as shown in Supplementary Figure 2. We were not able to identify source of extreme variation in these samples and suspect some underlying batch effects that were not traceable because of incomplete information on sequencing technologies and data QC parameters used by TCGA consortium. These samples were removed from the differential expression analysis (Supplementary Table 1).

Standardised z-transformed log_2_ gene-level FPKM values were calculated in the remaining 442 tumour samples and 24 controls, and used in differential gene expression (DE) testing. DE tests were considered significant if differences in expression between the groups were greater than twofold and *t*-test *P*-values were <0.05. *P*-values were corrected for multiple testing using FDR procedure with following Bonferrony correction for four contrasts: (1) HPV16^+^ *versus* HPV^−^ tumours, (2) HPV16^+^ *versus* controls, (3) HPVl^−/low^ tumours *versus* controls, and (4) HPV^low^ *versus* HPV^−^ tumours. For clarity, we separated DE genes into two categories: (1) all commonly DE genes between cases and controls independent from the HPV status we call ‘tumours vs normal’ DE genes; (2) remaining DE genes, specific to the HPV status are called ‘HPV^+^ versus HPV^−^’ DE genes.

In order to interrogate systematic bias from differences in anatomical location we have compared gene expression in tumours from base of tongue and tonsil with tumours from other sites within HPV16^+^ and HPV^−^ groups separately using *t*-test. Computational procedure and correction for multiple testing was identical to original tests. Genes that showed significant difference in expression between tumour sites were subjected to follow-up analysis of variance (ANOVA) analysis, testing independent effects of tumour site and HPV status.

Expressed transcripts were annotated with biotype information provided by Ensemble.^1^ Various types of pseudogenes (processed, unprocessed, transcribed, unitary and polymorphic) were consolidated into one group called ‘pseudogenes’. Technically, asRNA, lincRNA, sense intronic, sense overlapping and processed transcripts belong to the lncRNA category. Here we discuss asRNA genes as a separate group, owing to their more specific properties, and consolidate the remaining lncRNA genes into an unspecific lncRNA group. We assigned potential target genes for lncRNA and asRNA based on physical proximity on the chromosome. For asRNA, we included all overlapping genes from corresponding loci, independent from orientation, into the potential targets list. For lncRNA, the two closest neighbouring genes were selected as potential targets and stratified into four groups based on the ‘head’/’tail’ orientation of lncRNA gene relative to the ‘target’ gene. ‘Parent genes’ for all annotated pseudogenes were identified based on sequence similarity. Only one best mRNA hit found by BLAST^63^ was assigned as a ‘parent gene’ for each pseudogene transcript. Because of the lack of knowledge about possible targeting mechanisms, no attempt to identify targets for shortRNA was made.

Pearson correlation was calculated for ncRNA types and corresponding potential regulatory target genes. Genego MetaCore (https://portal.genego.com/), GSEA (http://www.broadinstitute.org/gsea/) and DAVID bioinformatics resources^64,65^ were used for ncRNA regulatory potential evaluation. RNA-binding proteins census^29^ was used to determine the putative targets of DE ncRNAs.

## Figures and Tables

**Table 1:** Number of differentially expressed (DE) genes in four comparisons

*Comparison (number of samples in the group)*	*Number of DE genes*
High HPV16^+^ (54) versus HPV^−^ tumours (286)	3214
High HPV16^+^ (54) versus controls (24)	3917
HPV^low^ (52) versus HPV^−^ (286)	0
HPV^−/low^ (341) versus controls (24)	3283

**Table 2:** Number of genes expressed in HNSC cohort

*Transcript type*	*Ensembl* *Biotype*	*Number of expressed genes used in DE analysis*	*DE genes HPV16*^*+*^ *tumour vs. HPV*^−^ *tumour*	*DE genes HPV16*^*+*^ *tumour vs. controls*	*DE genes HPV*^−*/low*^ *tumour vs. controls*
Coding	Protein-coding	12,147	1,779	2,367	2,067
					
Long non-coding	antisense	1,976	380	440	350
(asRNA and lncRNA)	lincRNA	1,568	362	359	364
	Sense intronic	248	38	50	26
	Processed transcript	159	24	33	29
	Sense overlapping	52	7	8	6
					
Pseudogenes	Polymorphic pseudogene	4	1	2	1
	Processed pseudogene	457	100	94	77
	Transcribed processed pseudogene	73	15	21	15
	Transcribed unprocessed pseudogene	177	39	38	30
	Unitary pseudogene	23	8	9	7
	Unprocessed pseudogene	138	40	35	24
					
Short non-coding	miRNA	286	43	39	11
	misc RNA	295	39	44	18
	snoRNA	82	13	20	20
	snRNA	72	18	11	9
					
Total number of genes with assigned biotype	17,757	2,906	3,570	3,054	
No biotype assigned by Ensembl	1,959	308	347	229	

**Table 3:** Most important genes potentially regulated by non-coding RNA

*Comparison*	*Gene category*	*Gene family*	*List of genes*
HPV16^+^ versus HPV^−^	lncRNA ‘targets’	Oncogenes (*N*=17)	*BCL2, BCL6, CDH11, CHCHD7, GNAS, JUN, KDSR, MYC, NCKIPSD, PCM1, PDE4DIP, POU5F1, RABEP1, RUNX1T1, SEPT9, TCF12* *, TCF3*
		Tumour suppressors (*N*=5)	*BLM, CDKN2A, PIK3R1, TSC2, XPA*
		Protein kinases (*N*=22)	*ACVR2A, BMP2K, BMPR1B, CDC42BPB, CDK1, DYRK1A, DYRK1B, EPHB1, HSPB8, MAP2K6, MAP3K9, MAPKAPK5, PDK2, PIK3R4, PXK, RYK, SGK1, STK3, TNK2, ULK1, CDS1, PIK3C3*
		Cell differentiation markers (*N*=13)	*BMPR1B, CD177, CD83, CDH5, EPCAM, ICOSLG, ITGA3, ITGA4, ITGA6, ITGB1, KLRD1, SIRPG, THBD*
		TFs (*N*=59)	*ACVR2A, AHR, ARID3B, BCL6, BRD7, CIR1, CITED2, DLX1, DMRTA2, DPF1, EN1, FAM20C, FOXC1, FOXE1, FOXF2, FOXM1, GRHL1, GTPBP1, IKZF2, IKZF3, IRF2, IRX2, JUN, KEAP1, KLF10, KLF11, KLF13, KLF7, MEF2A, MEOX1, MYC, NFIA, NKX2-3, POU5F1, PPARGC1A, PRDM11, PRDM2, PRDM8, RCOR1, RNF13, RNF144A, RUNX1T1, SOX21, SOX4, SOX5, SREBF2, ST18, TCF12, TCF3, TEAD4, TSC22D2, ZBTB25, ZEB2, ZFY, ZNF180, ZNF235, ZNF423, ZNF461, ZNF706*
		RNA-binding proteins (*N*=35)	*TNPO1, NOL10, RDM1, CAPRIN2, PARN, XRN2, SMNDC1, PUS1, RNASE10, RPP14, FAM46A, CRYZ, CNOT7, LSM14A, LSM14B, EXOSC5, AGO1, AGO3, PPARGC1A, CWC27, LUC7L, TYW5, RAE1, HNRNPD, SMN2, DHX8, RPS4Y1, KRR1, EIF4B, EFTUD1, FRG1B, FRG1, ANGEL1, GTPBP1, MAGOHB*
		Cytokines and growth factors (*N*=11)	*BMP7, CCL5, CTGF, CXCL14, CXCL17, DKK1, IL16, NRG1, RABEP1, SECTM1, STC2*
			
	asRNA ‘targets’	Oncogenes (*N*=8)	*CDK6, CREB1, GATA2, HOXC13, KDSR, KTN1, MLLT6, RPL22*
		Tumour suppressors (*N*=2)	*FANCA, SOCS1*
		Protein kinases (*N*=8)	*CAMK4, CDK6, GSG2, MAP4K1, MAPK6, MAST4, PDK1, ZAK*
		Cell differentiation markers (*N*=9)	*BSG, CD44, ITGA6, ITGAE, ITGB2, NRP1, PROCR, SIRPA, THY1*
		TFs (*N*=32)	*BRPF3, CORO1A, CREB1, CSRP1, CTCF, FOXA1, FOXD3, GATA2, GTF3C2, GTPBP1, HDAC2, HOXA5, HOXA7, HOXC13, IRX4, KLF11, LSR, MLLT6, NAB1, NRIP1, PAX9, PER3, POU6F2, PRDM6, SATB2, SIM2, SOX15, SPEN, TBX2, ZFY, ZNF131, ZNF337*
		RNA-binding proteins (*N*=24)	*DHX29, PSPC1, TEFM, SLTM, DDX49, EIF1, INTS8, RBM19, ZCCHC24, MOV10, FXR2, CTU2, EIF2B4, DDX28, MRPL14, CPEB2, SPEN, NOB1, TRNT1, RPL22, GTPBP1, IFIT1, IFIT2, IFIT3, YARS2*
		Cytokines and growth factors (*N*=8)	*CCL3L3, CCL4L1, CXCL14, FGF1, NAMPT, PTHLH, SEMA6A, UTS2*
			
	pseudogene ‘targets’	Oncogenes (*N*=3)	*NPM1, PDE4DIP, USP6*
		Protein kinases (*N*=2)	*CAMKK1*
		TFs (*N*=4)	*GTF2A2, HMGN2, TAF5L, TBC1D10B*
		RNA-binding proteins (*N*=16)	*RPL37, PSPC1, SNRPD2, RPLP1, GAPDH, RPS24, NUDT16, UBA52, MPHOSPH10, NPM1, POLR2K, MRPL39, FCF1, EIF4H, RPL23, MAGOH*
		Cell differentiation markers (*N*=1)	*CD8B*
			
Tumours versus controls	lncRNA ‘targets’	Oncogenes (*N*=5)	*BCL7A, CD79B, ETV1, ETV6, HMGA1*
		Tumour suppressors (*N*=1)	*FBXW7*
		Protein kinases (*N*=5)	*ERN1, GSK3B, MYLK3, RPS6KA5, STK35*
		Cell differentiation markers (*N*=5)	*CCR7, CD247, CD79B, ICAM2, MME*
		TFs (*N*=24)	*BANP, BRF1, CREG1, ETV1, ETV6, FOXA1, FOXD1, HES1, HMGA1, HNF4G, HOXD8, MIS18BP1, MLXIP, MSX2, NFE2L3, NR4A1, PKNOX1, SMARCAL1, SP3, TCF4, ZFPM1, ZHX2, ZNF157, ZNF304*
		RNA-binding proteins (*N*=8)	*RPL14, OASL, ADARB2, PET112, CNOT2, AAR2, ERN1, OBFC1, WDR61*
		Cytokines and growth factors (*N*=5)	*CCL16, CCL18, CSF3, NTF3, SEMA5A*
			
	asRNA ‘targets’	Oncogenes (*N*=4)	*EWSR1, IL6ST, MYH11, RARA*
		Tumour suppressors	*CDKN2A*
		Protein kinases (*N*=8)	*EPHA2, GUCY2C, IGF1R, LRRK1, MAPK6, PDK1, PDPK1, SNRK*
		Cell differentiation markers (*N*=9)	*ATP1B3, CD151, IGF1R, IL6ST, ITGA2, ITGA6, MFI2, PVRL2, TNFRSF10B*
		TFs (*N*=12)	*CASZ1, EGR3, GTPBP1, HOXB7, LBX2, MYBBP1A, RARA, SP2, TBC1D10B, TSHZ2, ZBTB47, ZNF35*
		RNA-binding proteins (*N*=15)	*FARS2, POLR2B, RBMS3, CWF19L1, NOA1, TARBP2, U2AF2, POLR2L, WARS, LENG9, PTRHD1, HNRNPA1, EWSR1, THOC6, GTPBP1*
		Cytokines and growth factors (*N*=4)	*BMP8A, FAM3D, IL6ST, S100A6*
			
	pseudogene ‘targets’	Oncogenes (*N*=1)	*PDE4DIP*
		Protein kinases (*N*=2)	*PAK4, SMG1*
		RNA-binding proteins (*N*=10)	*RPL37, RPS27A, RPS27, SMAD2, BMS1, SMG1, HNRNPA1, FAM98B, RPL29, RPL35A*
		TFs (*N*=3)	*BPTF, SMAD2, ZNF461*

**Table 4:** Differentially expressed pseudogenes and their parent genes involved in processes associated with viral infection

*Pseudogene name*	*Parent gene name*	*Log2-fold change*			
		*Pseudogene, HPV16*^*+*^ *versus HPV*^−^	*Parent gene, HPV16*^*+*^ *versus HPV*^*−*^	*Pseudogene, tumours versus controls*	*Parent gene, tumours versus controls*
*AC010677.5*	*RPL23*	−4.87	−0.15	5.49	−0.46
*TCEB2P2*	*TCEB2*	−3.53	−0.11	3.08	−0.2
*RPL37P2*	*RPL37*	−2.61	−0.07	−0.93	−0.07
*PPIAP26*	*PPIA*	−2.32	−0.03	3.97	0.61
*WTAPP1*	*MMP1*	−2.13	−4.33	3.08	5.19
*UNGP3*	*UNG*	−1.73	0.84	1.42	−0.03
*UBA52P8*	*UBA52*	−1.43	0.18	1.34	−0.18
*RP11-490K7.4*	*GTF2A2*	1.69	0.04	1.58	−0.28
*UBA52P6*	*UBA52*	3.16	0.18	1.34	−0.18
*EIF4HP2*	*EIF4H*	1.16	−0.11	1.32	−0.15
*AC114737.3*	*FDPS*	1.87	0.42	0.02	0.06
*RP1-89D4.1*	*RPS24*	2.17	0.36	−0.36	−0.92
*POLR2KP1*	*POLR2K*	1.82	0.11	−0.51	0.27
*CD8BP*	*CD8B*	5.12	3.55	−0.58	−0.56
*RP11-54C4.1*	*RPLP1*	1.32	0.28	−0.7	−0.47
*UNGP1*	*UNG*	1.13	0.84	−0.81	−0.03
*YWHAEP7*	*YWHAE*	2.25	0.03	−1.35	−0.08
*NPM1P25*	*NPM1*	1.46	0.25	−1.84	0.02
