# Supplementary material for: Non-coding RNAs profiling in head and neck cancers
Source: NPJ Genom Med. 2016 Jan 13;1:15004–. doi: 10.1038/npjgenmed.2015.4 (PMC5685291; doi:10.1038/npjgenmed.2015.4)
Supplement: Supplemental Table 1 [file npjgenmed20154-s1.pdf]

Supplemental table 1.: Proportion of samples with HPV

HPV type	Cases (high HPV expression)	Controls (high HPV expression)
HPV16	125 (58)	3 (1)
HPV18	17 (0)	0
HPV33	19 (8)	3 (0)
HPV35	5 (3)	0
