# Supplementary material for: Non-coding RNAs profiling in head and neck cancers
Source: NPJ Genom Med. 2016 Jan 13;1:15004–. doi: 10.1038/npjgenmed.2015.4 (PMC5685291; doi:10.1038/npjgenmed.2015.4)
Supplement: Supplemental Table 2 [file npjgenmed20154-s2.pdf]

Supplemental table 2: Sample information and HPV detection in HNSC							
ID	HPV type	Viral nucleotides count	Total Number of Reads	Human Alignment	Percent of viral reads	Adjacent normal tissue (control)	Excluded from DE testing after QC
**1**	16	48	156662061	97.05%	0.00000	yes	yes
**2**	16	93	183209234	97.04%	0.00000	yes	yes
**3**	**16**	4224	135420123	95.95%	0.00006	yes	
**4**	33	47	228314296	95.30%	0.00000	yes	
**5**	33	192	135829214	91.44%	0.00000	yes	
**6**	33	685	175502817	96.46%	0.00001	yes	
**7**			264489652	94.74%	0.00000	yes	yes
**8**			191341977	94.87%	0.00000	yes	yes
**9**			191752178	96.89%	0.00000	yes	yes
**10**			179414488	95.97%	0.00000	yes	yes
**11**			167528946	94.75%	0.00000	yes	yes
**12**			128726438	97.71%	0.00000	yes	yes
**13**			124826871	97.43%	0.00000	yes	yes
**14**			203759845	97.65%	0.00000	yes	yes
**15**			139606583	96.28%	0.00000	yes	yes
**16**			175301902	97.42%	0.00000	yes	yes
**17**			153008707	97.59%	0.00000	yes	yes
**18**			166508597	97.13%	0.00000	yes	yes
**19**			187420745	97.37%	0.00000	yes	yes
**20**			176564229	93.72%	0.00000	yes	yes
**21**			106362333	77.73%	0.00000	yes	yes
**42**			174223806	96.94%	0.00000	yes	yes
**22**			149871285	98.04%	0.00000	yes	
**23**			180655882	96.88%	0.00000	yes	
**24**			118580777	97.73%	0.00000	yes	
**25**			187122111	97.43%	0.00000	yes	
**26**			193507320	97.19%	0.00000	yes	
**27**			134812711	97.02%	0.00000	yes	
**28**			162709556	96.93%	0.00000	yes	
**29**			157650749	96.51%	0.00000	yes	
**30**			200731599	97.58%	0.00000	yes	
**31**			169044377	98.35%	0.00000	yes	
**32**			183108273	95.86%	0.00000	yes	
**33**			172148456	95.54%	0.00000	yes	

**34**			184455131	95.60%	0.00000	yes	
**35**			161970033	96.62%	0.00000	yes	
**36**			174666325	96.49%	0.00000	yes	
**37**			224686877	96.49%	0.00000	yes	
**38**			196617441	97.93%	0.00000	yes	
**39**			107287022	97.04%	0.00000	yes	
**40**			116499851	90.72%	0.00000	yes	
**41**			134756781	93.87%	0.00000	yes	
**43**	16	48	221965196	95.87%	0.00000		
**44**	16	48	151680872	97.05%	0.00000		
**45**	16	48	101701001	95.71%	0.00000		
**46**	16	94	139873114	96.51%	0.00000		
**47**	16	95	142869850	96.85%	0.00000		
**48**	16	96	212726172	82.81%	0.00000		yes
**49**	16	96	161546408	97.87%	0.00000		
**50**	16	96	192585460	98.08%	0.00000		
**51**	16	96	142044075	97.33%	0.00000		
**52**	16	96	145549751	98.19%	0.00000		
**53**	16	96	148260067	96.16%	0.00000		
**54**	16	96	105274148	97.76%	0.00000		
**55**	16	96	175466531	96.52%	0.00000		
**56**	16	96	135853820	96.63%	0.00000		
**57**	16	96	141704467	97.24%	0.00000		
**58**	16	96	210654444	95.24%	0.00000		
**59**	16	96	197580122	98.19%	0.00000		
**60**	16	96	159343690	97.87%	0.00000		
**61**	16	96	170657702	97.96%	0.00000		
**62**	16	96	211258133	96.48%	0.00000		
**63**	16	142	129633416	97.60%	0.00000		
**64**	16	144	211235932	97.13%	0.00000		
**65**	16	184	131719740	95.36%	0.00000		
**66**	16	192	185676431	82.77%	0.00000		yes
**67**	16	225	91088897	93.90%	0.00001		
**68**	16	227	189659553	91.99%	0.00000		yes
**69**	16	233	171832089	97.13%	0.00000		
**70**	16	238	169642939	95.45%	0.00000		
**71**	16	285	147855568	98.11%	0.00000		
**72**	16	288	157250967	97.76%	0.00000		
**73**	16	365	180801783	96.06%	0.00000		yes
**74**	16	373	146525687	96.82%	0.00001		
**75**	16	374	190014451	97.75%	0.00000		
**76**	16	375	134031237	97.42%	0.00001		
**77**	16	380	121972453	90.18%	0.00001		

**78**	16	382	134207318	88.32%	0.00001		
**79**	16	400	172227397	95.64%	0.00000		
**80**	16	427	150054175	97.32%	0.00001		yes
**81**	16	432	190985442	97.09%	0.00000		
**82**	16	453	140360257	97.36%	0.00001		
**83**	16	490	105638143	90.31%	0.00001		
**84**	16	525	87084249	83.95%	0.00001		yes
**85**	16	561	203584550	96.46%	0.00001		
**86**	16	569	229039288	95.75%	0.00001		
**87**	16	594	212684122	98.24%	0.00001		
**88**	16	613	169351712	96.82%	0.00001		
**89**	16	657	158378775	97.80%	0.00001		
**90**	16	671	116600297	95.95%	0.00001		
**91**	16	685	172545016	97.92%	0.00001		
**92**	16	747	163040495	97.99%	0.00001		
**93**	16	840	188839823	97.79%	0.00001		
**94**	16	857	158547694	96.98%	0.00001		
**95**	16	881	151411167	97.27%	0.00001		
**96**	16	911	252236977	97.54%	0.00001		
**97**	16	987	163382086	95.70%	0.00001		
**98**	16	1141	168341694	97.85%	0.00001		
**99**	16	1239	143134473	96.18%	0.00002		
**100**	16	1427	142515402	97.19%	0.00002		
**101**	16	1564	134853821	93.99%	0.00002		
**102**	16	1728	154404696	96.92%	0.00002		
**103**	16	2400	154541053	97.13%	0.00003		
**104**	16	2512	138087008	97.43%	0.00004		
**105**	16	2621	182198647	97.90%	0.00003		
**106**	16	2802	169804704	97.18%	0.00003		
**107**	16	3049	139292062	97.13%	0.00005		
**108**	16	4718	223043128	98.19%	0.00004		
**109**	16	5674	182325298	97.85%	0.00006		
**110**	16	18359	130409385	93.61%	0.00029		
**111**	16	226461	117593724	93.95%	0.00401		
**112**	16	265119	181800677	98.24%	0.00304		
**113**	16	314444	186541338	96.71%	0.00351		
**114**	16	364105	138798066	97.33%	0.00547		
**115**	16	384506	108105933	97.26%	0.00741		
**116**	16	485210	126558099	96.60%	0.00799		
**117**	16	505558	157848843	95.64%	0.00667		
**118**	16	787416	128714868	97.09%	0.01274		
**119**	16	853482	152055374	96.82%	0.01169		
**120**	16	855348	141069328	95.79%	0.01263		

**121**	16	882290	152324480	97.03%	0.01207		
**122**	16	960513	133648016	96.62%	0.01497		
**123**	16	972234	87267636	87.72%	0.02321		
**124**	16	1006690	175832359	98.05%	0.01193		
**125**	16	1258409	169192429	93.98%	0.01550		
**126**	16	1471944	110476476	90.16%	0.02776		
**127**	16	1473414	189160690	96.84%	0.01623		
**128**	16	1680370	179449426	97.21%	0.01951		
**129**	16	1720472	196116441	96.40%	0.01828		
**130**	16	1730798	209602192	96.54%	0.01720		
**131**	16	1783035	133666734	97.01%	0.02779		
**132**	16	1866060	126227402	97.34%	0.03080		
**133**	16	1931535	123855627	95.93%	0.03249		
**134**	16	1981818	134881524	98.02%	0.03061		
**135**	16	2396972	170181479	90.60%	0.02934		yes
**136**	16	2503708	131001786	96.86%	0.03982		
**137**	16	2632360	125337742	97.65%	0.04375		
**138**	16	2632802	176148173	95.56%	0.03114		
**139**	16	2733591	120258638	92.79%	0.04736		
**140**	16	2739084	112957134	95.44%	0.05052		
**141**	16	2796969	213770639	97.12%	0.02726		
**142**	16	2799463	166349755	95.15%	0.03506		
**143**	16	2801223	205368171	93.83%	0.02842		
**144**	16	2925374	87755308	96.82%	0.06945		
**145**	16	2942341	187881792	96.29%	0.03263		
**146**	16	3203224	135647829	97.36%	0.04920		
**147**	16	3244557	171702395	96.51%	0.03937		
**148**	16	3537258	174622214	96.55%	0.04220		
**149**	16	3602418	203222827	97.89%	0.03693		
**150**	16	3677344	141611111	94.84%	0.05410		
**151**	16	3983053	181402697	96.76%	0.04574		
**152**	16	4058158	133788085	97.33%	0.06319		yes
**153**	16	4195126	192128749	96.14%	0.04549		
**154**	16	4375981	140136870	96.82%	0.06506		
**155**	16	4441212	116818303	97.24%	0.07920		
**156**	16	4508181	145014082	97.20%	0.06477		
**157**	16	4808667	192875478	96.50%	0.05194		yes
**158**	16	4898480	146217231	97.58%	0.06979		
**159**	16	5062862	169621351	97.36%	0.06218		
**160**	16	5070544	153661147	95.93%	0.06875		yes
**161**	16	5181539	154863734	96.70%	0.06971		
**162**	16	5912623	127205489	96.86%	0.09684		
**163**	16	6197052	142362280	96.58%	0.09069		

**164**	16	6640931	154200193	96.86%	0.08972		
**165**	16	7352887	172579907	97.18%	0.08876		
**166**	16	9058344	146638390	96.67%	0.12869		
**167**	16	9752604	191029843	97.84%	0.10636		
**168**	18	100	199208971	96.09%	0.00000		
**169**	18	148	169527999	96.45%	0.00000		
**170**	18	150	188789633	96.15%	0.00000		
**171**	18	191	186956454	97.22%	0.00000		
**172**	18	197	173217078	97.27%	0.00000		
**173**	18	200	192636290	96.28%	0.00000		
**174**	18	200	197884997	94.14%	0.00000		
**175**	18	240	239597166	96.02%	0.00000		
**176**	18	250	212872271	95.85%	0.00000		
**177**	18	299	176802000	96.09%	0.00000		
**178**	18	347	174215218	96.62%	0.00000		
**179**	18	442	153963289	96.17%	0.00001		
**180**	18	500	180455061	96.03%	0.00001		
**181**	18	549	188387980	96.02%	0.00001		
**182**	18	598	154175542	96.76%	0.00001		
**183**	18	638	265547357	96.05%	0.00001		
**184**	18	743	186968761	96.05%	0.00001		
**185**	33	100	186428242	96.92%	0.00000		
**186**	33	192	168356882	97.35%	0.00000		
**187**	33	250	190143231	96.90%	0.00000		
**188**	33	283	175163754	95.14%	0.00000		
**189**	33	1008	215273850	97.87%	0.00001		
**190**	33	1525	156339709	96.29%	0.00002		
**191**	33	2674	154429487	94.19%	0.00004		
**192**	33	4188	179402532	95.49%	0.00005		
**193**	33	1662079	113154121	88.87%	0.03060		yes
**194**	33	2274478	160888754	97.22%	0.02945		yes
**195**	33	2399524	174652904	95.24%	0.02862		
**196**	33	2931710	**133959420**	93.00%	0.04559		
**197**	33	4559877	**119640891**	95.99%	0.07940		
**198**	33	5476946	184553017	95.12%	0.06183		
**199**	33	15302781	249557396	93.59%	0.12775		
**200**	33	26329862	165321411	96.38%	0.33180		
**201**	35	310	110119029	96.59%	0.00001		
**202**	35	860	160837131	96.96%	0.00001		
**203**	35	324468	109890203	96.48%	0.00615		
**204**	35	2983612	152683357	97.11%	0.04071		
**205**	35	6633817	150348250	95.35%	0.09192		
**206**			193043648	97.62%	0.00000		yes

**207**			117082337	81.89%	0.00000		yes
**208**			218635200	53.16%	0.00000		yes
**209**			182050655	97.23%	0.00000		yes
**210**			195616016	95.66%	0.00000		yes
**211**			145626005	97.32%	0.00000		yes
**212**			225456182	96.38%	0.00000		yes
**213**			243147682	96.28%	0.00000		yes
**214**			208281687	93.35%	0.00000		yes
**215**			115261403	97.88%	0.00000		yes
**216**			88498063	97.11%	0.00000		yes
**217**			246283798	97.65%	0.00000		yes
**218**			148227421	92.91%	0.00000		yes
**219**			143124021	96.45%	0.00000		yes
**220**			226245434	96.88%	0.00000		yes
**221**			140205662	97.25%	0.00000		yes
**222**			129880074	97.67%	0.00000		yes
**223**			185853671	96.52%	0.00000		yes
**224**			186735843	97.25%	0.00000		yes
**225**			130135816	97.27%	0.00000		yes
**226**			159058344	97.04%	0.00000		yes
**227**			155340990	97.50%	0.00000		yes
**228**			173445755	96.97%	0.00000		yes
**229**			180031740	97.06%	0.00000		yes
**230**			181215309	97.58%	0.00000		yes
**231**			190560974	97.55%	0.00000		yes
**232**			218715316	96.43%	0.00000		yes
**233**			184303808	95.35%	0.00000		yes
**234**			194917787	93.94%	0.00000		yes
**235**			153926599	96.72%	0.00000		yes
**236**			145491789	94.41%	0.00000		yes
**237**			146551423	97.08%	0.00000		yes
**238**			144314908	87.47%	0.00000		yes
**239**			173710092	89.24%	0.00000		yes
**240**			146933664	93.47%	0.00000		yes
**241**			138296731	93.26%	0.00000		yes
**242**			144628259	83.15%	0.00000		yes
**243**			95933974	95.54%	0.00000		yes
**244**			172209613	96.53%	0.00000		yes
**245**			103770791	73.50%	0.00000		yes
**246**			170544626	94.48%	0.00000		yes
**247**			220928619	94.61%	0.00000		
**248**			133211815	97.47%	0.00000		
**249**			130063537	97.48%	0.00000		

**250**			253433256	96.07%	0.00000		
**251**			232720344	94.85%	0.00000		
**252**			187031083	97.73%	0.00000		
**253**			189609587	97.79%	0.00000		
**254**			195428035	97.87%	0.00000		
**255**			182728794	97.57%	0.00000		
**256**			240570141	97.35%	0.00000		
**257**			119930294	97.08%	0.00000		
**258**			98947667	96.52%	0.00000		
**259**			184960562	97.01%	0.00000		
**260**			130485974	89.55%	0.00000		
**261**			58397504	97.47%	0.00000		
**262**			136923233	95.71%	0.00000		
**263**			171830779	97.17%	0.00000		
**264**			144860215	96.55%	0.00000		
**265**			176195735	97.60%	0.00000		
**266**			155590514	95.54%	0.00000		
**267**			171077434	96.36%	0.00000		
**268**			185485615	93.91%	0.00000		
**269**			94682866	94.13%	0.00000		
**270**			105300428	95.86%	0.00000		
**271**			172288037	97.42%	0.00000		
**272**			149845790	97.65%	0.00000		
**273**			132494403	96.12%	0.00000		
**274**			141145130	95.33%	0.00000		
**275**			158748052	96.48%	0.00000		
**276**			219705243	96.21%	0.00000		
**277**			136265898	97.82%	0.00000		
**278**			124955257	98.22%	0.00000		
**279**			160828604	95.32%	0.00000		
**280**			175257788	97.94%	0.00000		
**281**			151043991	98.06%	0.00000		
**282**			178223366	97.80%	0.00000		
**283**			197742553	97.26%	0.00000		
**284**			192691700	97.66%	0.00000		
**285**			163482365	96.54%	0.00000		
**286**			124913695	98.05%	0.00000		
**287**			202895880	96.33%	0.00000		
**288**			158645262	97.97%	0.00000		
**289**			182852509	97.69%	0.00000		
**290**			119475601	98.21%	0.00000		
**291**			152709292	97.35%	0.00000		
**292**			156450760	97.81%	0.00000		

**293**			136447404	98.16%	0.00000		
**294**			128812790	96.56%	0.00000		
**295**			143194125	97.71%	0.00000		
**296**			126477418	98.03%	0.00000		
**297**			147531683	97.83%	0.00000		
**298**			176247350	97.75%	0.00000		
**299**			276932304	97.93%	0.00000		
**300**			156456853	98.22%	0.00000		
**301**			198938266	97.67%	0.00000		
**302**			196964519	96.98%	0.00000		
**303**			237678439	97.26%	0.00000		
**304**			141681042	97.25%	0.00000		
**305**			158045676	97.03%	0.00000		
**306**			181049801	96.32%	0.00000		
**307**			257001145	96.99%	0.00000		
**308**			219928181	96.96%	0.00000		
**309**			122403769	96.54%	0.00000		
**310**			146107356	96.44%	0.00000		
**311**			142265713	97.69%	0.00000		
**312**			155600213	97.18%	0.00000		
**313**			169692333	95.68%	0.00000		
**314**			172691203	97.50%	0.00000		
**315**			143143618	97.20%	0.00000		
**316**			177774031	95.72%	0.00000		
**317**			114792332	97.87%	0.00000		
**318**			105169864	97.95%	0.00000		
**319**			124667337	98.08%	0.00000		
**320**			108775990	91.40%	0.00000		
**321**			125932736	97.80%	0.00000		
**322**			182997857	98.13%	0.00000		
**323**			161078918	97.90%	0.00000		
**324**			143729915	97.00%	0.00000		
**325**			162525920	94.56%	0.00000		
**326**			161153935	96.49%	0.00000		
**327**			222817086	96.96%	0.00000		
**328**			197900983	96.73%	0.00000		
**329**			212697322	98.46%	0.00000		
**330**			200536390	97.74%	0.00000		
**331**			194268436	98.20%	0.00000		
**332**			205690237	98.04%	0.00000		
**333**			116231015	96.84%	0.00000		
**334**			167319386	95.95%	0.00000		
**335**			190458770	95.60%	0.00000		

**336**			103370422	96.58%	0.00000		
**337**			144311708	96.29%	0.00000		
**338**			158641352	96.59%	0.00000		
**339**			152759762	93.59%	0.00000		
**340**			125123456	96.21%	0.00000		
**341**			122464836	97.07%	0.00000		
**342**			125058897	94.03%	0.00000		
**343**			127276437	96.72%	0.00000		
**344**			130693758	96.73%	0.00000		
**345**			155955895	97.17%	0.00000		
**346**			148893307	97.46%	0.00000		
**347**			146526393	97.33%	0.00000		
**348**			133460767	97.85%	0.00000		
**349**			201630720	95.72%	0.00000		
**350**			196925317	97.28%	0.00000		
**351**			158941841	96.82%	0.00000		
**352**			188396673	96.81%	0.00000		
**353**			219651302	96.01%	0.00000		
**354**			175230242	96.97%	0.00000		
**355**			167667593	96.50%	0.00000		
**356**			176294339	96.75%	0.00000		
**357**			169154897	97.04%	0.00000		
**358**			179239223	97.70%	0.00000		
**359**			18661720	100%	0.00000		
**360**			178286758	97.48%	0.00000		
**361**			129693889	97.29%	0.00000		
**362**			120291663	95.78%	0.00000		
**363**			177468936	96.54%	0.00000		
**364**			171125920	97.49%	0.00000		
**365**			169371572	97.10%	0.00000		
**366**			141691113	96.26%	0.00000		
**367**			108989404	97.34%	0.00000		
**368**			95514595	97.05%	0.00000		
**369**			135857130	96.70%	0.00000		
**370**			158194630	97.60%	0.00000		
**371**			196786737	97.37%	0.00000		
**372**			156914492	97.55%	0.00000		
**373**			114745455	97.10%	0.00000		
**374**			146989719	96.91%	0.00000		
**375**			106194025	98.20%	0.00000		
**376**			167923107	97.31%	0.00000		
**377**			159776020	97.08%	0.00000		
**378**			133269063	96.93%	0.00000		

**379**			124906754	96.45%	0.00000		
**380**			146751730	95.94%	0.00000		
**381**			173656088	97.28%	0.00000		
**382**			202731863	96.93%	0.00000		
**383**			154528698	98.09%	0.00000		
**384**			170319716	94.99%	0.00000		
**385**			122105120	95.94%	0.00000		
**386**			233293705	95.80%	0.00000		
**387**			198964913	96.06%	0.00000		
**388**			235653425	97.55%	0.00000		
**389**			159653375	97.03%	0.00000		
**390**			167974428	97.92%	0.00000		
**391**			206419552	95.24%	0.00000		
**392**			153215688	97.40%	0.00000		
**393**			209630221	96.96%	0.00000		
**394**			168005469	97.66%	0.00000		
**395**			185363677	97.60%	0.00000		
**396**			170659040	97.29%	0.00000		
**397**			189713526	97.36%	0.00000		
**398**			155018672	97.53%	0.00000		
**399**			171002904	97.19%	0.00000		
**400**			91635912	97.71%	0.00000		
**401**			209295725	97.23%	0.00000		
**402**			143153025	97.61%	0.00000		
**403**			167702900	97.26%	0.00000		
**404**			231711423	96.33%	0.00000		
**405**			239792403	97.65%	0.00000		
**406**			132923747	97.35%	0.00000		
**407**			199954355	97.62%	0.00000		
**408**			177767912	98.10%	0.00000		
**409**			191052222	95.28%	0.00000		
**410**			204523813	94.89%	0.00000		
**411**			187213089	94.90%	0.00000		
**412**			206862346	94.92%	0.00000		
**413**			187290055	98.02%	0.00000		
**414**			195087341	97.85%	0.00000		
**415**			165727582	98.26%	0.00000		
**416**			199790329	95.93%	0.00000		
**417**			167799591	97.03%	0.00000		
**418**			167113372	98.11%	0.00000		
**419**			176253226	98.17%	0.00000		
**420**			190990804	96.09%	0.00000		
**421**			187077304	96.54%	0.00000		

**422**			178859537	96.70%	0.00000		
**423**			169759512	97.83%	0.00000		
**424**			140170419	96.72%	0.00000		
**425**			153249306	97.35%	0.00000		
**426**			134269877	97.29%	0.00000		
**427**			142887166	94.05%	0.00000		
**428**			136420781	95.47%	0.00000		
**429**			131352300	96.30%	0.00000		
**430**			133785409	96.52%	0.00000		
**431**			131286345	92.25%	0.00000		
**432**			148888536	96.36%	0.00000		
**433**			139253044	96.29%	0.00000		
**434**			139331367	97.03%	0.00000		
**435**			151979906	97.33%	0.00000		
**436**			133816189	96.31%	0.00000		
**437**			146488826	96.50%	0.00000		
**438**			140627681	95.52%	0.00000		
**439**			154778031	89.93%	0.00000		
**440**			127968943	96.67%	0.00000		
**441**			193673821	96.60%	0.00000		
**442**			154946476	88.73%	0.00000		
**443**			191363068	97.18%	0.00000		
**444**			168273139	95.68%	0.00000		
**445**			194259952	97.57%	0.00000		
**446**			194605748	97.09%	0.00000		
**447**			137787824	92.55%	0.00000		
**448**			159064887	96.50%	0.00000		
**449**			188636593	98.08%	0.00000		
**450**			**204895428**	98.05%	0.00000		
**451**			230737736	97.85%	0.00000		
**452**			163706789	97.51%	0.00000		
**453**			172138714	97.61%	0.00000		
**454**			240047291	91.33%	0.00000		
**455**			177445809	97.24%	0.00000		
**456**			96930772	94.46%	0.00000		
**457**			94401169	96.43%	0.00000		
**458**			138405295	97.22%	0.00000		
**459**			125481856	96.37%	0.00000		
**460**			120512376	93.65%	0.00000		
**461**			120394182	93.95%	0.00000		
**462**			125287701	94.50%	0.00000		
**463**			188421588	95.70%	0.00000		
**464**			141784654	93.54%	0.00000		

**465**			170335688	96.08%	0.00000		
**466**			211831650	98.01%	0.00000		
**467**			169038450	98.09%	0.00000		
**468**			152225579	98.33%	0.00000		
**469**			146311650	97.95%	0.00000		
**470**			245260754	98.03%	0.00000		
**471**			165391849	97.36%	0.00000		
**472**			97677467	96.92%	0.00000		
**473**			112978653	93.76%	0.00000		
**474**			146797249	96.19%	0.00000		
**475**			101228351	97.52%	0.00000		
**476**			120906908	96.63%	0.00000		
**477**			102620892	93.82%	0.00000		
**478**			131260800	97.24%	0.00000		
**479**			129081175	97.07%	0.00000		
**480**			138724968	94.85%	0.00000		
**481**			142240105	97.22%	0.00000		
**482**			166365274	97.31%	0.00000		
**483**			177258391	96.64%	0.00000		
**484**			204439220	96.61%	0.00000		
**485**			95993217	96.27%	0.00000		
**486**			100401192	96.88%	0.00000		
**487**			159091371	96.93%	0.00000		
**488**			156896235	96.91%	0.00000		
**489**			121361971	97.70%	0.00000		
**490**			95463372	96.95%	0.00000		
**491**			122860168	95.91%	0.00000		
**492**			151242483	97.59%	0.00000		
**493**			119950904	90.62%	0.00000		
**494**			163363229	97.85%	0.00000		
**495**			150091911	97.70%	0.00000		
**496**			137269042	96.91%	0.00000		
**497**			157877791	96.45%	0.00000		
**498**			137711365	95.95%	0.00000		
**499**			146819242	96.64%	0.00000		
**500**			193375748	96.45%	0.00000		
**501**			144113376	94.75%	0.00000		
**502**			144237858	96.98%	0.00000		
**503**			174092067	96.20%	0.00000		
**504**			131384719	93.45%	0.00000		
**505**			143988928	96.97%	0.00000		
**506**			142410944	97.18%	0.00000		
**507**			99470578	96.76%	0.00000		

**508**			152654073	96.08%	0.00000		
**509**			94418324	89.17%	0.00000		
**510**			126834221	95.67%	0.00000		
**511**			157942831	97.83%	0.00000		
**512**			150990002	97.92%	0.00000		
**513**			180388482	97.17%	0.00000		
**514**			152544943	94.75%	0.00000		
**515**			133183920	96.25%	0.00000		
**516**			144256065	96.56%	0.00000		
**517**			164093610	97.59%	0.00000		
**518**			138892084	95.27%	0.00000		
**519**			165571311	93.83%	0.00000		
**520**			142550580	87.22%	0.00000		
**521**			191959511	94.50%	0.00000		
**522**			138092317	91.67%	0.00000		
**523**			97004019	96.73%	0.00000		
**524**			168758967	95.36%	0.00000		
**525**			198882142	96.40%	0.00000		
**526**			155121111	95.59%	0.00000		
**527**			125225693	89.07%	0.00000		
**528**			82863787	95.16%	0.00000		
**529**			157914397	96.04%	0.00000		
**530**			171769310	95.19%	0.00000		
**531**			105319190	96.59%	0.00000		
**532**			103542497	95.88%	0.00000		
**533**			120381340	92.45%	0.00000		
**534**			106410636	95.39%	0.00000		
**535**			131587007	96.94%	0.00000		
**536**			151970470	95.55%	0.00000		
**537**			82441118	94.54%	0.00000		
