# Supplementary material for: Non-coding RNAs profiling in head and neck cancers
Source: NPJ Genom Med. 2016 Jan 13;1:15004–. doi: 10.1038/npjgenmed.2015.4 (PMC5685291; doi:10.1038/npjgenmed.2015.4)
Supplement: Supplemental Table 3 [file npjgenmed20154-s3.pdf]

Supplemental table 3.: Anatomical sites

HPV16 expression	Alveolar Ridge	Base of tongue	Buccal mucosa	Floor of mouth	Hard palate	Hypopharynx	Larynx	Lip	Oral cavity	Tongue	Oropharynx	Tonsil	Total sum
HPV16+ tumors	4	13	0	0	1	2	1	0	3	2	0	28	54
HPV16 low tumors	2	2	2	8	2	1	11	0	12	9	2	1	52
HPV- tumors	10	8	16	43	2	4	74	3	40	82	2	5	289
Adjacent normal tissue (controls)	0	0	0	2	0	0	7	0	10	5	0	0	24
