# Supplementary material for: Non-coding RNAs profiling in head and neck cancers
Source: NPJ Genom Med. 2016 Jan 13;1:15004–. doi: 10.1038/npjgenmed.2015.4 (PMC5685291; doi:10.1038/npjgenmed.2015.4)
Supplement: Supplemental Table 4 [file npjgenmed20154-s4.pdf]

Supplemental table 4.: Follow-up ANOVA analysis on significantly DE genes in tonsil or base of tongue *versus* other anatomical sites

Gene name	t-test for DE in virus free tonsils or base of tongue vs. other anatomical sites		Follow-up ANOVA in HNSC cohort for DE in tonsil or base of tongue vs. other anatomical sites		Confounding effect of anatomical site on association of gene expression with HPV16 status	Biotype
	Fold Change	Adjusted p-value	p-value, anatomic site	p-value, HPV16		
HMGN2P18	-3.296509	**1.06E-04**	**1.19E-05**	2.19E-01	Confounded	processed_pseudogene
SCGB1A1	2.780568	**4.01E-13**	3.97E-01	**4.13E-02**	Not confounded	protein_coding
MIRLET7I	1.346814	**3.91E-03**	7.90E-01	7.49E-01	Not confounded	miRNA
RP11-242D8.2	3.303077	**4.72E-15**	2.56E-01	**1.72E-02**	Not confounded	unprocessed_pseudogene
MIR4263	-1.79048	**2.59E-02**	2.78E-01	5.49E-02	Not confounded	miRNA
RNU6-1161P	-2.367232	**2.64E-03**	8.18E-02	**1.57E-02**	Not confounded	snRNA
RP4-613B23.1	-1.854595	**1.68E-02**	7.34E-01	1.77E-01	Not confounded	antisense
RNU1-70P	1.945099	**6.76E-07**	2.29E-01	9.84E-01	Not confounded	snRNA
HSPB1P1	-2.158883	**4.47E-04**	**3.53E-02**	4.69E-01	Not confounded	processed_pseudogene
