# Supplementary material for: Non-coding RNAs profiling in head and neck cancers
Source: NPJ Genom Med. 2016 Jan 13;1:15004–. doi: 10.1038/npjgenmed.2015.4 (PMC5685291; doi:10.1038/npjgenmed.2015.4)
Supplement: Supplemental Table 5 [file npjgenmed20154-s5.pdf]

Supplemental table 5.: Association of HPV16 load with histological composition of tumor

Type of infiltration	p-values for association with HPV16 read count
percent_lymphocyte_infiltration	0.60
percent_monocyte_infiltration	0.65
percent_necrosis	0.69
percent_neutrophil_infiltration	0.43
percent_normal_cells	0.91
percent_stromal_cells	0.67
percent_tumor_cells	0.87
percent_tumor_nuclei	0.22
