# Supplementary material for: Non-coding RNAs profiling in head and neck cancers
Source: NPJ Genom Med. 2016 Jan 13;1:15004–. doi: 10.1038/npjgenmed.2015.4 (PMC5685291; doi:10.1038/npjgenmed.2015.4)
Supplement: Supplemental Table 6 [file npjgenmed20154-s6.pdf]

**Supplemental table 6: Genes with significant expression changes between tumors and controls chaning in opposite direction for HPV+ and HPV- samples**

| Gene name | Biotype | Differential expression (log2 ratio) | | | Association with cancer | # of papers associated with cancer* |
|---|---|---|---|---|---|---|
| | | HPV16+ vs. HPV- | HPV16+ vs. controls | HPV16-/low vs. controls | | |
| ***YBX2*** | protein coding | 5.61 | **2.87** | **-2.70** | NA | 0 |
| ***MYO3A*** | protein coding | 6.52 | **4.24** | **-2.26** | associated with multiple cancers | 4 |
| ***TDRD10*** | protein coding | 3.81 | **2.04** | **-1.72** | NA | 0 |
| ***NEFH*** | protein coding | 5.23 | **3.50** | **-1.66** | associated with multiple cancers | 8 |
| ***CD8B*** | protein coding | 3.55 | **2.52** | **-1.00** | ductal carcinoma, melanoma | 3 |
| ***SORCS2*** | protein coding | -3.20 | **-2.15** | **1.07** | melanoma | 1 |
| ***HSPA12A*** | protein coding | -2.82 | **-1.76** | **1.09** | NA | 0 |
| ***GDPD2*** | protein coding | -2.31 | **-1.26** | **1.11** | NA | 0 |
| ***NT5E*** | protein coding | -2.45 | **-1.34** | **1.12** | associated with multiple cancers | 19 |
| ***RP11-259O2.1*** | lincRNA | -3.72 | **-2.50** | **1.15** | NA | 0 |
| ***CXCL14*** | protein coding | -3.85 | **-2.71** | **1.18** | associated with multiple cancers | 50 |
| ***KIRREL*** | protein coding | -2.38 | **-1.12** | **1.29** | NA | 0 |
| ***SLC4A3*** | protein coding | -2.79 | **-1.43** | **1.34** | hepatocellular carcinoma | 1 |
| ***CSPG4*** | protein coding | -2.71 | **-1.34** | **1.40** | associated with multiple cancers | 26 |
| ***F2RL1*** | protein coding | -3.68 | **-2.32** | **1.40** | thyroid cancer and mesothelioma | 3 |
| ***CAV1*** | protein coding | -2.96 | **-1.50** | **1.50** | associated with multiple cancers | 130 |
| ***THSD1*** | protein coding | -2.84 | **-1.35** | **1.55** | colorectal cancer, esophageal carcinoma | 2 |
| ***AJAP1*** | protein coding | -3.64 | **-2.09** | **1.56** | silenced in gloioma and glioblastoma, shorter survival in glioma, methylated in EBV positive gastric cancer | 9 |
| ***SPOCK1*** | protein coding | -3.95 | **-2.43** | **1.57** | promotes lung, prostate, bladder cancer | 7 |
| ***VEGFC*** | protein coding | -2.92 | **-1.24** | **1.71** | associated with multiple cancers | 64 |
| ***SLC6A2*** | protein coding | -3.66 | **-1.92** | **1.72** | NA | 0 |
| ***RP11-497E19.2*** | protein coding | -4.57 | **-2.33** | **2.21** | NA | 0 |
| ***COL4A6*** | protein coding | -6.25 | **-3.31** | **2.94** | silenced in prostate and colorectal cancer | 3 |

*Footnote:*

* - scicurve.com/ was used for paper search

NA - has not been previously shown in association with cancer
