# Supplementary material for: Non-coding RNAs profiling in head and neck cancers
Source: NPJ Genom Med. 2016 Jan 13;1:15004–. doi: 10.1038/npjgenmed.2015.4 (PMC5685291; doi:10.1038/npjgenmed.2015.4)
Supplement: Supplemental Table 7 [file npjgenmed20154-s7.pdf]

Supplemental table 7: Number of genes differentially expressed between HNSC tumors and normal adjacent tissue as well as between HNSC tumors stratified by HPV16 status

Type of RNA	Tumors vs. controls, UP-regulated genes	Tumors vs. controls, DOWN-regulated genes	HPV16+ tumors vs. HPV16- tumors, UP-regulated genes	HPV16+ tumors vs. HPV- tumors, DOWN-regulated genes	Percentage from all expressed genes	Percentage from all DE genes
protein coding	404	699	728	852	68.41%	63.87%
antisense	95	84	214	139	11.13%	12.66%
long non-coding (lncRNA)	123	70	218	179	11.42%	14.04%
pseudogenes	42	28	128	59	4.91%	6.12%
short RNA	27	5	92	15	4.14%	3.31%
