# Supplementary material for: Non-coding RNAs profiling in head and neck cancers
Source: NPJ Genom Med. 2016 Jan 13;1:15004–. doi: 10.1038/npjgenmed.2015.4 (PMC5685291; doi:10.1038/npjgenmed.2015.4)
Supplement: Supplemental Table 8 [file npjgenmed20154-s8.pdf]

Supplemental table 8: Differentially expressed non-coding RNA genes with corresponding potential protein-coding regulatory target genes

ncRNA name	target gene name	ncRNA biotype	target.gene.biotype	ncRNA log2 fold change	target gene log2 fold change	test category
RP11-677I18.3	*AASDHPPT*	antisense	protein_coding	-1.11	-0.93	HPV16+/HPV16-
RP11-218E20.5	*ABHD12B*	antisense	protein_coding	-2.90	-1.78	HPV16+/HPV16-
SNRK-AS1	*ABHD5*	antisense	protein_coding	-1.28	-1.20	tumors/normal
LINC01237	*AC131097.4*	antisense	protein_coding	1.62	2.08	HPV16+/HPV16-
RP11-144G7.2	*ACAT1*	antisense	protein_coding	-1.25	-0.82	tumors/normal
RP11-275I14.4	*ACBD3*	antisense	protein_coding	-1.75	-0.58	tumors/normal
RP11-624C23.1	*ADAM28*	antisense	protein_coding	1.87	2.39	HPV16+/HPV16-
RP11-624C23.1	*ADAMDEC1*	antisense	protein_coding	1.87	1.55	HPV16+/HPV16-
CTB-25B13.9	*ADAMTSL5*	antisense	protein_coding	-1.49	-1.13	HPV16+/HPV16-
AC091177.1	*ADAP2*	antisense	protein_coding	-1.74	-0.79	HPV16+/HPV16-
RP11-443B20.1	*ADCY3*	antisense	protein_coding	1.84	0.94	tumors/normal
TMEM220-AS1	*ADPRM*	antisense	protein_coding	-2.56	-0.85	tumors/normal
RP11-360L9.4	*AGPAT6*	antisense	protein_coding	1.16	0.31	HPV16+/HPV16-
RP11-360L9.7	*AGPAT6*	antisense	protein_coding	1.61	0.13	tumors/normal
RP11-337N6.1	*AGPS*	antisense	protein_coding	2.12	0.51	tumors/normal
RP3-388E23.2	*AHI1*	antisense	protein_coding	1.47	0.38	HPV16+/HPV16-
RP11-67C2.2	*ALOX5*	antisense	protein_coding	1.42	0.82	HPV16+/HPV16-

RP13-225O21.2	*ANGPTL2*	antisense	protein_coding	2.38	-0.91	HPV16+/HPV16-
RP11-388P9.2	*ANK3*	antisense	protein_coding	-1.96	-1.42	tumors/normal
AC137932.5	*ANKRD11*	antisense	protein_coding	1.40	-0.19	HPV16+/HPV16-
RP3-436N22.3	*ANKRD45*	antisense	protein_coding	3.78	0.55	tumors/normal
RP4-758J18.7	*ANKRD65*	antisense	protein_coding	1.16	1.29	HPV16+/HPV16-
ANO1-AS1	*ANO1*	antisense	protein_coding	-2.61	-2.29	HPV16+/HPV16-
SNRK-AS1	*ANO10*	antisense	protein_coding	-1.28	-0.54	tumors/normal
CTD-2035E11.3	*ANXA2R*	antisense	protein_coding	1.29	0.74	HPV16+/HPV16-
AC006942.4	*AP2A1*	antisense	protein_coding	1.00	0.09	HPV16+/HPV16-
RP11-829H16.3	*AP4S1*	antisense	protein_coding	1.83	-0.22	HPV16+/HPV16-
RP11-632F7.3	*APBB2*	antisense	protein_coding	-2.20	-1.87	HPV16+/HPV16-
RP4-583P15.16	*ARFRP1*	antisense	protein_coding	2.44	-0.31	HPV16+/HPV16-
RP11-932O9.7	*ARHGAP11B*	antisense	protein_coding	1.13	0.93	HPV16+/HPV16-
ARHGAP5-AS1	*ARHGAP5*	antisense	protein_coding	-1.31	-0.35	tumors/normal
RP11-498C9.3	*ARHGDIA*	antisense	protein_coding	-1.33	-0.58	HPV16+/HPV16-
ASMTL-AS1	*ASMTL*	antisense	protein_coding	1.04	0.64	HPV16+/HPV16-
CTD-2531D15.5	*ASRGL1*	antisense	protein_coding	2.91	1.72	HPV16+/HPV16-
AC092597.3	*ATP10D*	antisense	protein_coding	-1.76	-0.96	HPV16+/HPV16-
ATP1B3-AS1	*ATP1B3*	antisense	protein_coding	3.91	0.97	tumors/normal
RP11-517C16.2	*ATP2C2*	antisense	protein_coding	-1.38	-1.71	HPV16+/HPV16-
BBOX1-AS1	*BBOX1*	antisense	protein_coding	2.86	-1.06	tumors/normal
RP11-284F21.10	*BCAN*	antisense	protein_coding	2.13	2.53	tumors/normal
RP11-284F21.7	*BCAN*	antisense	protein_coding	2.59	2.53	tumors/normal
TBX2-AS1	*BCAS3*	antisense	protein_coding	-1.34	0.49	HPV16+/HPV16-
RP11-662I13.2	*BCAT1*	antisense	protein_coding	1.46	1.09	tumors/normal

RP11-521C20.3	*BMF*	antisense	protein_coding	1.52	0.72	HPV16+/HPV16-
PPIEL	*BMP8A*	antisense	protein_coding	1.60	3.39	tumors/normal
RP11-867G23.12	*BRMS1*	antisense	protein_coding	-1.32	-0.69	HPV16+/HPV16-
RP11-317N8.5	*BRMS1L*	antisense	protein_coding	3.31	0.13	tumors/normal
RP1-179N16.6	*BRPF3*	antisense	protein_coding	1.17	0.35	HPV16+/HPV16-
AC009005.2	*BSG*	antisense	protein_coding	1.97	-0.13	HPV16+/HPV16-
BZRAP1-AS1	*BZRAP1*	antisense	protein_coding	1.60	1.93	HPV16+/HPV16-
RP11-966I7.3	*C14orf23*	antisense	protein_coding	1.07	0.22	HPV16+/HPV16-
RP11-857B24.5	*C14orf28*	antisense	protein_coding	2.93	0.00	HPV16+/HPV16-
CTC-429P9.1	*C19orf44*	antisense	protein_coding	1.01	0.43	HPV16+/HPV16-
CTC-453G23.8	*C19orf68*	antisense	protein_coding	1.27	0.74	HPV16+/HPV16-
RP11-159F24.6	*C5orf34*	antisense	protein_coding	2.68	1.60	tumors/normal
RP11-159F24.5	*C5orf34*	antisense	protein_coding	4.89	1.60	tumors/normal
AC116366.6	*C5orf56*	antisense	protein_coding	1.33	1.26	HPV16+/HPV16-
AC007319.1	*CALCRL*	antisense	protein_coding	2.47	-0.22	HPV16+/HPV16-
CTC-499J9.1	*CAMK4*	antisense	protein_coding	-2.29	1.19	HPV16+/HPV16-
RP5-1050D4.2	*CAMTA2*	antisense	protein_coding	-1.22	-0.15	tumors/normal
CTC-453G23.8	*CARD8*	antisense	protein_coding	1.27	0.52	HPV16+/HPV16-
RP4-734G22.3	*CASZ1*	antisense	protein_coding	-1.24	-1.07	tumors/normal
RP4-622L5.7	*CCDC28B*	antisense	protein_coding	1.14	1.13	HPV16+/HPV16-
RP11-473M20.5	*CCDC64B*	antisense	protein_coding	-1.69	-2.01	tumors/normal
AL022341.3	*CCDC78*	antisense	protein_coding	-1.33	1.42	HPV16+/HPV16-
RP11-727A23.10	*CCDC90B*	antisense	protein_coding	-2.19	0.07	HPV16+/HPV16-
CTB-91J4.1	*CCL3L3*	antisense	protein_coding	1.05	0.59	HPV16+/HPV16-
CTB-91J4.1	*CCL4L1*	antisense	protein_coding	1.05	0.42	HPV16+/HPV16-

RP1-199J3.7	*CCNC*	antisense	protein_coding	-1.06	-0.01	tumors/normal
RP11-347C18.5	*CCNE2*	antisense	protein_coding	2.20	1.49	HPV16+/HPV16-
RP11-715J22.3	*CCNF*	antisense	protein_coding	1.59	0.99	HPV16+/HPV16-
AP006621.8	*CD151*	antisense	protein_coding	-1.33	0.61	tumors/normal
RP1-68D18.4	*CD44*	antisense	protein_coding	-1.27	-1.28	HPV16+/HPV16-
AC006369.2	*CDC42EP3*	antisense	protein_coding	1.52	-0.88	HPV16+/HPV16-
RP11-615I2.2	*CDH3*	antisense	protein_coding	-1.37	-1.24	HPV16+/HPV16-
RP11-615I2.2	*CDH3*	antisense	protein_coding	-1.37	-0.88	HPV16+/HPV16-
CDIPT-AS1	*CDIPT*	antisense	protein_coding	2.39	-0.55	HPV16+/HPV16-
AC002454.1	*CDK6*	antisense	protein_coding	-4.31	-2.13	HPV16+/HPV16-
CDKN2B-AS	*CDKN2A*	antisense	protein_coding	2.02	2.44	tumors/normal
CDKN2B-AS	*CDKN2B*	antisense	protein_coding	2.02	-0.44	tumors/normal
RP11-443B20.1	*CENPO*	antisense	protein_coding	1.84	1.02	tumors/normal
RP3-477O4.14	*CEP250*	antisense	protein_coding	1.03	0.55	HPV16+/HPV16-
CTD-2589H19.6	*CEP72*	antisense	protein_coding	1.41	1.22	tumors/normal
RP11-168G16.2	*CERS3*	antisense	protein_coding	-1.44	-2.20	HPV16+/HPV16-
CTB-50L17.5	*CHAF1A*	antisense	protein_coding	1.49	1.51	HPV16+/HPV16-
AC005162.5	*CHN2*	antisense	protein_coding	-3.50	-0.07	tumors/normal
CTB-113D17.1	*CHN2*	antisense	protein_coding	-3.46	-0.07	tumors/normal
RP4-651E10.4	*CLCA4*	antisense	protein_coding	-4.58	-4.76	tumors/normal
LA16c-390E6.4	*CLCN7*	antisense	protein_coding	2.28	0.80	tumors/normal
AC002116.7	*CLIP3*	antisense	protein_coding	1.49	0.50	tumors/normal
RP11-273B20.1	*CLSTN3*	antisense	protein_coding	2.25	0.83	tumors/normal
RP11-74E22.3	*CLUH*	antisense	protein_coding	1.80	0.11	HPV16+/HPV16-
RP11-829H16.3	*COCH*	antisense	protein_coding	1.83	2.57	HPV16+/HPV16-

RP3-486I3.5	*COL10A1*	antisense	protein_coding	1.22	-1.37	HPV16+/HPV16-
COL4A2-AS1	*COL4A1*	antisense	protein_coding	-1.40	-1.33	HPV16+/HPV16-
COL4A2-AS1	*COL4A2*	antisense	protein_coding	-1.40	-1.20	HPV16+/HPV16-
COLCA1	*COLCA2*	antisense	protein_coding	1.19	2.35	HPV16+/HPV16-
RP11-968A15.2	*COPZ1*	antisense	protein_coding	3.74	-0.04	tumors/normal
RP11-455F5.5	*CORO1A*	antisense	protein_coding	1.76	1.25	HPV16+/HPV16-
RP11-452L6.8	*COX6A2*	antisense	protein_coding	-5.38	-5.31	tumors/normal
RP11-665G4.1	*CPEB2*	antisense	protein_coding	-1.72	-0.99	HPV16+/HPV16-
AC005162.5	*CPVL*	antisense	protein_coding	-3.50	-0.49	tumors/normal
CTB-113D17.1	*CPVL*	antisense	protein_coding	-3.46	-0.49	tumors/normal
CTD-2396E7.11	*CRB3*	antisense	protein_coding	1.11	0.82	HPV16+/HPV16-
RP11-97C16.1	*CRBN*	antisense	protein_coding	1.52	0.95	HPV16+/HPV16-
AC007879.5	*CREB1*	antisense	protein_coding	-1.34	0.16	HPV16+/HPV16-
RP11-78I14.1	*CRIM1*	antisense	protein_coding	-1.70	-1.52	HPV16+/HPV16-
CTD-2026K11.1	*CSPG4*	antisense	protein_coding	-2.79	-2.71	HPV16+/HPV16-
RP11-134G8.7	*CSRP1*	antisense	protein_coding	1.00	0.05	HPV16+/HPV16-
AC009095.4	*CTCF*	antisense	protein_coding	1.32	0.13	HPV16+/HPV16-
RP11-235E17.6	*CTNS*	antisense	protein_coding	1.43	0.51	tumors/normal
CTA-797E19.2	*CTTN*	antisense	protein_coding	-1.54	-1.60	HPV16+/HPV16-
AP000487.6	*CTTN*	antisense	protein_coding	-1.41	-1.60	HPV16+/HPV16-
AP000487.5	*CTTN*	antisense	protein_coding	-1.05	-1.60	HPV16+/HPV16-
RP5-1142A6.2	*CTU2*	antisense	protein_coding	-1.40	-0.66	HPV16+/HPV16-
RP5-1142A6.9	*CTU2*	antisense	protein_coding	-1.06	-0.66	HPV16+/HPV16-
RP11-144G7.2	*CUL5*	antisense	protein_coding	-1.25	-0.89	tumors/normal
RP3-330M21.5	*CUL9*	antisense	protein_coding	1.71	1.36	HPV16+/HPV16-

RP11-316M21.6	*CWF19L1*	antisense	protein_coding	1.03	0.57	tumors/normal
CTC-321K16.1	*CXCL14*	antisense	protein_coding	-2.75	-3.85	HPV16+/HPV16-
LINC00894	*CXorf40B*	antisense	protein_coding	1.37	0.37	tumors/normal
AC114730.7	*D2HGDH*	antisense	protein_coding	1.13	0.28	HPV16+/HPV16-
RP11-15B17.1	*DDIT4L*	antisense	protein_coding	1.08	-1.36	HPV16+/HPV16-
DDX11-AS1	*DDX11*	antisense	protein_coding	2.39	1.66	tumors/normal
RP11-96D1.5	*DDX28*	antisense	protein_coding	1.15	-0.53	HPV16+/HPV16-
AC005932.1	*DDX49*	antisense	protein_coding	-1.55	0.09	HPV16+/HPV16-
CTD-2396E7.11	*DENND1C*	antisense	protein_coding	1.11	1.11	HPV16+/HPV16-
RP11-755B10.2	*DENND6A*	antisense	protein_coding	1.37	0.34	HPV16+/HPV16-
RP4-694A7.2	*DEPDC1*	antisense	protein_coding	-2.70	0.18	HPV16+/HPV16-
AC053503.6	*DES*	antisense	protein_coding	-5.74	-5.20	tumors/normal
RP11-506H20.1	*DHX29*	antisense	protein_coding	1.36	-0.33	HPV16+/HPV16-
CTB-161M19.4	*DMXL1*	antisense	protein_coding	1.33	0.51	HPV16+/HPV16-
RP11-278C7.4	*DNM1L*	antisense	protein_coding	1.09	-0.36	HPV16+/HPV16-
RP11-96D1.5	*DPEP2*	antisense	protein_coding	1.15	0.29	HPV16+/HPV16-
AC104699.1	*DTNB*	antisense	protein_coding	1.90	0.51	HPV16+/HPV16-
CTD-2269F5.1	*EDIL3*	antisense	protein_coding	-2.26	-2.28	HPV16+/HPV16-
AP006621.8	*EFCAB4A*	antisense	protein_coding	-1.33	-1.59	tumors/normal
CTD-2263F21.1	*EGFLAM*	antisense	protein_coding	-3.29	-0.59	HPV16+/HPV16-
RP11-875O11.3	*EGR3*	antisense	protein_coding	3.12	-1.71	tumors/normal
EHMT2-AS1	*EHMT2*	antisense	protein_coding	1.52	0.44	HPV16+/HPV16-
AC019349.5	*EIF1*	antisense	protein_coding	9.55	7.20	HPV16+/HPV16-
AC074117.10	*EIF2B4*	antisense	protein_coding	1.38	1.44	HPV16+/HPV16-
GTF3C2-AS1	*EIF2B4*	antisense	protein_coding	1.81	1.44	HPV16+/HPV16-

RP1-117B12.4	*EME1*	antisense	protein_coding	-1.09	0.69	HPV16+/HPV16-
CTC-459M5.2	*EPB41L4A*	antisense	protein_coding	1.56	1.26	HPV16+/HPV16-
RP11-526F3.1	*EPB41L4A*	antisense	protein_coding	4.89	1.26	HPV16+/HPV16-
AC012363.4	*EPB41L5*	antisense	protein_coding	-1.91	-0.32	HPV16+/HPV16-
RP11-276H7.2	*EPHA2*	antisense	protein_coding	-1.57	-1.02	tumors/normal
CTA-984G1.5	*EWSR1*	antisense	protein_coding	1.21	0.20	tumors/normal
RP11-656D10.6	*EXO5*	antisense	protein_coding	1.41	0.86	HPV16+/HPV16-
RP11-656D10.5	*EXO5*	antisense	protein_coding	1.72	0.86	HPV16+/HPV16-
RP11-363E6.3	*FABP5*	antisense	protein_coding	-1.78	-1.87	HPV16+/HPV16-
RP11-805J14.5	*FADD*	antisense	protein_coding	-1.28	-1.22	HPV16+/HPV16-
RP11-475O23.3	*FAM107A*	antisense	protein_coding	-1.49	-4.69	tumors/normal
AL022341.3	*FAM173A*	antisense	protein_coding	-1.33	0.36	HPV16+/HPV16-
AL022341.3	*FAM195A*	antisense	protein_coding	-1.33	0.33	HPV16+/HPV16-
AC006026.13	*FAM221A*	antisense	protein_coding	2.46	0.95	HPV16+/HPV16-
RP11-475O23.3	*FAM3D*	antisense	protein_coding	-1.49	-6.27	tumors/normal
RP11-30K9.5	*FAM63B*	antisense	protein_coding	2.27	-0.18	HPV16+/HPV16-
RP11-539E17.5	*FAM83A*	antisense	protein_coding	-1.98	-1.99	HPV16+/HPV16-
FAM83A-AS1	*FAM83A*	antisense	protein_coding	-1.90	-1.99	HPV16+/HPV16-
VPS9D1-AS1	*FANCA*	antisense	protein_coding	-1.68	0.85	HPV16+/HPV16-
RP1-232P20.1	*FARS2*	antisense	protein_coding	-4.26	0.35	tumors/normal
PCAT7	*FBP2*	antisense	protein_coding	1.22	-1.65	tumors/normal
AL022341.3	*FBXL16*	antisense	protein_coding	-1.33	0.42	HPV16+/HPV16-
FEZF1-AS1	*FEZF1*	antisense	protein_coding	2.32	2.77	HPV16+/HPV16-
AC005592.2	*FGF1*	antisense	protein_coding	-1.75	0.42	HPV16+/HPV16-
AC073635.5	*FGL2*	antisense	protein_coding	1.56	0.40	HPV16+/HPV16-

AC019349.5	*FKBP10*	antisense	protein_coding	9.55	4.19	HPV16+/HPV16-
AC007285.6	*FKBP14*	antisense	protein_coding	-1.38	-0.70	HPV16+/HPV16-
AC005387.3	*FKBP8*	antisense	protein_coding	2.41	-0.13	tumors/normal
AC005387.2	*FKBP8*	antisense	protein_coding	2.49	-0.13	tumors/normal
LA16c-321D4.2	*FLYWCH1*	antisense	protein_coding	3.38	0.59	tumors/normal
RP1-127D3.4	*FMO2*	antisense	protein_coding	-5.67	-5.17	tumors/normal
CTD-2058B24.2	*FOXA1*	antisense	protein_coding	-2.79	3.71	HPV16+/HPV16-
FOXD3-AS1	*FOXD3*	antisense	protein_coding	2.98	3.06	HPV16+/HPV16-
RP11-399E6.4	*FOXO6*	antisense	protein_coding	1.52	1.85	HPV16+/HPV16-
FOXP4-AS1	*FOXP4*	antisense	protein_coding	1.94	0.20	HPV16+/HPV16-
AC006942.4	*FUZ*	antisense	protein_coding	1.00	1.12	HPV16+/HPV16-
AC113189.5	*FXR2*	antisense	protein_coding	2.13	0.09	HPV16+/HPV16-
RP11-1103G16.1	*G2E3*	antisense	protein_coding	3.32	0.27	tumors/normal
RP11-92C4.3	*GALNT12*	antisense	protein_coding	-2.23	-2.11	tumors/normal
RP11-475N22.4	*GATA2*	antisense	protein_coding	-1.78	-1.09	HPV16+/HPV16-
RP4-620F22.2	*GBP5*	antisense	protein_coding	3.24	2.75	tumors/normal
GFOD1-AS1	*GFOD1*	antisense	protein_coding	-1.33	-0.90	HPV16+/HPV16-
RP11-360L9.4	*GINS4*	antisense	protein_coding	1.16	1.09	HPV16+/HPV16-
RP11-360L9.7	*GINS4*	antisense	protein_coding	1.61	1.36	tumors/normal
RP11-837J7.4	*GIT2*	antisense	protein_coding	1.00	0.31	HPV16+/HPV16-
SNHG10	*GLRX5*	antisense	protein_coding	1.02	-0.19	tumors/normal
AC073957.15	*GPER1*	antisense	protein_coding	-2.30	0.21	HPV16+/HPV16-
AC073957.15	*GPR146*	antisense	protein_coding	-2.30	0.10	HPV16+/HPV16-
RP11-517I3.1	*GRAMD3*	antisense	protein_coding	1.18	0.25	HPV16+/HPV16-
KB-1562D12.1	*GRHL2*	antisense	protein_coding	-2.04	0.29	tumors/normal

RP11-10N16.3	*GRHL3*	antisense	protein_coding	1.66	0.71	HPV16+/HPV16-
AC073635.5	*GSAP*	antisense	protein_coding	1.56	0.65	HPV16+/HPV16-
CTD-3195I5.4	*GSG2*	antisense	protein_coding	1.72	0.56	HPV16+/HPV16-
AC074117.10	*GTF3C2*	antisense	protein_coding	1.38	1.91	HPV16+/HPV16-
GTF3C2-AS1	*GTF3C2*	antisense	protein_coding	1.81	1.91	HPV16+/HPV16-
RP3-508I15.21	*GTPBP1*	antisense	protein_coding	1.12	0.05	tumors/normal
RP3-508I15.22	*GTPBP1*	antisense	protein_coding	1.26	0.25	HPV16+/HPV16-
RP11-695J4.2	*GUCY2C*	antisense	protein_coding	-2.00	-1.82	tumors/normal
RP11-104E19.1	*GZMB*	antisense	protein_coding	-1.56	1.07	HPV16+/HPV16-
RP11-104E19.1	*GZMH*	antisense	protein_coding	-1.56	2.34	HPV16+/HPV16-
RP11-15B17.1	*H2AFZ*	antisense	protein_coding	1.08	1.07	HPV16+/HPV16-
RP11-396C23.4	*H3F3A*	antisense	protein_coding	1.82	0.96	HPV16+/HPV16-
AL022341.3	*HAGHL*	antisense	protein_coding	-1.33	0.18	HPV16+/HPV16-
AC019349.5	*HAP1*	antisense	protein_coding	9.55	3.95	HPV16+/HPV16-
RP11-473M20.5	*HCFC1R1*	antisense	protein_coding	-1.69	0.08	tumors/normal
RP3-399L15.3	*HDAC2*	antisense	protein_coding	1.05	0.29	HPV16+/HPV16-
RP11-968A15.2	*HNRNPA1*	antisense	protein_coding	3.74	0.09	tumors/normal
HOXA-AS2	*HOXA5*	antisense	protein_coding	1.17	0.86	HPV16+/HPV16-
HOXA-AS2	*HOXA7*	antisense	protein_coding	1.17	0.36	HPV16+/HPV16-
HOXB-AS4	*HOXB7*	antisense	protein_coding	5.19	0.27	tumors/normal
HOXC13-AS	*HOXC13*	antisense	protein_coding	-4.30	-4.17	HPV16+/HPV16-
RP11-510J16.5	*HSD17B2*	antisense	protein_coding	-2.00	-1.60	HPV16+/HPV16-
AC006042.6	*ICA1*	antisense	protein_coding	2.68	2.13	HPV16+/HPV16-
RP1-120G22.11	*ICMT*	antisense	protein_coding	1.63	-0.31	HPV16+/HPV16-
RP11-288L9.4	*IFI6*	antisense	protein_coding	3.74	3.22	tumors/normal

RP11-149I23.3	*IFIT1*	antisense	protein_coding	-2.11	-1.93	HPV16+/HPV16-
RP11-149I23.3	*IFIT2*	antisense	protein_coding	-2.11	-0.92	HPV16+/HPV16-
RP11-149I23.3	*IFIT3*	antisense	protein_coding	-2.11	-1.03	HPV16+/HPV16-
RP11-6O2.4	*IGF1R*	antisense	protein_coding	-3.15	0.61	tumors/normal
AC007563.5	*IGFBP5*	antisense	protein_coding	-2.21	-2.04	tumors/normal
IGFBP7-AS1	*IGFBP7*	antisense	protein_coding	1.85	1.78	tumors/normal
CTD-2031P19.5	*IL6ST*	antisense	protein_coding	-2.13	-1.38	tumors/normal
RP11-347C18.5	*INTS8*	antisense	protein_coding	2.20	0.10	HPV16+/HPV16-
RP4-622L5.7	*IQCC*	antisense	protein_coding	1.14	0.63	HPV16+/HPV16-
CTD-2194D22.3	*IRX4*	antisense	protein_coding	-4.14	-3.49	HPV16+/HPV16-
ISPD-AS1	*ISPD*	antisense	protein_coding	-3.59	-1.36	HPV16+/HPV16-
CTD-2288O8.1	*ITGA2*	antisense	protein_coding	2.90	0.55	tumors/normal
AC078883.4	*ITGA6*	antisense	protein_coding	-1.04	-1.26	HPV16+/HPV16-
AC093818.1	*ITGA6*	antisense	protein_coding	1.04	1.40	tumors/normal
CTD-3195I5.4	*ITGAE*	antisense	protein_coding	1.72	0.59	HPV16+/HPV16-
ITGB2-AS1	*ITGB2*	antisense	protein_coding	1.45	0.62	HPV16+/HPV16-
AC019349.5	*JUP*	antisense	protein_coding	9.55	6.92	HPV16+/HPV16-
RP11-677I18.3	*KBTBD3*	antisense	protein_coding	-1.11	-0.16	HPV16+/HPV16-
RP11-305K5.1	*KCNAB1*	antisense	protein_coding	1.21	0.04	HPV16+/HPV16-
RP11-542C16.1	*KCTD11*	antisense	protein_coding	-1.25	-1.21	HPV16+/HPV16-
RP11-635N19.1	*KDSR*	antisense	protein_coding	1.49	-0.11	HPV16+/HPV16-
RP11-843P14.1	*KIAA1191*	antisense	protein_coding	1.12	0.21	HPV16+/HPV16-
CTA-221G9.11	*KIAA1671*	antisense	protein_coding	-1.85	-0.50	tumors/normal
CTD-2647L4.1	*KIF13B*	antisense	protein_coding	-3.35	-1.68	tumors/normal
AC109333.10	*KIF1C*	antisense	protein_coding	-1.20	-1.67	tumors/normal

AC009133.21	*KIF22*	antisense	protein_coding	1.00	0.58	HPV16+/HPV16-
RP11-253M7.1	*KIF23*	antisense	protein_coding	-1.62	0.56	HPV16+/HPV16-
CTD-2600O9.2	*KIFC3*	antisense	protein_coding	-1.38	-1.18	HPV16+/HPV16-
RP11-254F7.3	*KLF11*	antisense	protein_coding	1.09	1.11	HPV16+/HPV16-
CTC-518B2.12	*KLK10*	antisense	protein_coding	-4.86	-3.45	HPV16+/HPV16-
CTC-518B2.9	*KLK12*	antisense	protein_coding	-2.65	-3.20	HPV16+/HPV16-
CTB-147C22.8	*KLK5*	antisense	protein_coding	-5.39	-6.41	HPV16+/HPV16-
CTB-147C22.9	*KLK5*	antisense	protein_coding	-4.53	-6.41	HPV16+/HPV16-
CTB-147C22.8	*KLK6*	antisense	protein_coding	-5.39	-4.37	HPV16+/HPV16-
CTB-147C22.9	*KLK6*	antisense	protein_coding	-4.53	-4.37	HPV16+/HPV16-
CTB-147C22.8	*KLK7*	antisense	protein_coding	-5.39	-4.84	HPV16+/HPV16-
CTB-147C22.9	*KLK7*	antisense	protein_coding	-4.53	-4.84	HPV16+/HPV16-
RP3-416H24.1	*KRT7*	antisense	protein_coding	1.53	1.13	HPV16+/HPV16-
RP11-845M18.6	*KRT7*	antisense	protein_coding	3.17	1.13	HPV16+/HPV16-
RP3-416H24.1	*KRT86*	antisense	protein_coding	1.53	2.31	HPV16+/HPV16-
RP11-845M18.6	*KRT86*	antisense	protein_coding	3.17	2.31	HPV16+/HPV16-
KTN1-AS1	*KTN1*	antisense	protein_coding	-1.17	-0.75	HPV16+/HPV16-
RP11-523H20.3	*LBX2*	antisense	protein_coding	3.48	3.42	tumors/normal
LENG8-AS1	*LENG8*	antisense	protein_coding	1.16	0.28	tumors/normal
LENG8-AS1	*LENG9*	antisense	protein_coding	1.16	-0.47	tumors/normal
CTD-2184D3.5	*LEO1*	antisense	protein_coding	-2.20	-0.12	HPV16+/HPV16-
RP11-56B16.4	*LEO1*	antisense	protein_coding	-1.16	-0.02	tumors/normal
LEPREL1-AS1	*LEPREL1*	antisense	protein_coding	1.24	2.07	tumors/normal
AC019349.5	*LEPREL4*	antisense	protein_coding	9.55	4.99	HPV16+/HPV16-
CTC-453G23.5	*LIG1*	antisense	protein_coding	1.62	1.64	HPV16+/HPV16-

RP11-149I23.3	*LIPA*	antisense	protein_coding	-2.11	-0.62	HPV16+/HPV16-
AC005534.8	*LMBR1*	antisense	protein_coding	1.20	-0.49	HPV16+/HPV16-
LMCD1-AS1	*LMCD1*	antisense	protein_coding	1.55	-1.58	tumors/normal
RP1-117B12.4	*LRRC59*	antisense	protein_coding	-1.09	-0.73	HPV16+/HPV16-
CTD-3193O13.12	*LRRC8E*	antisense	protein_coding	-1.07	-0.37	HPV16+/HPV16-
RP11-66B24.4	*LRRK1*	antisense	protein_coding	-1.44	2.21	tumors/normal
AC002128.5	*LSR*	antisense	protein_coding	1.38	0.28	HPV16+/HPV16-
CTD-2292P10.4	*LY6K*	antisense	protein_coding	2.41	3.08	tumors/normal
RP11-706C16.7	*LYNX1*	antisense	protein_coding	-2.27	-1.94	HPV16+/HPV16-
AC103563.8	*MAL*	antisense	protein_coding	-4.91	-6.35	tumors/normal
AC103563.9	*MAL*	antisense	protein_coding	-4.83	-6.35	tumors/normal
RP11-109P14.9	*MANEAL*	antisense	protein_coding	1.73	1.37	HPV16+/HPV16-
CTB-186G2.1	*MAP4K1*	antisense	protein_coding	2.27	1.24	HPV16+/HPV16-
CTD-2184D3.5	*MAPK6*	antisense	protein_coding	-2.20	-0.79	HPV16+/HPV16-
RP11-56B16.4	*MAPK6*	antisense	protein_coding	-1.16	0.35	tumors/normal
LA16c-361A3.3	*MAPK8IP3*	antisense	protein_coding	2.17	0.47	tumors/normal
MAST4-AS1	*MAST4*	antisense	protein_coding	1.50	-0.21	HPV16+/HPV16-
MCCC1-AS1	*MCCC1*	antisense	protein_coding	1.47	0.90	HPV16+/HPV16-
MCF2L-AS1	*MCF2L*	antisense	protein_coding	2.36	1.09	HPV16+/HPV16-
AL022341.3	*METRN*	antisense	protein_coding	-1.33	-0.95	HPV16+/HPV16-
AC007879.5	*METTL21A*	antisense	protein_coding	-1.34	-0.21	HPV16+/HPV16-
MFI2-AS1	*MFI2*	antisense	protein_coding	1.64	1.97	tumors/normal
RP11-345P4.9	*MIB2*	antisense	protein_coding	-1.96	0.38	tumors/normal
RP11-265D17.2	*MICAL2*	antisense	protein_coding	-1.72	-1.07	HPV16+/HPV16-
CTB-58E17.9	*MLLT6*	antisense	protein_coding	1.19	0.87	HPV16+/HPV16-

RP11-465L10.10	*MMP9*	antisense	protein_coding	3.70	4.00	tumors/normal
RP11-426L16.3	*MOV10*	antisense	protein_coding	1.17	0.36	HPV16+/HPV16-
AC113189.5	*MPDU1*	antisense	protein_coding	2.13	0.29	HPV16+/HPV16-
RP5-1120P11.1	*MRPL14*	antisense	protein_coding	1.05	-0.27	HPV16+/HPV16-
AL022341.3	*MSLN*	antisense	protein_coding	-1.33	0.70	HPV16+/HPV16-
RP11-395B7.4	*MUC12*	antisense	protein_coding	-1.60	-1.89	HPV16+/HPV16-
AC118754.4	*MYBBP1A*	antisense	protein_coding	-2.95	0.01	tumors/normal
RP11-755O11.2	*MYBPC1*	antisense	protein_coding	-5.97	-6.12	tumors/normal
AF001548.6	*MYH11*	antisense	protein_coding	-2.78	-1.43	tumors/normal
CTB-191K22.5	*MYH14*	antisense	protein_coding	1.96	1.79	HPV16+/HPV16-
CTD-2201G16.1	*MYH7*	antisense	protein_coding	-7.04	-5.78	tumors/normal
RP11-466A19.1	*MYO1D*	antisense	protein_coding	1.06	0.21	tumors/normal
CTD-2524L6.3	*MYO9A*	antisense	protein_coding	-1.32	-0.45	HPV16+/HPV16-
RP1-267L14.3	*NAA25*	antisense	protein_coding	1.19	0.20	tumors/normal
AC006460.2	*NAB1*	antisense	protein_coding	-1.12	-0.44	HPV16+/HPV16-
RP11-22N19.2	*NAMPT*	antisense	protein_coding	-1.48	-0.86	HPV16+/HPV16-
RP4-694B14.8	*NANP*	antisense	protein_coding	1.59	0.12	HPV16+/HPV16-
AL022341.3	*NARFL*	antisense	protein_coding	-1.33	0.32	HPV16+/HPV16-
NAV2-AS2	*NAV2*	antisense	protein_coding	-1.89	-0.35	tumors/normal
RP11-347C18.5	*NDUFAF6*	antisense	protein_coding	2.20	0.80	HPV16+/HPV16-
CTB-79E8.2	*NEURL1B*	antisense	protein_coding	1.98	1.82	HPV16+/HPV16-
RP11-10N16.3	*NIPAL3*	antisense	protein_coding	1.66	0.23	HPV16+/HPV16-
RP4-613B23.1	*NKTR*	antisense	protein_coding	-2.07	-0.12	tumors/normal
LA16c-361A3.3	*NME3*	antisense	protein_coding	2.17	-0.10	tumors/normal
NNT-AS1	*NNT*	antisense	protein_coding	2.39	-1.69	tumors/normal

IGFBP7-AS1	*NOA1*	antisense	protein_coding	1.85	-0.31	tumors/normal
CTD-2033A16.3	*NOB1*	antisense	protein_coding	-2.37	-0.63	HPV16+/HPV16-
STARD4-AS1	*NREP*	antisense	protein_coding	1.81	1.28	tumors/normal
AF127577.10	*NRIP1*	antisense	protein_coding	-1.09	-1.30	HPV16+/HPV16-
RP11-342D11.2	*NRP1*	antisense	protein_coding	-1.42	-0.96	HPV16+/HPV16-
RP3-486I3.5	*NT5DC1*	antisense	protein_coding	1.22	0.41	HPV16+/HPV16-
RP11-256I23.2	*OBSL1*	antisense	protein_coding	-3.18	-0.59	tumors/normal
CTD-3065J16.6	*OPLAH*	antisense	protein_coding	2.25	-0.63	tumors/normal
RP11-203M5.7	*OSGEP*	antisense	protein_coding	1.26	0.65	HPV16+/HPV16-
RP11-415F23.2	*OXNAD1*	antisense	protein_coding	1.56	0.91	HPV16+/HPV16-
RP11-415F23.4	*OXNAD1*	antisense	protein_coding	1.74	0.91	HPV16+/HPV16-
RP11-415F23.3	*OXNAD1*	antisense	protein_coding	1.85	0.91	HPV16+/HPV16-
RP11-964E11.2	*PAX9*	antisense	protein_coding	3.74	2.18	HPV16+/HPV16-
RP11-1191J2.5	*PDE6B*	antisense	protein_coding	3.01	1.61	HPV16+/HPV16-
RP11-802O23.3	*PDHB*	antisense	protein_coding	-1.67	-1.40	tumors/normal
AC078883.4	*PDK1*	antisense	protein_coding	-1.04	-0.02	HPV16+/HPV16-
AC093818.1	*PDK1*	antisense	protein_coding	1.04	1.02	tumors/normal
CTD-3126B10.1	*PDPK1*	antisense	protein_coding	-1.09	-0.16	tumors/normal
RP11-129M16.4	*PDZD8*	antisense	protein_coding	-1.21	-0.87	HPV16+/HPV16-
PDZRN3-AS1	*PDZRN3*	antisense	protein_coding	-4.12	-2.29	tumors/normal
RP11-136L23.2	*PEBP1*	antisense	protein_coding	1.14	0.16	HPV16+/HPV16-
RP11-875O11.3	*PEBP4*	antisense	protein_coding	3.12	-7.36	tumors/normal
RP3-467L1.4	*PER3*	antisense	protein_coding	1.88	1.66	HPV16+/HPV16-
RP11-6O2.4	*PGPEP1L*	antisense	protein_coding	-3.15	-1.62	tumors/normal
RP11-415J8.3	*PHC2*	antisense	protein_coding	1.60	-0.06	tumors/normal

RP11-290L1.3	*PHLDA1*	antisense	protein_coding	-1.23	-1.27	HPV16+/HPV16-
RP5-1142A6.2	*PIEZO1*	antisense	protein_coding	-1.40	-0.83	HPV16+/HPV16-
RP5-1142A6.9	*PIEZO1*	antisense	protein_coding	-1.06	-0.83	HPV16+/HPV16-
L34079.4	*PINLYP*	antisense	protein_coding	1.53	-0.43	HPV16+/HPV16-
CTD-2265M8.2	*PLA2G4C*	antisense	protein_coding	1.16	0.21	HPV16+/HPV16-
RP11-695J4.2	*PLBD1*	antisense	protein_coding	-2.00	-0.73	tumors/normal
RP3-395M20.2	*PLCH2*	antisense	protein_coding	1.26	0.22	HPV16+/HPV16-
RP3-395M20.3	*PLCH2*	antisense	protein_coding	1.32	0.22	HPV16+/HPV16-
RP5-1059L7.1	*PMEPA1*	antisense	protein_coding	-1.90	-1.99	HPV16+/HPV16-
AP006621.8	*PNPLA2*	antisense	protein_coding	-1.33	-1.13	tumors/normal
RP11-334A14.5	*PODN*	antisense	protein_coding	-2.71	-1.56	tumors/normal
IGFBP7-AS1	*POLR2B*	antisense	protein_coding	1.85	-0.19	tumors/normal
AP006621.8	*POLR2L*	antisense	protein_coding	-1.33	-0.30	tumors/normal
CTD-2530N21.4	*POLR3D*	antisense	protein_coding	1.19	-0.01	HPV16+/HPV16-
POU6F2-AS2	*POU6F2*	antisense	protein_coding	-4.90	-1.88	HPV16+/HPV16-
CTA-797E19.2	*PPFIA1*	antisense	protein_coding	-1.54	-0.89	HPV16+/HPV16-
AP000487.6	*PPFIA1*	antisense	protein_coding	-1.41	-0.89	HPV16+/HPV16-
AP000487.5	*PPFIA1*	antisense	protein_coding	-1.05	-0.89	HPV16+/HPV16-
RP11-359P5.1	*PPIC*	antisense	protein_coding	-1.20	-0.48	tumors/normal
PRC1-AS1	*PRC1*	antisense	protein_coding	1.06	0.94	HPV16+/HPV16-
AC106786.1	*PRDM6*	antisense	protein_coding	1.57	1.22	HPV16+/HPV16-
PRKAG2-AS1	*PRKAG2*	antisense	protein_coding	2.05	0.33	HPV16+/HPV16-
RP11-42O4.2	*PROCR*	antisense	protein_coding	-2.03	-2.09	HPV16+/HPV16-
PROSER2-AS1	*PROSER2*	antisense	protein_coding	1.70	0.09	HPV16+/HPV16-
UBE2R2-AS1	*PRSS3*	antisense	protein_coding	-1.21	-2.13	HPV16+/HPV16-

RP11-523H24.3	*PSPC1*	antisense	protein_coding	1.29	0.82	HPV16+/HPV16-
RP11-993B23.3	*PTHLH*	antisense	protein_coding	-4.59	-4.31	HPV16+/HPV16-
RP11-507K2.3	*PTPN21*	antisense	protein_coding	-1.15	-0.80	tumors/normal
RP11-785D18.3	*PTPRB*	antisense	protein_coding	-1.77	-0.23	HPV16+/HPV16-
RP11-443B20.1	*PTRHD1*	antisense	protein_coding	1.84	-0.68	tumors/normal
CTB-129P6.4	*PVRL2*	antisense	protein_coding	-1.37	-0.40	tumors/normal
RP11-218E20.5	*PYGL*	antisense	protein_coding	-2.90	-1.76	HPV16+/HPV16-
RP11-839G9.1	*RAB27B*	antisense	protein_coding	1.81	0.85	HPV16+/HPV16-
CTD-2024P10.1	*RAI14*	antisense	protein_coding	-2.34	-0.53	HPV16+/HPV16-
RP13-225O21.2	*RALGPS1*	antisense	protein_coding	2.38	1.29	HPV16+/HPV16-
RARA-AS1	*RARA*	antisense	protein_coding	-1.66	-0.13	tumors/normal
RP11-780K2.1	*RBM19*	antisense	protein_coding	-1.09	-0.43	HPV16+/HPV16-
RBMS3-AS3	*RBMS3*	antisense	protein_coding	-3.23	-0.80	tumors/normal
RP11-33N14.5	*RFC5*	antisense	protein_coding	3.40	1.03	HPV16+/HPV16-
RP11-415F23.2	*RFTN1*	antisense	protein_coding	1.56	-0.15	HPV16+/HPV16-
RP11-415F23.4	*RFTN1*	antisense	protein_coding	1.74	-0.15	HPV16+/HPV16-
RP11-415F23.3	*RFTN1*	antisense	protein_coding	1.85	-0.15	HPV16+/HPV16-
CTA-984G1.5	*RHBDD3*	antisense	protein_coding	1.21	0.88	tumors/normal
AL022341.3	*RHBDL1*	antisense	protein_coding	-1.33	0.73	HPV16+/HPV16-
RP11-875O11.3	*RHOBTB2*	antisense	protein_coding	3.12	-0.51	tumors/normal
AL022341.3	*RHOT2*	antisense	protein_coding	-1.33	0.15	HPV16+/HPV16-
RP11-867G23.12	*RIN1*	antisense	protein_coding	-1.32	-1.23	HPV16+/HPV16-
RP11-485G7.5	*RMI2*	antisense	protein_coding	1.59	1.73	HPV16+/HPV16-
RP11-848P1.4	*RNF135*	antisense	protein_coding	-1.89	-0.11	HPV16+/HPV16-
RNF157-AS1	*RNF157*	antisense	protein_coding	3.50	0.60	HPV16+/HPV16-

RP1-120G22.11	*RNF207*	antisense	protein_coding	1.63	1.37	HPV16+/HPV16-
AC005534.8	*RNF32*	antisense	protein_coding	1.20	1.38	HPV16+/HPV16-
RP11-758M4.4	*RP11-758M4.1*	antisense	protein_coding	-1.98	-2.56	HPV16+/HPV16-
RP1-120G22.11	*RPL22*	antisense	protein_coding	1.63	0.32	HPV16+/HPV16-
RRM1-AS1	*RRM1*	antisense	protein_coding	-1.57	0.61	HPV16+/HPV16-
RP11-46J23.1	*RUFY3*	antisense	protein_coding	-1.43	-0.33	tumors/normal
BX470102.3	*S100A6*	antisense	protein_coding	2.52	0.18	tumors/normal
AC113189.5	*SAT2*	antisense	protein_coding	2.13	-0.45	HPV16+/HPV16-
SATB2-AS1	*SATB2*	antisense	protein_coding	-1.32	-0.80	HPV16+/HPV16-
TMEM220-AS1	*SCO1*	antisense	protein_coding	-2.56	-0.09	tumors/normal
AC007285.6	*SCRN1*	antisense	protein_coding	-1.38	-0.86	HPV16+/HPV16-
AC098617.1	*SDPR*	antisense	protein_coding	-1.93	-3.54	tumors/normal
RP4-613B23.1	*SEC22C*	antisense	protein_coding	-2.07	-0.12	tumors/normal
RP3-402G11.27	*SELO*	antisense	protein_coding	1.09	0.01	HPV16+/HPV16-
CTB-118N6.3	*SEMA6A*	antisense	protein_coding	1.85	1.44	HPV16+/HPV16-
RP11-127I20.5	*SEPT12*	antisense	protein_coding	1.90	1.21	HPV16+/HPV16-
CTC-492K19.4	*SERTAD3*	antisense	protein_coding	1.01	0.10	HPV16+/HPV16-
RP4-598P13.1	*SGIP1*	antisense	protein_coding	1.07	3.24	tumors/normal
SGOL1-AS1	*SGOL1*	antisense	protein_coding	1.36	0.84	HPV16+/HPV16-
SH3BP5-AS1	*SH3BP5*	antisense	protein_coding	1.21	0.83	HPV16+/HPV16-
RP11-416N2.4	*SH3PXD2A*	antisense	protein_coding	1.64	-0.62	HPV16+/HPV16-
CTD-2616J11.2	*SIGLEC10*	antisense	protein_coding	1.91	0.67	HPV16+/HPV16-
CTD-2616J11.3	*SIGLEC10*	antisense	protein_coding	2.54	0.67	HPV16+/HPV16-
AP000697.6	*SIM2*	antisense	protein_coding	2.67	2.37	HPV16+/HPV16-
RP5-858L17.1	*SIRPA*	antisense	protein_coding	-3.22	-1.60	HPV16+/HPV16-

RP11-190A12.8	*SLAMF8*	antisense	protein_coding	1.38	0.37	HPV16+/HPV16-
RP11-465L10.10	*SLC12A5*	antisense	protein_coding	3.70	0.77	tumors/normal
RP11-97N19.2	*SLC24A3*	antisense	protein_coding	-2.01	-1.56	tumors/normal
RP11-964E11.2	*SLC25A21*	antisense	protein_coding	3.74	0.44	HPV16+/HPV16-
CTD-2396E7.11	*SLC25A23*	antisense	protein_coding	1.11	1.52	HPV16+/HPV16-
RP11-290D2.6	*SLC25A30*	antisense	protein_coding	-1.54	-0.44	tumors/normal
RP4-681L3.2	*SLC26A9*	antisense	protein_coding	2.10	1.65	HPV16+/HPV16-
RP5-1120P11.1	*SLC29A1*	antisense	protein_coding	1.05	-0.75	HPV16+/HPV16-
RP11-519G16.5	*SLC30A4*	antisense	protein_coding	1.19	-0.02	HPV16+/HPV16-
NCK1-AS1	*SLC35G2*	antisense	protein_coding	1.07	0.98	HPV16+/HPV16-
RP11-731C17.2	*SLC35G2*	antisense	protein_coding	1.34	0.98	HPV16+/HPV16-
CTD-2350C19.2	*SLC46A1*	antisense	protein_coding	1.08	0.04	HPV16+/HPV16-
TM4SF19-AS1	*SLC51A*	antisense	protein_coding	-1.90	0.63	HPV16+/HPV16-
AC069257.8	*SLC51A*	antisense	protein_coding	3.26	0.03	tumors/normal
RP4-536B24.2	*SLC7A5*	antisense	protein_coding	-2.89	-2.41	HPV16+/HPV16-
RP11-93B14.10	*SLCO4A1*	antisense	protein_coding	-1.88	-0.03	tumors/normal
RP11-93B14.9	*SLCO4A1*	antisense	protein_coding	-1.88	-0.03	tumors/normal
RP11-93B14.5	*SLCO4A1*	antisense	protein_coding	-1.35	-0.03	tumors/normal
RP11-30K9.5	*SLTM*	antisense	protein_coding	2.27	0.15	HPV16+/HPV16-
RP11-127I20.5	*SMIM22*	antisense	protein_coding	1.90	3.17	HPV16+/HPV16-
RP11-460I13.2	*SMPDL3B*	antisense	protein_coding	3.05	2.70	HPV16+/HPV16-
RP3-412A9.16	*SMTN*	antisense	protein_coding	-1.25	-1.58	HPV16+/HPV16-
MGC32805	*SNCAIP*	antisense	protein_coding	-2.64	0.02	HPV16+/HPV16-
SNRK-AS1	*SNRK*	antisense	protein_coding	-1.28	-0.33	tumors/normal
RP11-359P5.1	*SNX24*	antisense	protein_coding	-1.20	-0.33	tumors/normal

RP11-52J3.2	*SNX9*	antisense	protein_coding	-1.65	-1.35	tumors/normal
RP11-485G7.5	*SOCS1*	antisense	protein_coding	1.59	0.40	HPV16+/HPV16-
AC113189.5	*SOX15*	antisense	protein_coding	2.13	-1.30	HPV16+/HPV16-
RP11-6N17.3	*SP2*	antisense	protein_coding	-1.18	-0.54	tumors/normal
CTB-113P19.1	*SPARC*	antisense	protein_coding	-1.85	-1.32	HPV16+/HPV16-
RP11-507K2.3	*SPATA7*	antisense	protein_coding	-1.15	-0.21	tumors/normal
FLJ37453	*SPEN*	antisense	protein_coding	1.04	0.47	HPV16+/HPV16-
AC118754.4	*SPNS2*	antisense	protein_coding	-2.95	-2.50	tumors/normal
RP11-21L19.1	*SPON1*	antisense	protein_coding	-2.18	-1.98	HPV16+/HPV16-
RP11-20I20.4	*SPON2*	antisense	protein_coding	-1.18	-1.62	HPV16+/HPV16-
AC005592.2	*SPRY4*	antisense	protein_coding	-1.75	-1.45	HPV16+/HPV16-
RP11-196H14.3	*SRGAP1*	antisense	protein_coding	-1.95	-0.95	HPV16+/HPV16-
RP11-196H14.2	*SRGAP1*	antisense	protein_coding	-1.60	-0.95	HPV16+/HPV16-
RP4-613B23.1	*SS18L2*	antisense	protein_coding	-2.07	-0.11	tumors/normal
AC007292.7	*STAP2*	antisense	protein_coding	2.37	0.51	HPV16+/HPV16-
STARD4-AS1	*STARD4*	antisense	protein_coding	1.81	1.06	tumors/normal
RP11-10N16.3	*STPG1*	antisense	protein_coding	1.66	-0.34	HPV16+/HPV16-
RP11-829H16.3	*STRN3*	antisense	protein_coding	1.83	-0.64	HPV16+/HPV16-
AL022341.3	*STUB1*	antisense	protein_coding	-1.33	-0.17	HPV16+/HPV16-
AF196970.3	*SUV39H1*	antisense	protein_coding	1.25	1.18	HPV16+/HPV16-
RP11-6O2.4	*SYNM*	antisense	protein_coding	-3.15	-2.73	tumors/normal
RP11-793H13.11	*TARBP2*	antisense	protein_coding	-1.79	0.31	tumors/normal
RP11-347C12.11	*TBC1D10B*	antisense	protein_coding	1.37	0.28	tumors/normal
RP11-353N14.7	*TBC1D16*	antisense	protein_coding	-2.04	-1.60	HPV16+/HPV16-
TBX2-AS1	*TBX2*	antisense	protein_coding	-1.34	-1.15	HPV16+/HPV16-

RP13-152O15.5	*TCEA2*	antisense	protein_coding	1.99	0.25	HPV16+/HPV16-
RP11-837J7.4	*TCHP*	antisense	protein_coding	1.00	0.59	HPV16+/HPV16-
AC091177.1	*TEFM*	antisense	protein_coding	-1.74	0.64	HPV16+/HPV16-
MGC45800	*TENM3*	antisense	protein_coding	-1.15	-1.76	HPV16+/HPV16-
AC007319.1	*TFPI*	antisense	protein_coding	2.47	0.07	HPV16+/HPV16-
AC010883.5	*THADA*	antisense	protein_coding	1.22	-0.10	HPV16+/HPV16-
AC002116.7	*THAP8*	antisense	protein_coding	1.49	-0.06	tumors/normal
CTD-2033D15.3	*THBS1*	antisense	protein_coding	-1.86	-1.68	HPV16+/HPV16-
CTD-2033D15.1	*THBS1*	antisense	protein_coding	-1.83	-1.68	HPV16+/HPV16-
XXyac-YX65C7_A.2	*THBS2*	antisense	protein_coding	-1.47	-2.40	HPV16+/HPV16-
AL450992.2	*THEM5*	antisense	protein_coding	2.29	0.97	HPV16+/HPV16-
RP11-473M20.5	*THOC6*	antisense	protein_coding	-1.69	-0.03	tumors/normal
RP11-1123I8.1	*THSD4*	antisense	protein_coding	-6.25	-1.98	tumors/normal
USP2-AS1	*THY1*	antisense	protein_coding	-1.20	-0.77	HPV16+/HPV16-
AP000251.3	*TIAM1*	antisense	protein_coding	2.80	0.12	HPV16+/HPV16-
RP11-517C16.4	*TLDC1*	antisense	protein_coding	-2.36	-1.41	HPV16+/HPV16-
RP11-625H11.2	*TLN2*	antisense	protein_coding	2.95	-0.40	tumors/normal
RP11-315O6.1	*TMEM123*	antisense	protein_coding	-1.16	-0.66	HPV16+/HPV16-
TMEM161B-AS1	*TMEM161B*	antisense	protein_coding	1.10	0.40	HPV16+/HPV16-
TMEM220-AS1	*TMEM220*	antisense	protein_coding	-2.56	-1.94	tumors/normal
RP5-1120P11.1	*TMEM63B*	antisense	protein_coding	1.05	-0.37	HPV16+/HPV16-
TMEM9B-AS1	*TMEM9B*	antisense	protein_coding	-1.24	-0.11	HPV16+/HPV16-
CTD-2184D3.5	*TMOD3*	antisense	protein_coding	-2.20	-0.17	HPV16+/HPV16-
RP11-56B16.4	*TMOD3*	antisense	protein_coding	-1.16	-0.72	tumors/normal
TMPO-AS1	*TMPO*	antisense	protein_coding	1.71	1.56	HPV16+/HPV16-

RP11-875O11.3	*TNFRSF10B*	antisense	protein_coding	3.12	0.66	tumors/normal
CTB-129P6.4	*TOMM40*	antisense	protein_coding	-1.37	0.53	tumors/normal
RP11-347C18.5	*TP53INP1*	antisense	protein_coding	2.20	0.47	HPV16+/HPV16-
RP11-244F12.2	*TPM1*	antisense	protein_coding	-1.73	-1.29	HPV16+/HPV16-
CTD-2589H19.6	*TPPP*	antisense	protein_coding	1.41	-1.84	tumors/normal
TPRG1-AS2	*TPRG1*	antisense	protein_coding	-2.14	-1.73	tumors/normal
RP11-326K13.4	*TRAPPC8*	antisense	protein_coding	-1.14	-0.30	tumors/normal
RP11-97C16.1	*TRNT1*	antisense	protein_coding	1.52	0.57	HPV16+/HPV16-
RP5-823G15.5	*TSHZ2*	antisense	protein_coding	3.44	-1.11	tumors/normal
RP4-533D7.5	*TSPAN1*	antisense	protein_coding	2.48	1.43	HPV16+/HPV16-
RP11-137H2.4	*TSPAN14*	antisense	protein_coding	1.03	0.13	HPV16+/HPV16-
TTC39A-AS1	*TTC39A*	antisense	protein_coding	-3.58	-1.13	tumors/normal
CTD-2058B24.2	*TTC6*	antisense	protein_coding	-2.79	2.01	HPV16+/HPV16-
CTD-2537I9.12	*U2AF2*	antisense	protein_coding	1.71	0.36	tumors/normal
UBE2R2-AS1	*UBE2R2*	antisense	protein_coding	-1.21	-0.27	HPV16+/HPV16-
CTB-50L17.5	*UBXN6*	antisense	protein_coding	1.49	0.13	HPV16+/HPV16-
ADORA2A-AS1	*UPB1*	antisense	protein_coding	2.77	1.16	HPV16+/HPV16-
AP000355.2	*UPB1*	antisense	protein_coding	3.94	1.16	HPV16+/HPV16-
RP11-334E6.3	*USP2*	antisense	protein_coding	-3.51	-2.84	tumors/normal
RP11-162J8.3	*UST*	antisense	protein_coding	2.75	0.83	tumors/normal
RP3-467L1.4	*UTS2*	antisense	protein_coding	1.88	1.31	HPV16+/HPV16-
RP11-535A19.2	*UVRAG*	antisense	protein_coding	-2.46	0.79	tumors/normal
VCAN-AS1	*VCAN*	antisense	protein_coding	-1.27	-1.49	HPV16+/HPV16-
VPS9D1-AS1	*VPS9D1*	antisense	protein_coding	-1.68	0.17	HPV16+/HPV16-
RP11-136L23.2	*VSIG10*	antisense	protein_coding	1.14	0.22	HPV16+/HPV16-

RP11-136L23.2	*VSIG10*	antisense	protein_coding	1.14	0.46	HPV16+/HPV16-
CTD-2616J11.16	*VSIG10L*	antisense	protein_coding	-2.72	-2.54	tumors/normal
RP11-638I2.9	*WARS*	antisense	protein_coding	2.68	1.05	tumors/normal
WDR11-AS1	*WDR11*	antisense	protein_coding	1.41	0.09	HPV16+/HPV16-
RP11-638I2.9	*WDR25*	antisense	protein_coding	2.68	0.16	tumors/normal
AL022341.3	*WDR90*	antisense	protein_coding	-1.33	0.68	HPV16+/HPV16-
RP11-33N14.5	*WSB2*	antisense	protein_coding	3.40	0.28	HPV16+/HPV16-
RP11-460I13.2	*XKR8*	antisense	protein_coding	3.05	0.10	HPV16+/HPV16-
CTD-2576D5.4	*XYLT1*	antisense	protein_coding	1.48	-0.06	HPV16+/HPV16-
RP11-864G5.3	*YAP1*	antisense	protein_coding	-1.43	-1.25	HPV16+/HPV16-
RP11-278C7.4	*YARS2*	antisense	protein_coding	1.09	-0.27	HPV16+/HPV16-
YEATS2-AS1	*YEATS2*	antisense	protein_coding	1.44	0.93	tumors/normal
MLK7-AS1	*ZAK*	antisense	protein_coding	-1.41	-0.92	HPV16+/HPV16-
RP4-613B23.1	*ZBTB47*	antisense	protein_coding	-2.07	-1.69	tumors/normal
RP11-342M3.5	*ZCCHC24*	antisense	protein_coding	-1.93	-1.26	HPV16+/HPV16-
RP11-615I2.1	*ZFP90*	antisense	protein_coding	2.64	0.17	tumors/normal
ZFY-AS1	*ZFY*	antisense	protein_coding	2.14	1.85	HPV16+/HPV16-
CTC-453G23.8	*ZNF114*	antisense	protein_coding	1.27	-0.89	HPV16+/HPV16-
CTD-2035E11.3	*ZNF131*	antisense	protein_coding	1.29	0.31	HPV16+/HPV16-
CTC-559E9.8	*ZNF253*	antisense	protein_coding	1.03	0.55	HPV16+/HPV16-
VPS9D1-AS1	*ZNF276*	antisense	protein_coding	-1.68	0.28	HPV16+/HPV16-
RP4-694B14.8	*ZNF337*	antisense	protein_coding	1.59	0.54	HPV16+/HPV16-
RP11-944L7.4	*ZNF35*	antisense	protein_coding	-2.12	-0.61	tumors/normal
CTD-2525I3.6	*ZNF480*	antisense	protein_coding	1.48	0.29	HPV16+/HPV16-
RP11-411B10.3	*ZNF519*	antisense	protein_coding	2.97	0.48	HPV16+/HPV16-

CTD-2630F21.1	*ZNF566*	antisense	protein_coding	2.99	-0.10	tumors/normal
CTD-3064H18.1	*ZNF571*	antisense	protein_coding	-3.19	-0.41	tumors/normal
RP5-855D21.1	*ZNF596*	antisense	protein_coding	1.37	0.61	HPV16+/HPV16-
RP11-617F23.1	*ZNF710*	antisense	protein_coding	-1.49	-0.24	tumors/normal
CTD-2525I3.6	*ZNF766*	antisense	protein_coding	1.48	0.37	HPV16+/HPV16-
CTD-3064H18.1	*ZNF793*	antisense	protein_coding	-3.19	0.11	tumors/normal
RP11-234K24.6	*AAR2*	lncRNA	protein_coding	-3.06	0.16	tumors/normal
SOX21-AS1	*ABCC4*	lncRNA	protein_coding	1.40	0.08	HPV16+/HPV16-
CTD-2555K7.2	*ABHD4*	lncRNA	protein_coding	1.26	0.14	HPV16+/HPV16-
CTD-2619J13.13	*AC012313.1*	lncRNA	protein_coding	-2.26	-2.16	tumors/normal
ACAP2-IT1	*ACAP2*	lncRNA	protein_coding	1.15	0.68	HPV16+/HPV16-
RP11-774O3.3	*ACOX3*	lncRNA	protein_coding	-2.27	-0.69	tumors/normal
RP11-701P16.5	*ACSL1*	lncRNA	protein_coding	-1.56	-0.66	HPV16+/HPV16-
RP11-999E24.3	*ACTR10*	lncRNA	protein_coding	-2.94	-0.29	tumors/normal
RP11-445N20.3	*ACTR3C*	lncRNA	protein_coding	2.78	0.33	HPV16+/HPV16-
TEX41	*ACVR2A*	lncRNA	protein_coding	-1.08	0.52	HPV16+/HPV16-
RP11-890B15.2	*ADAMTS15*	lncRNA	protein_coding	-1.09	-3.34	HPV16+/HPV16-
RP11-398B16.2	*ADARB2*	lncRNA	protein_coding	-3.60	-1.16	tumors/normal
RP11-571L19.8	*ADH5*	lncRNA	protein_coding	1.06	0.49	HPV16+/HPV16-
RP11-1293J14.1	*ADI1*	lncRNA	protein_coding	1.77	0.56	HPV16+/HPV16-
CTD-3157E16.2	*ADORA2B*	lncRNA	protein_coding	-1.62	0.69	tumors/normal
AC008074.4	*AFTPH*	lncRNA	protein_coding	-2.68	-0.41	tumors/normal
LINC01099	*AGA*	lncRNA	protein_coding	2.57	-0.53	HPV16+/HPV16-
RP4-789D17.5	*AGO1*	lncRNA	protein_coding	1.29	0.22	HPV16+/HPV16-
RP4-789D17.5	*AGO3*	lncRNA	protein_coding	1.29	-0.05	HPV16+/HPV16-

RP11-8L2.1	*AGPAT9*	lncRNA	protein_coding	-2.39	-1.25	HPV16+/HPV16-
AC019117.2	*AHR*	lncRNA	protein_coding	-2.68	-1.10	HPV16+/HPV16-
RP11-439L18.2	*AIG1*	lncRNA	protein_coding	-1.19	0.15	tumors/normal
CTD-2555O16.3	*AKAP5*	lncRNA	protein_coding	2.38	0.70	HPV16+/HPV16-
RP11-38M8.1	*AKR1B1*	lncRNA	protein_coding	2.56	0.21	tumors/normal
LINC00704	*AKR1E2*	lncRNA	protein_coding	-3.26	1.82	HPV16+/HPV16-
RP11-93K22.13	*ALG1L2*	lncRNA	protein_coding	1.69	1.13	HPV16+/HPV16-
AC017002.2	*ANAPC1*	lncRNA	protein_coding	1.79	0.54	tumors/normal
RP11-200A1.1	*ANAPC13*	lncRNA	protein_coding	3.11	-0.14	HPV16+/HPV16-
RP11-7F17.7	*ANGEL1*	lncRNA	protein_coding	-2.78	-0.50	HPV16+/HPV16-
GS1-166A23.1	*ANKMY2*	lncRNA	protein_coding	-2.47	0.10	HPV16+/HPV16-
LINC01337	*ANKRD34B*	lncRNA	protein_coding	3.40	3.71	HPV16+/HPV16-
AC159540.2	*ANKRD36B*	lncRNA	protein_coding	2.30	0.40	HPV16+/HPV16-
LINC00342	*ANKRD36C*	lncRNA	protein_coding	1.56	1.05	HPV16+/HPV16-
LINC01091	*ANKRD50*	lncRNA	protein_coding	-2.59	-0.01	tumors/normal
RP4-758J18.13	*ANKRD65*	lncRNA	protein_coding	1.23	0.06	tumors/normal
CTD-2555C10.3	*ANKRD9*	lncRNA	protein_coding	-2.91	-0.24	HPV16+/HPV16-
LINC01094	*ANXA3*	lncRNA	protein_coding	-1.38	-0.92	HPV16+/HPV16-
AP006621.6	*AP006621.5*	lncRNA	protein_coding	1.40	-0.02	HPV16+/HPV16-
RP11-73K9.3	*AP1AR*	lncRNA	protein_coding	1.70	0.25	HPV16+/HPV16-
RP11-108K3.1	*AP4E1*	lncRNA	protein_coding	2.44	0.36	tumors/normal
APCDD1L-AS1	*APCDD1L*	lncRNA	protein_coding	-4.45	-3.71	HPV16+/HPV16-
RP11-932O9.10	*ARHGAP11B*	lncRNA	protein_coding	1.83	0.93	HPV16+/HPV16-
LINC00668	*ARHGAP28*	lncRNA	protein_coding	-1.44	0.10	HPV16+/HPV16-
RP11-10O17.3	*ARID3B*	lncRNA	protein_coding	2.12	0.03	HPV16+/HPV16-

AC011891.5	*ARL4A*	lncRNA	protein_coding	-4.04	-1.01	tumors/normal
AC011288.2	*ARL4A*	lncRNA	protein_coding	3.41	-1.01	tumors/normal
AC010148.1	*ARL4C*	lncRNA	protein_coding	1.79	0.47	tumors/normal
MIR2117	*ARL4D*	lncRNA	protein_coding	-1.77	-1.74	HPV16+/HPV16-
CTD-2501M5.1	*ASAP1*	lncRNA	protein_coding	-1.77	-0.15	HPV16+/HPV16-
RP11-336A10.4	*ASB13*	lncRNA	protein_coding	-1.49	0.06	HPV16+/HPV16-
LINC01290	*ATF7IP2*	lncRNA	protein_coding	1.58	0.92	HPV16+/HPV16-
RP11-1100L3.8	*ATG101*	lncRNA	protein_coding	-1.97	0.11	tumors/normal
RP11-303E16.2	*ATMIN*	lncRNA	protein_coding	1.40	-0.45	tumors/normal
RP11-823E8.3	*ATP2B1*	lncRNA	protein_coding	-1.56	-0.49	HPV16+/HPV16-
RP11-297D21.4	*ATP6V0D1*	lncRNA	protein_coding	1.04	-0.58	HPV16+/HPV16-
CTA-392E5.1	*ATP6V0D2*	lncRNA	protein_coding	-4.87	-0.29	HPV16+/HPV16-
RP11-445N20.3	*ATP6V0E2*	lncRNA	protein_coding	2.78	1.42	HPV16+/HPV16-
AC005256.1	*ATP8B3*	lncRNA	protein_coding	-3.16	-0.40	HPV16+/HPV16-
RP11-176N18.2	*ATP9B*	lncRNA	protein_coding	-1.57	0.29	HPV16+/HPV16-
RP11-245J9.5	*ATXN7*	lncRNA	protein_coding	1.13	0.52	HPV16+/HPV16-
KB-1732A1.1	*AZIN1*	lncRNA	protein_coding	-2.25	-0.40	HPV16+/HPV16-
AC093159.1	*B3GNT2*	lncRNA	protein_coding	-3.63	-0.57	tumors/normal
CTC-435M10.12	*B9D2*	lncRNA	protein_coding	1.63	0.96	HPV16+/HPV16-
RP11-863P13.3	*BANP*	lncRNA	protein_coding	1.82	0.49	tumors/normal
RP11-54A9.1	*BBS10*	lncRNA	protein_coding	-2.12	-0.10	HPV16+/HPV16-
RP11-250B2.3	*BCKDHB*	lncRNA	protein_coding	1.47	0.86	HPV16+/HPV16-
RP11-28F1.2	*BCL2*	lncRNA	protein_coding	1.96	2.14	HPV16+/HPV16-
CTD-2184D3.3	*BCL2L10*	lncRNA	protein_coding	-3.72	-0.24	HPV16+/HPV16-
AC108463.1	*BCL2L11*	lncRNA	protein_coding	3.12	-0.09	tumors/normal

RP11-567G11.1	*BCL6*	lncRNA	protein_coding	-4.20	0.19	HPV16+/HPV16-
AC156455.1	*BCL7A*	lncRNA	protein_coding	2.45	0.15	tumors/normal
RP11-403P17.3	*BEAN1*	lncRNA	protein_coding	-1.11	0.41	tumors/normal
CTD-3094K11.1	*BLM*	lncRNA	protein_coding	-1.38	0.51	HPV16+/HPV16-
LINC01094	*BMP2K*	lncRNA	protein_coding	-1.38	-0.27	HPV16+/HPV16-
RP4-813D12.3	*BMP7*	lncRNA	protein_coding	1.08	0.81	HPV16+/HPV16-
RP11-681L8.1	*BMPR1B*	lncRNA	protein_coding	-1.84	-2.51	HPV16+/HPV16-
CTB-33O18.1	*BOD1*	lncRNA	protein_coding	-4.17	0.06	HPV16+/HPV16-
RP11-21B23.2	*BRD7*	lncRNA	protein_coding	-3.58	-0.37	HPV16+/HPV16-
RP11-44N21.1	*BRF1*	lncRNA	protein_coding	1.35	0.17	tumors/normal
RP11-399K21.14	*C10orf11*	lncRNA	protein_coding	1.47	-0.12	tumors/normal
RP11-216P16.8	*C12orf43*	lncRNA	protein_coding	3.72	0.12	tumors/normal
RP11-999E24.3	*C14orf105*	lncRNA	protein_coding	-2.94	-0.03	tumors/normal
CTD-2591A6.2	*C14orf23*	lncRNA	protein_coding	5.70	0.46	tumors/normal
RP11-568J23.8	*C16orf74*	lncRNA	protein_coding	3.51	1.87	tumors/normal
RP11-855A2.5	*C17orf58*	lncRNA	protein_coding	2.04	0.63	HPV16+/HPV16-
AC005532.5	*C1GALT1*	lncRNA	protein_coding	-2.48	0.02	tumors/normal
RP5-1057J7.7	*C1orf213*	lncRNA	protein_coding	1.75	0.54	HPV16+/HPV16-
LINC00302	*C1orf68*	lncRNA	protein_coding	-3.95	-3.38	HPV16+/HPV16-
RP11-745L13.2	*C4orf33*	lncRNA	protein_coding	1.33	0.33	HPV16+/HPV16-
C5orf66-AS1	*C5orf66*	lncRNA	protein_coding	4.06	1.44	HPV16+/HPV16-
RP11-351J23.1	*C6orf123*	lncRNA	protein_coding	-5.59	0.06	tumors/normal
RP11-7K24.3	*C6orf132*	lncRNA	protein_coding	-1.77	-1.23	tumors/normal
CTA-392E5.1	*CA2*	lncRNA	protein_coding	-4.87	-1.49	HPV16+/HPV16-
ESRG	*CACNA2D3*	lncRNA	protein_coding	-1.92	0.30	HPV16+/HPV16-

RABGAP1L-IT1	*CACYBP*	lncRNA	protein_coding	1.19	0.36	tumors/normal
RP11-338N10.3	*CAMTA1*	lncRNA	protein_coding	1.87	0.56	HPV16+/HPV16-
RP11-212I21.2	*CAPNS2*	lncRNA	protein_coding	1.87	-0.59	HPV16+/HPV16-
LINC00941	*CAPRIN2*	lncRNA	protein_coding	-1.66	-0.34	HPV16+/HPV16-
AC026806.2	*CATSPERG*	lncRNA	protein_coding	-1.14	0.00	HPV16+/HPV16-
RP11-875H7.5	*CCDC66*	lncRNA	protein_coding	-5.16	-0.17	tumors/normal
RP5-884M6.1	*CCDC71L*	lncRNA	protein_coding	-1.95	-0.88	HPV16+/HPV16-
LINC01279	*CCDC80*	lncRNA	protein_coding	-1.62	-0.92	tumors/normal
CTD-2547L24.4	*CCDC88C*	lncRNA	protein_coding	-1.94	0.34	HPV16+/HPV16-
CTB-186H2.3	*CCL16*	lncRNA	protein_coding	-2.66	0.56	tumors/normal
CTB-186H2.3	*CCL18*	lncRNA	protein_coding	-2.66	0.90	tumors/normal
AC015849.16	*CCL5*	lncRNA	protein_coding	2.41	0.60	HPV16+/HPV16-
RP5-1028K7.2	*CCR7*	lncRNA	protein_coding	2.58	0.03	tumors/normal
AF129075.5	*CCT8*	lncRNA	protein_coding	1.19	0.09	HPV16+/HPV16-
CTC-490G23.2	*CD177*	lncRNA	protein_coding	-2.29	-0.05	HPV16+/HPV16-
RP11-104L21.2	*CD247*	lncRNA	protein_coding	-1.98	0.27	tumors/normal
RP11-214C8.2	*CD79B*	lncRNA	protein_coding	1.32	-0.98	tumors/normal
RP11-330A16.1	*CD83*	lncRNA	protein_coding	1.99	0.39	HPV16+/HPV16-
CDC37L1-AS1	*CDC37L1*	lncRNA	protein_coding	1.24	0.05	HPV16+/HPV16-
RP11-736N17.8	*CDC42BPB*	lncRNA	protein_coding	2.15	-0.65	HPV16+/HPV16-
RP11-256I9.2	*CDH11*	lncRNA	protein_coding	-2.27	-1.83	HPV16+/HPV16-
CTD-2023M8.1	*CDH18*	lncRNA	protein_coding	5.88	-0.53	tumors/normal
RP5-1043L13.1	*CDH26*	lncRNA	protein_coding	-1.24	0.83	HPV16+/HPV16-
RP11-256I9.2	*CDH5*	lncRNA	protein_coding	-2.27	-0.47	HPV16+/HPV16-
RP11-131N11.4	*CDK1*	lncRNA	protein_coding	2.16	1.14	HPV16+/HPV16-

RP11-180M15.7	*CDKN1B*	lncRNA	protein_coding	1.26	0.72	HPV16+/HPV16-
RP11-149I2.4	*CDKN2A*	lncRNA	protein_coding	2.73	5.09	HPV16+/HPV16-
RP11-215P8.4	*CDKN2AIPNL*	lncRNA	protein_coding	3.97	0.13	tumors/normal
RP11-149I2.4	*CDKN2B*	lncRNA	protein_coding	2.73	1.43	HPV16+/HPV16-
RP11-8L2.1	*CDS1*	lncRNA	protein_coding	-2.39	-0.30	HPV16+/HPV16-
RP4-529N6.2	*CDYL*	lncRNA	protein_coding	-2.21	-0.40	HPV16+/HPV16-
RP11-328K4.1	*CENPE*	lncRNA	protein_coding	-4.81	0.17	HPV16+/HPV16-
CTD-2116N20.1	*CENPK*	lncRNA	protein_coding	3.90	1.04	tumors/normal
RP11-303E16.2	*CENPN*	lncRNA	protein_coding	1.40	1.00	tumors/normal
RP11-167P11.2	*CENPT*	lncRNA	protein_coding	1.42	-0.26	HPV16+/HPV16-
RP11-339B21.10	*CERCAM*	lncRNA	protein_coding	-1.50	-1.43	HPV16+/HPV16-
LINC00926	*CGNL1*	lncRNA	protein_coding	1.06	0.72	HPV16+/HPV16-
RP11-140I16.3	*CHCHD7*	lncRNA	protein_coding	1.70	-0.29	HPV16+/HPV16-
RP11-295M3.4	*CHD9*	lncRNA	protein_coding	-2.23	-0.12	tumors/normal
LINC00971	*CHMP2B*	lncRNA	protein_coding	1.08	0.13	HPV16+/HPV16-
RP11-80H8.4	*CHST2*	lncRNA	protein_coding	3.38	2.51	tumors/normal
RP11-424I19.2	*CHSY1*	lncRNA	protein_coding	-1.62	-0.77	HPV16+/HPV16-
LINC01305	*CIR1*	lncRNA	protein_coding	5.16	0.13	HPV16+/HPV16-
RP3-460G2.2	*CITED2*	lncRNA	protein_coding	-1.71	0.00	HPV16+/HPV16-
AC091814.3	*CLEC12A*	lncRNA	protein_coding	4.56	2.03	tumors/normal
AC091814.3	*CLEC2B*	lncRNA	protein_coding	4.56	0.40	tumors/normal
RP11-90D4.3	*CLEC4E*	lncRNA	protein_coding	1.77	-0.06	HPV16+/HPV16-
RP11-231E4.2	*CNDP2*	lncRNA	protein_coding	1.08	0.24	HPV16+/HPV16-
RP11-353K11.1	*CNNM3*	lncRNA	protein_coding	1.47	0.55	HPV16+/HPV16-
RP11-353K11.1	*CNNM4*	lncRNA	protein_coding	1.47	-0.06	HPV16+/HPV16-

RP11-320P7.2	*CNOT2*	lncRNA	protein_coding	3.98	0.32	tumors/normal
RP11-242F4.2	*CNOT7*	lncRNA	protein_coding	1.21	0.29	HPV16+/HPV16-
RP11-760H22.2	*COL14A1*	lncRNA	protein_coding	-3.90	-1.63	tumors/normal
LL21NC02-21A1.1	*COL18A1*	lncRNA	protein_coding	-1.19	-0.80	HPV16+/HPV16-
AC005532.5	*COL28A1*	lncRNA	protein_coding	-2.48	0.70	tumors/normal
RP5-1159O4.1	*COL28A1*	lncRNA	protein_coding	-1.65	0.70	tumors/normal
AC112721.2	*COL6A3*	lncRNA	protein_coding	4.72	2.34	tumors/normal
RP11-805L22.3	*COPRS*	lncRNA	protein_coding	2.71	0.03	tumors/normal
RP11-18C24.8	*COQ5*	lncRNA	protein_coding	1.24	0.40	HPV16+/HPV16-
RP11-626G11.6	*COQ7*	lncRNA	protein_coding	2.32	0.10	HPV16+/HPV16-
CTC-480C2.1	*COX7C*	lncRNA	protein_coding	-1.79	0.62	HPV16+/HPV16-
RP11-104L21.3	*CREG1*	lncRNA	protein_coding	-2.76	-0.68	tumors/normal
RP11-490M8.1	*CRIM1*	lncRNA	protein_coding	-2.28	-1.52	HPV16+/HPV16-
CTD-2349P21.10	*CRLF3*	lncRNA	protein_coding	1.19	0.43	HPV16+/HPV16-
RP5-967N21.11	*CRLS1*	lncRNA	protein_coding	1.41	-0.31	HPV16+/HPV16-
RP11-107M16.2	*CRNN*	lncRNA	protein_coding	-2.91	0.25	HPV16+/HPV16-
RP4-660H19.1	*CRYZ*	lncRNA	protein_coding	-2.99	-0.05	HPV16+/HPV16-
RP11-1136G11.8	*CSAD*	lncRNA	protein_coding	1.77	0.93	HPV16+/HPV16-
RP11-387H17.6	*CSF3*	lncRNA	protein_coding	1.90	-1.82	tumors/normal
RP4-568C11.4	*CST7*	lncRNA	protein_coding	1.28	0.56	HPV16+/HPV16-
RP11-383C5.3	*CTBP2*	lncRNA	protein_coding	2.13	-0.05	tumors/normal
RP11-849F2.8	*CTC1*	lncRNA	protein_coding	1.12	0.44	HPV16+/HPV16-
CTD-2547L24.4	*CTD-2547L24.3*	lncRNA	protein_coding	-1.94	-0.18	HPV16+/HPV16-
RP11-248M19.1	*CTDP1*	lncRNA	protein_coding	1.15	0.46	HPV16+/HPV16-
RP11-620J15.3	*CTDSP2*	lncRNA	protein_coding	1.04	0.10	HPV16+/HPV16-

RP11-571M6.18	*CTDSP2*	lncRNA	protein_coding	1.21	0.10	HPV16+/HPV16-
RP11-69I8.2	*CTGF*	lncRNA	protein_coding	-1.69	-1.06	HPV16+/HPV16-
RP5-965F6.2	*CTTNBP2NL*	lncRNA	protein_coding	-4.18	-0.43	tumors/normal
RP11-307L14.1	*CWC27*	lncRNA	protein_coding	1.46	0.38	HPV16+/HPV16-
RP11-119J18.1	*CXCL14*	lncRNA	protein_coding	-4.27	-3.85	HPV16+/HPV16-
CTB-50E14.4	*CXCL17*	lncRNA	protein_coding	3.34	2.99	HPV16+/HPV16-
RP11-231E4.2	*CYB5A*	lncRNA	protein_coding	1.08	0.34	HPV16+/HPV16-
AC003090.1	*CYCS*	lncRNA	protein_coding	-4.85	-0.41	tumors/normal
RP11-108K3.2	*CYP19A1*	lncRNA	protein_coding	3.52	1.25	tumors/normal
RP11-776H12.1	*CYP2J2*	lncRNA	protein_coding	-2.64	0.67	HPV16+/HPV16-
CTD-2357A8.3	*CYTH1*	lncRNA	protein_coding	5.99	0.14	tumors/normal
RP6-114E22.1	*DCAF4*	lncRNA	protein_coding	-2.53	-0.61	HPV16+/HPV16-
RP11-365O16.3	*DEGS1*	lncRNA	protein_coding	1.04	-0.84	HPV16+/HPV16-
RP11-760H22.2	*DEPTOR*	lncRNA	protein_coding	-3.90	-3.14	tumors/normal
RP11-44N11.1	*DERL1*	lncRNA	protein_coding	2.37	0.34	tumors/normal
RP11-876N24.7	*DEXI*	lncRNA	protein_coding	1.80	-0.12	HPV16+/HPV16-
LINC01337	*DHFR*	lncRNA	protein_coding	3.40	1.76	HPV16+/HPV16-
MIR2117	*DHX8*	lncRNA	protein_coding	-1.77	0.18	HPV16+/HPV16-
RP11-346D6.6	*DKK1*	lncRNA	protein_coding	-6.25	-3.15	HPV16+/HPV16-
AC128709.3	*DLG1*	lncRNA	protein_coding	-2.27	0.21	HPV16+/HPV16-
AC104088.1	*DLX1*	lncRNA	protein_coding	-1.97	-4.43	HPV16+/HPV16-
RP5-850O15.4	*DMRTA2*	lncRNA	protein_coding	2.95	4.48	HPV16+/HPV16-
RP11-108K3.2	*DMXL2*	lncRNA	protein_coding	3.52	0.62	tumors/normal
AC012074.2	*DNMT3A*	lncRNA	protein_coding	1.26	0.78	HPV16+/HPV16-
CTB-102L5.8	*DPF1*	lncRNA	protein_coding	1.08	-1.64	HPV16+/HPV16-

RP11-631N16.4	*DPY19L2*	lncRNA	protein_coding	2.47	0.23	tumors/normal
RP11-650P15.1	*DSG2*	lncRNA	protein_coding	-2.36	1.15	tumors/normal
RP11-650P15.1	*DSG3*	lncRNA	protein_coding	-2.36	0.19	tumors/normal
RP11-15I11.2	*DTL*	lncRNA	protein_coding	1.07	1.17	HPV16+/HPV16-
RP11-96A15.1	*DTNA*	lncRNA	protein_coding	-2.91	-0.96	HPV16+/HPV16-
AC012074.2	*DTNB*	lncRNA	protein_coding	1.26	0.51	HPV16+/HPV16-
CTD-3179P9.2	*DTWD2*	lncRNA	protein_coding	3.75	-0.35	tumors/normal
CTC-501O10.1	*DUSP3*	lncRNA	protein_coding	1.06	-0.31	HPV16+/HPV16-
RP11-525A16.4	*DUSP5*	lncRNA	protein_coding	-2.17	-0.36	HPV16+/HPV16-
RP11-823E8.3	*DUSP6*	lncRNA	protein_coding	-1.56	-1.57	HPV16+/HPV16-
RP11-110H1.4	*DYM*	lncRNA	protein_coding	1.63	0.54	HPV16+/HPV16-
AP001434.2	*DYRK1A*	lncRNA	protein_coding	-2.17	0.57	HPV16+/HPV16-
LLNLR-268E12.1	*DYRK1B*	lncRNA	protein_coding	2.02	0.89	HPV16+/HPV16-
RP4-529N6.2	*ECI2*	lncRNA	protein_coding	-2.21	-0.64	HPV16+/HPV16-
LINC00568	*ECM1*	lncRNA	protein_coding	-1.83	-1.10	tumors/normal
RP11-383C5.5	*EDRF1*	lncRNA	protein_coding	1.88	0.21	HPV16+/HPV16-
LINC00460	*EFNB2*	lncRNA	protein_coding	-4.48	-1.71	HPV16+/HPV16-
CTD-2501M5.1	*EFR3A*	lncRNA	protein_coding	-1.77	-0.35	HPV16+/HPV16-
RP11-499F3.2	*EFTUD1*	lncRNA	protein_coding	-2.77	-0.16	HPV16+/HPV16-
LLNLR-268E12.1	*EID2*	lncRNA	protein_coding	2.02	-0.15	HPV16+/HPV16-
RP11-153F5.7	*EIF4B*	lncRNA	protein_coding	1.94	0.27	HPV16+/HPV16-
RP11-475O6.1	*ELTD1*	lncRNA	protein_coding	-1.18	-0.43	tumors/normal
CTD-2013M15.1	*EMB*	lncRNA	protein_coding	2.60	-1.01	tumors/normal
LINC01290	*EMP2*	lncRNA	protein_coding	1.58	0.85	HPV16+/HPV16-
CTC-241F20.4	*EMP3*	lncRNA	protein_coding	2.65	0.91	tumors/normal

AC093901.1	*EN1*	lncRNA	protein_coding	1.24	-2.78	HPV16+/HPV16-
RP11-69I8.2	*ENPP1*	lncRNA	protein_coding	-1.69	0.33	HPV16+/HPV16-
RP11-234K24.6	*EPB41L1*	lncRNA	protein_coding	-3.06	-1.24	tumors/normal
RP4-550H1.5	*EPB41L1*	lncRNA	protein_coding	1.24	0.46	HPV16+/HPV16-
BCYRN1	*EPCAM*	lncRNA	protein_coding	-1.04	0.38	HPV16+/HPV16-
RP11-657O9.1	*EPHB1*	lncRNA	protein_coding	-4.36	-0.01	HPV16+/HPV16-
RP11-129K12.3	*EPM2AIP1*	lncRNA	protein_coding	1.87	0.42	HPV16+/HPV16-
SNORA76C	*ERN1*	lncRNA	protein_coding	1.36	-0.40	tumors/normal
RP11-114G22.1	*ETNK1*	lncRNA	protein_coding	2.56	-0.27	HPV16+/HPV16-
AC011288.2	*ETV1*	lncRNA	protein_coding	3.41	-1.15	tumors/normal
LINC01252	*ETV6*	lncRNA	protein_coding	1.65	0.03	tumors/normal
RP11-736N17.8	*EXOC3L4*	lncRNA	protein_coding	2.15	1.76	HPV16+/HPV16-
CTC-435M10.12	*EXOSC5*	lncRNA	protein_coding	1.63	0.59	HPV16+/HPV16-
RP11-120K24.5	*F10*	lncRNA	protein_coding	2.47	-0.59	HPV16+/HPV16-
RP5-850O15.4	*FAF1*	lncRNA	protein_coding	2.95	0.79	HPV16+/HPV16-
RP11-462G2.1	*FAM135A*	lncRNA	protein_coding	-1.97	-0.15	HPV16+/HPV16-
RP11-1252I4.2	*FAM153A*	lncRNA	protein_coding	2.15	1.63	HPV16+/HPV16-
AC009784.3	*FAM180A*	lncRNA	protein_coding	-2.19	-1.57	HPV16+/HPV16-
RP11-1277A3.3	*FAM193B*	lncRNA	protein_coding	1.14	0.62	HPV16+/HPV16-
AC093627.10	*FAM20C*	lncRNA	protein_coding	2.16	-0.54	HPV16+/HPV16-
LINC01160	*FAM212B*	lncRNA	protein_coding	3.40	0.06	tumors/normal
RP1-93H18.7	*FAM26F*	lncRNA	protein_coding	-1.45	0.68	HPV16+/HPV16-
RP11-374M1.5	*FAM27C*	lncRNA	protein_coding	3.09	-0.19	tumors/normal
AC090505.6	*FAM43A*	lncRNA	protein_coding	1.42	-0.55	HPV16+/HPV16-
RP11-250B2.3	*FAM46A*	lncRNA	protein_coding	1.47	-0.49	HPV16+/HPV16-

RP11-1398P2.1	*FAM53A*	lncRNA	protein_coding	-1.02	0.91	HPV16+/HPV16-
RP11-297D21.4	*FAM65A*	lncRNA	protein_coding	1.04	-0.44	HPV16+/HPV16-
RP11-338H14.1	*FAM76B*	lncRNA	protein_coding	2.88	0.44	tumors/normal
LA16c-380A1.1	*FBXL16*	lncRNA	protein_coding	-3.96	-1.00	tumors/normal
CTD-2354A18.1	*FBXO15*	lncRNA	protein_coding	-1.66	0.48	HPV16+/HPV16-
RP11-73G16.1	*FBXW7*	lncRNA	protein_coding	1.29	-0.41	tumors/normal
LINC-ROR	*FECH*	lncRNA	protein_coding	-1.31	0.37	HPV16+/HPV16-
AC002511.3	*FFAR2*	lncRNA	protein_coding	-3.02	0.39	tumors/normal
AC093850.2	*FN1*	lncRNA	protein_coding	6.29	2.85	tumors/normal
RP11-388C12.8	*FN3K*	lncRNA	protein_coding	1.54	-0.77	tumors/normal
RP11-388C12.5	*FN3K*	lncRNA	protein_coding	2.31	-0.77	tumors/normal
RP11-388C12.5	*FN3KRP*	lncRNA	protein_coding	2.31	0.37	tumors/normal
RP11-356O9.2	*FOXA1*	lncRNA	protein_coding	4.80	-3.26	tumors/normal
RP11-157J24.2	*FOXC1*	lncRNA	protein_coding	1.60	-0.68	HPV16+/HPV16-
RP11-60A8.1	*FOXD1*	lncRNA	protein_coding	-2.40	2.46	tumors/normal
RP11-23B15.1	*FOXE1*	lncRNA	protein_coding	1.80	1.13	HPV16+/HPV16-
RP11-157J24.2	*FOXF2*	lncRNA	protein_coding	1.60	-1.82	HPV16+/HPV16-
RP11-372B4.3	*FOXM1*	lncRNA	protein_coding	1.39	0.45	HPV16+/HPV16-
AF146191.4	*FRG1*	lncRNA	protein_coding	1.90	0.63	HPV16+/HPV16-
FAM182A	*FRG1B*	lncRNA	protein_coding	2.43	0.61	HPV16+/HPV16-
RP11-255G12.3	*FRMD6*	lncRNA	protein_coding	-3.86	-1.44	HPV16+/HPV16-
AC073043.1	*FTCDNL1*	lncRNA	protein_coding	1.10	-0.76	HPV16+/HPV16-
CTD-3094K11.1	*FURIN*	lncRNA	protein_coding	-1.38	-0.99	HPV16+/HPV16-
LINC00971	*GBE1*	lncRNA	protein_coding	1.08	0.47	HPV16+/HPV16-
GLYCTK-AS1	*GLYCTK*	lncRNA	protein_coding	-1.29	-0.69	tumors/normal

APCDD1L-AS1	*GNAS*	lncRNA	protein_coding	-4.45	0.16	HPV16+/HPV16-
RP11-182J1.13	*GOLGA6L4*	lncRNA	protein_coding	1.43	0.18	HPV16+/HPV16-
RP11-129J12.2	*GOT1*	lncRNA	protein_coding	4.52	0.50	HPV16+/HPV16-
RP11-44N21.1	*GPR132*	lncRNA	protein_coding	1.35	0.73	tumors/normal
LINC00337	*GPR153*	lncRNA	protein_coding	1.28	1.08	tumors/normal
RP3-510D11.2	*GPR157*	lncRNA	protein_coding	1.48	-0.44	HPV16+/HPV16-
RP11-94L15.2	*GRB7*	lncRNA	protein_coding	1.73	0.92	HPV16+/HPV16-
RP11-254F7.2	*GRHL1*	lncRNA	protein_coding	1.84	0.14	HPV16+/HPV16-
RP11-18H7.1	*GSK3B*	lncRNA	protein_coding	1.31	0.77	tumors/normal
RP3-508I15.20	*GTPBP1*	lncRNA	protein_coding	1.48	0.25	HPV16+/HPV16-
C5orf66-AS1	*H2AFY*	lncRNA	protein_coding	4.06	0.41	HPV16+/HPV16-
RP3-510D11.2	*H6PD*	lncRNA	protein_coding	1.48	0.10	HPV16+/HPV16-
LA16c-OS12.2	*HBA2*	lncRNA	protein_coding	1.37	-0.69	HPV16+/HPV16-
RP11-324E6.10	*HCAR2*	lncRNA	protein_coding	2.91	-0.01	HPV16+/HPV16-
RP11-733O18.1	*HDHD1*	lncRNA	protein_coding	-2.54	-0.42	HPV16+/HPV16-
RP11-407B7.1	*HES1*	lncRNA	protein_coding	-3.22	-0.62	tumors/normal
LINC00263	*HIF1AN*	lncRNA	protein_coding	-1.02	0.03	HPV16+/HPV16-
RP1-86C11.7	*HIST1H2BJ*	lncRNA	protein_coding	1.47	1.46	HPV16+/HPV16-
RP3-468B3.2	*HMGA1*	lncRNA	protein_coding	1.58	0.68	tumors/normal
CASC9	*HNF4G*	lncRNA	protein_coding	4.32	-0.10	tumors/normal
RP11-127B20.2	*HNRNPD*	lncRNA	protein_coding	2.23	0.42	HPV16+/HPV16-
AC009336.24	*HOXD8*	lncRNA	protein_coding	3.88	2.14	tumors/normal
RP11-131L12.3	*HSPB8*	lncRNA	protein_coding	1.04	-0.68	HPV16+/HPV16-
RP11-774O3.3	*HTRA3*	lncRNA	protein_coding	-2.27	2.23	tumors/normal
AC095067.1	*HUS1*	lncRNA	protein_coding	1.69	0.45	tumors/normal

RP11-214C8.2	*ICAM2*	lncRNA	protein_coding	1.32	-0.60	tumors/normal
LINC00337	*ICMT*	lncRNA	protein_coding	1.28	0.07	tumors/normal
AP001059.7	*ICOSLG*	lncRNA	protein_coding	1.69	0.70	HPV16+/HPV16-
RP11-44K6.2	*IDO1*	lncRNA	protein_coding	3.05	1.65	tumors/normal
AC006262.4	*IGFL2*	lncRNA	protein_coding	-4.77	-3.83	HPV16+/HPV16-
LINC01133	*IGSF9*	lncRNA	protein_coding	1.46	1.33	HPV16+/HPV16-
RP11-105N14.1	*IKZF2*	lncRNA	protein_coding	2.35	1.71	HPV16+/HPV16-
RP11-94L15.2	*IKZF3*	lncRNA	protein_coding	1.73	3.09	HPV16+/HPV16-
RP11-499F3.2	*IL16*	lncRNA	protein_coding	-2.77	0.58	HPV16+/HPV16-
AC093901.1	*INSIG2*	lncRNA	protein_coding	1.24	-0.50	HPV16+/HPV16-
RP11-290F5.1	*IRF2*	lncRNA	protein_coding	1.03	0.42	HPV16+/HPV16-
RP11-395N3.2	*IRS1*	lncRNA	protein_coding	-4.34	-2.27	HPV16+/HPV16-
LINC01067	*IRS2*	lncRNA	protein_coding	2.81	-1.22	tumors/normal
RP11-259O2.3	*IRX2*	lncRNA	protein_coding	-1.41	-3.06	HPV16+/HPV16-
RP5-875H18.9	*ITGA3*	lncRNA	protein_coding	-3.61	-1.25	HPV16+/HPV16-
AC104820.2	*ITGA4*	lncRNA	protein_coding	3.00	0.28	HPV16+/HPV16-
AC104088.1	*ITGA6*	lncRNA	protein_coding	-1.97	-1.26	HPV16+/HPV16-
RP11-462L8.1	*ITGB1*	lncRNA	protein_coding	-1.81	-0.72	HPV16+/HPV16-
RP11-71H17.9	*ITGB5*	lncRNA	protein_coding	-1.32	-0.90	HPV16+/HPV16-
GS1-279B7.2	*IVNS1ABP*	lncRNA	protein_coding	3.64	0.61	tumors/normal
RP11-215P8.4	*JADE2*	lncRNA	protein_coding	3.97	0.86	tumors/normal
RP11-422P24.11	*JTB*	lncRNA	protein_coding	1.04	0.36	HPV16+/HPV16-
RP4-794H19.1	*JUN*	lncRNA	protein_coding	-1.41	-0.21	HPV16+/HPV16-
RP11-108L7.15	*KAZALD1*	lncRNA	protein_coding	1.10	0.06	HPV16+/HPV16-
RP5-965F6.2	*KCND3*	lncRNA	protein_coding	-4.18	-0.99	tumors/normal

RP1-193H18.2	*KCNJ2*	lncRNA	protein_coding	1.90	0.54	HPV16+/HPV16-
AC026806.2	*KCNK6*	lncRNA	protein_coding	-1.14	-0.78	HPV16+/HPV16-
CTC-526N19.1	*KCTD15*	lncRNA	protein_coding	-1.75	-0.42	HPV16+/HPV16-
LINC00460	*KDELC1*	lncRNA	protein_coding	-4.48	-0.87	HPV16+/HPV16-
TTTY14	*KDM5D*	lncRNA	protein_coding	3.01	2.05	HPV16+/HPV16-
RP11-28F1.2	*KDSR*	lncRNA	protein_coding	1.96	-0.11	HPV16+/HPV16-
CTC-429L19.3	*KEAP1*	lncRNA	protein_coding	2.46	0.62	HPV16+/HPV16-
LINC00669	*KIAA1328*	lncRNA	protein_coding	1.90	-0.95	HPV16+/HPV16-
LINC00678	*KIF18A*	lncRNA	protein_coding	-1.86	0.43	HPV16+/HPV16-
KB-1732A1.1	*KLF10*	lncRNA	protein_coding	-2.25	-1.15	HPV16+/HPV16-
RP11-254F7.2	*KLF11*	lncRNA	protein_coding	1.84	1.11	HPV16+/HPV16-
RP11-16E12.2	*KLF13*	lncRNA	protein_coding	1.79	-0.17	HPV16+/HPV16-
AC007879.7	*KLF7*	lncRNA	protein_coding	-2.73	-1.61	HPV16+/HPV16-
RP11-277P12.10	*KLRD1*	lncRNA	protein_coding	-2.11	-1.22	HPV16+/HPV16-
RP11-324E6.10	*KNTC1*	lncRNA	protein_coding	2.91	1.14	HPV16+/HPV16-
AC145343.2	*KPNA2*	lncRNA	protein_coding	1.66	0.14	HPV16+/HPV16-
RP11-855A2.5	*KPNA2*	lncRNA	protein_coding	2.04	0.14	HPV16+/HPV16-
RP11-114H23.1	*KRR1*	lncRNA	protein_coding	-1.91	-0.17	HPV16+/HPV16-
LINC00592	*KRT7*	lncRNA	protein_coding	-1.83	1.13	HPV16+/HPV16-
RP11-153F5.7	*KRT8*	lncRNA	protein_coding	1.94	1.64	HPV16+/HPV16-
LINC00592	*KRT80*	lncRNA	protein_coding	-1.83	-0.75	HPV16+/HPV16-
RP11-69G7.1	*LACTB*	lncRNA	protein_coding	-3.29	-0.60	HPV16+/HPV16-
RP11-346D19.1	*LAMA4*	lncRNA	protein_coding	-3.37	-1.08	HPV16+/HPV16-
LINC00302	*LCE3A*	lncRNA	protein_coding	-3.95	-2.12	HPV16+/HPV16-
RP3-468B3.2	*LEMD2*	lncRNA	protein_coding	1.58	0.46	tumors/normal

AC008074.4	*LGALSL*	lncRNA	protein_coding	-2.68	-1.94	tumors/normal
CTD-2527I21.15	*LGI4*	lncRNA	protein_coding	4.13	-0.65	HPV16+/HPV16-
CTB-50E14.4	*LIPE*	lncRNA	protein_coding	3.34	1.01	HPV16+/HPV16-
RP11-110H1.8	*LIPG*	lncRNA	protein_coding	1.07	0.85	tumors/normal
RP11-462G2.1	*LMBRD1*	lncRNA	protein_coding	-1.97	0.01	HPV16+/HPV16-
RP11-410K21.2	*LPAR1*	lncRNA	protein_coding	1.15	-0.49	HPV16+/HPV16-
RP11-137H15.3	*LRP10*	lncRNA	protein_coding	1.27	-0.19	HPV16+/HPV16-
ZFPM2-AS1	*LRP12*	lncRNA	protein_coding	-2.94	-1.09	HPV16+/HPV16-
RP11-529E10.7	*LRPAP1*	lncRNA	protein_coding	1.32	-0.21	HPV16+/HPV16-
RP11-20G13.1	*LRRC28*	lncRNA	protein_coding	-1.84	0.01	HPV16+/HPV16-
RP11-18H7.1	*LRRC58*	lncRNA	protein_coding	1.31	0.39	tumors/normal
RP11-120I21.2	*LRRCC1*	lncRNA	protein_coding	1.01	0.82	HPV16+/HPV16-
CTC-526N19.1	*LSM14A*	lncRNA	protein_coding	-1.75	0.02	HPV16+/HPV16-
RP5-1043L13.1	*LSM14B*	lncRNA	protein_coding	-1.24	-0.17	HPV16+/HPV16-
RP1-102E24.8	*LTBR*	lncRNA	protein_coding	-1.04	-0.56	HPV16+/HPV16-
LA16c-OS12.2	*LUC7L*	lncRNA	protein_coding	1.37	0.17	HPV16+/HPV16-
RP11-109M17.2	*LURAP1L*	lncRNA	protein_coding	6.37	0.79	tumors/normal
RP1-80N2.3	*LY86*	lncRNA	protein_coding	1.52	-0.42	tumors/normal
RP4-580N22.2	*LYST*	lncRNA	protein_coding	-2.99	-0.67	HPV16+/HPV16-
RP11-291B21.2	*MAGOHB*	lncRNA	protein_coding	2.10	0.01	HPV16+/HPV16-
LINC00173	*MAP1LC3B2*	lncRNA	protein_coding	1.15	-0.67	HPV16+/HPV16-
RP1-193H18.2	*MAP2K6*	lncRNA	protein_coding	1.90	1.11	HPV16+/HPV16-
AF129075.5	*MAP3K7CL*	lncRNA	protein_coding	1.19	0.06	HPV16+/HPV16-
RP6-65G23.3	*MAP3K9*	lncRNA	protein_coding	-1.18	-0.38	HPV16+/HPV16-
RP3-462E2.5	*MAPKAPK5*	lncRNA	protein_coding	1.09	0.17	HPV16+/HPV16-

RP11-96A15.1	*MAPRE2*	lncRNA	protein_coding	-2.91	-0.83	HPV16+/HPV16-
RP11-505K9.5	*MBTPS1*	lncRNA	protein_coding	-1.24	-0.01	HPV16+/HPV16-
RP5-967N21.11	*MCM8*	lncRNA	protein_coding	1.41	0.79	HPV16+/HPV16-
RP11-57P19.1	*MCTP2*	lncRNA	protein_coding	1.03	-0.34	HPV16+/HPV16-
RP11-81H14.2	*MDM1*	lncRNA	protein_coding	1.62	1.34	HPV16+/HPV16-
RP11-20G13.1	*MEF2A*	lncRNA	protein_coding	-1.84	-0.24	HPV16+/HPV16-
CTC-501O10.1	*MEOX1*	lncRNA	protein_coding	1.06	0.13	HPV16+/HPV16-
RP11-571L19.8	*METAP1*	lncRNA	protein_coding	1.06	0.46	HPV16+/HPV16-
LA16c-380A1.1	*METRN*	lncRNA	protein_coding	-3.96	0.31	tumors/normal
AC144831.3	*METRNL*	lncRNA	protein_coding	-2.14	-0.81	tumors/normal
AC079767.4	*METTL21A*	lncRNA	protein_coding	2.43	-0.21	HPV16+/HPV16-
RP11-161I6.2	*METTL4*	lncRNA	protein_coding	3.55	0.44	tumors/normal
ZDHHC20-IT1	*MICU2*	lncRNA	protein_coding	1.18	0.21	HPV16+/HPV16-
RP5-1159O4.1	*MIOS*	lncRNA	protein_coding	-1.65	-0.72	tumors/normal
RP11-356O9.2	*MIPOL1*	lncRNA	protein_coding	4.80	0.79	tumors/normal
RP11-454K7.1	*MIS18BP1*	lncRNA	protein_coding	4.87	0.69	tumors/normal
RP11-4C20.4	*MKI67*	lncRNA	protein_coding	-3.68	0.54	HPV16+/HPV16-
AC112721.2	*MLPH*	lncRNA	protein_coding	4.72	-1.04	tumors/normal
AC156455.1	*MLXIP*	lncRNA	protein_coding	2.45	0.11	tumors/normal
RP11-451G4.2	*MME*	lncRNA	protein_coding	-4.48	0.32	tumors/normal
RP11-212I21.2	*MMP2*	lncRNA	protein_coding	1.87	-1.91	HPV16+/HPV16-
RP11-680H20.2	*MRE11A*	lncRNA	protein_coding	-3.41	0.07	HPV16+/HPV16-
RP11-230G5.2	*MSRB3*	lncRNA	protein_coding	-1.59	-1.55	HPV16+/HPV16-
CTD-2532K18.2	*MSX2*	lncRNA	protein_coding	5.97	-0.03	tumors/normal
RP11-16E12.1	*MTMR10*	lncRNA	protein_coding	1.00	0.01	HPV16+/HPV16-

RP11-932O9.10	*MTMR10*	lncRNA	protein_coding	1.83	0.01	HPV16+/HPV16-
AF131215.2	*MTMR9*	lncRNA	protein_coding	2.16	-0.05	HPV16+/HPV16-
LINC01139	*MTR*	lncRNA	protein_coding	2.04	0.14	HPV16+/HPV16-
RP11-806O11.1	*MTUS1*	lncRNA	protein_coding	2.22	0.83	HPV16+/HPV16-
HCG22	*MUC21*	lncRNA	protein_coding	-4.46	-6.95	tumors/normal
AC124944.5	*MUC4*	lncRNA	protein_coding	2.51	4.04	HPV16+/HPV16-
CASC21	*MYC*	lncRNA	protein_coding	-2.70	-0.87	HPV16+/HPV16-
LINC01405	*MYL2*	lncRNA	protein_coding	-2.48	-3.08	HPV16+/HPV16-
RP11-480G7.1	*MYLK3*	lncRNA	protein_coding	-5.38	-0.44	tumors/normal
RP11-1252I4.2	*N4BP3*	lncRNA	protein_coding	2.15	1.23	HPV16+/HPV16-
RP11-54A9.1	*NAP1L1*	lncRNA	protein_coding	-2.12	0.27	HPV16+/HPV16-
RP11-883A18.3	*NAPG*	lncRNA	protein_coding	1.40	0.12	tumors/normal
CH17-353B19.1	*NBPF12*	lncRNA	protein_coding	-1.00	0.41	HPV16+/HPV16-
RP11-89F3.2	*NBPF8*	lncRNA	protein_coding	2.13	0.16	HPV16+/HPV16-
RP4-791M13.3	*NBPF9*	lncRNA	protein_coding	1.49	0.35	HPV16+/HPV16-
RP11-572O6.1	*NCKIPSD*	lncRNA	protein_coding	1.75	0.45	HPV16+/HPV16-
RP11-701H24.7	*NDN*	lncRNA	protein_coding	-3.00	-1.73	tumors/normal
RP3-327A19.5	*NDUFA1*	lncRNA	protein_coding	1.10	0.24	HPV16+/HPV16-
TTLL11-IT1	*NDUFA8*	lncRNA	protein_coding	-3.65	0.12	HPV16+/HPV16-
RP4-660H19.1	*NEGR1*	lncRNA	protein_coding	-2.99	-0.34	HPV16+/HPV16-
LINC01315	*NFAM1*	lncRNA	protein_coding	2.78	1.03	HPV16+/HPV16-
CTA-126B4.7	*NFAM1*	lncRNA	protein_coding	3.20	1.03	HPV16+/HPV16-
AC003090.1	*NFE2L3*	lncRNA	protein_coding	-4.85	0.87	tumors/normal
RP11-776H12.1	*NFIA*	lncRNA	protein_coding	-2.64	0.75	HPV16+/HPV16-
RP4-580N22.2	*NID1*	lncRNA	protein_coding	-2.99	-1.95	HPV16+/HPV16-

RP3-327A19.5	*NKAP*	lncRNA	protein_coding	1.10	0.02	HPV16+/HPV16-
RP11-21B23.2	*NKD1*	lncRNA	protein_coding	-3.58	-2.13	HPV16+/HPV16-
RP11-129J12.2	*NKX2-3*	lncRNA	protein_coding	4.52	4.53	HPV16+/HPV16-
TTTY14	*NLGN4Y*	lncRNA	protein_coding	3.01	1.68	HPV16+/HPV16-
AC007249.3	*NOL10*	lncRNA	protein_coding	1.23	-0.29	HPV16+/HPV16-
CTD-2066L21.3	*NPR3*	lncRNA	protein_coding	-4.37	0.40	HPV16+/HPV16-
RP11-1100L3.8	*NR4A1*	lncRNA	protein_coding	-1.97	-2.11	tumors/normal
RP11-1002K11.1	*NRG1*	lncRNA	protein_coding	-2.92	-2.54	HPV16+/HPV16-
CASC15	*NRSN1*	lncRNA	protein_coding	-1.23	0.25	HPV16+/HPV16-
RP11-1038A11.3	*NTF3*	lncRNA	protein_coding	2.38	-2.99	tumors/normal
RP11-138J23.1	*NUDT12*	lncRNA	protein_coding	-1.56	-0.04	HPV16+/HPV16-
RP11-655M14.13	*NUDT8*	lncRNA	protein_coding	1.87	0.68	HPV16+/HPV16-
RP11-365O16.3	*NVL*	lncRNA	protein_coding	1.04	0.40	HPV16+/HPV16-
RP11-216P16.8	*OASL*	lncRNA	protein_coding	3.72	2.22	tumors/normal
RP11-541N10.3	*OBFC1*	lncRNA	protein_coding	-1.33	-1.05	tumors/normal
RP11-339B21.10	*ODF2*	lncRNA	protein_coding	-1.50	0.17	HPV16+/HPV16-
RP11-314C16.1	*OFCC1*	lncRNA	protein_coding	1.56	-2.11	HPV16+/HPV16-
RP13-20L14.1	*OGFOD3*	lncRNA	protein_coding	1.45	-0.33	HPV16+/HPV16-
LINC01305	*OLA1*	lncRNA	protein_coding	5.16	-0.11	HPV16+/HPV16-
RP11-407B7.1	*OPA1*	lncRNA	protein_coding	-3.22	0.08	tumors/normal
LHFPL3-AS1	*ORC5*	lncRNA	protein_coding	2.92	0.32	tumors/normal
RP11-480G7.1	*ORC6*	lncRNA	protein_coding	-5.38	1.72	tumors/normal
RP11-379B18.5	*OSBPL11*	lncRNA	protein_coding	1.10	-0.44	tumors/normal
RP11-44N12.5	*OSR2*	lncRNA	protein_coding	2.45	-0.24	HPV16+/HPV16-
CTD-2555K7.2	*OXA1L*	lncRNA	protein_coding	1.26	0.24	HPV16+/HPV16-

RP11-38P22.2	*P2RY1*	lncRNA	protein_coding	1.05	0.74	HPV16+/HPV16-
RP11-800A3.4	*P2RY2*	lncRNA	protein_coding	-1.73	-1.57	tumors/normal
RP11-800A3.4	*P2RY6*	lncRNA	protein_coding	-1.73	0.85	tumors/normal
LINC00491	*PAM*	lncRNA	protein_coding	3.89	0.22	tumors/normal
RP1-68D18.2	*PAMR1*	lncRNA	protein_coding	-1.74	-0.48	HPV16+/HPV16-
RP3-395M20.12	*PANK4*	lncRNA	protein_coding	-1.26	0.42	HPV16+/HPV16-
RP11-680H20.2	*PANX1*	lncRNA	protein_coding	-3.41	-1.55	HPV16+/HPV16-
AC139100.4	*PARD6G*	lncRNA	protein_coding	1.48	0.43	HPV16+/HPV16-
RP11-65J21.3	*PARN*	lncRNA	protein_coding	-2.72	-0.15	HPV16+/HPV16-
RP11-806O11.1	*PCM1*	lncRNA	protein_coding	2.22	0.72	HPV16+/HPV16-
XX-C2158C6.3	*PDCD2*	lncRNA	protein_coding	-2.90	-0.15	HPV16+/HPV16-
RP4-791M13.3	*PDE4DIP*	lncRNA	protein_coding	1.49	0.54	HPV16+/HPV16-
RP5-875H18.9	*PDK2*	lncRNA	protein_coding	-3.61	0.12	HPV16+/HPV16-
RP11-474O21.5	*PDPN*	lncRNA	protein_coding	2.07	-1.90	HPV16+/HPV16-
RP11-73G16.1	*PET112*	lncRNA	protein_coding	1.29	-0.85	tumors/normal
AP001059.7	*PFKL*	lncRNA	protein_coding	1.69	0.36	HPV16+/HPV16-
CTC-428G20.3	*PGGT1B*	lncRNA	protein_coding	-1.19	-0.64	tumors/normal
CTD-3131K8.2	*PGLS*	lncRNA	protein_coding	1.02	-0.08	HPV16+/HPV16-
RP11-420A23.1	*PGRMC2*	lncRNA	protein_coding	-1.36	-0.67	tumors/normal
RP11-883A18.3	*PIEZO2*	lncRNA	protein_coding	1.40	0.55	tumors/normal
KC6	*PIK3C3*	lncRNA	protein_coding	-1.94	0.25	HPV16+/HPV16-
RP11-434D9.2	*PIK3R1*	lncRNA	protein_coding	-1.11	0.34	HPV16+/HPV16-
RP11-77P16.4	*PIK3R4*	lncRNA	protein_coding	1.00	0.61	HPV16+/HPV16-
AP001046.5	*PKNOX1*	lncRNA	protein_coding	1.71	0.36	tumors/normal
RP11-373D23.3	*PLB1*	lncRNA	protein_coding	-1.50	-0.11	tumors/normal

RP11-81A22.5	*PLD2*	lncRNA	protein_coding	-1.75	-0.51	HPV16+/HPV16-
RP11-347C18.3	*PLEKHF2*	lncRNA	protein_coding	1.34	-0.03	HPV16+/HPV16-
CTD-2231H16.1	*PLEKHG4B*	lncRNA	protein_coding	-3.11	-1.81	HPV16+/HPV16-
RP11-798G7.8	*PLEKHM1*	lncRNA	protein_coding	1.24	-0.34	HPV16+/HPV16-
RP11-501O2.5	*PLSCR1*	lncRNA	protein_coding	2.36	0.79	tumors/normal
RP1-272L16.1	*PLXNA2*	lncRNA	protein_coding	-2.56	-0.16	HPV16+/HPV16-
AC004158.2	*PMFBP1*	lncRNA	protein_coding	1.94	0.17	tumors/normal
RP11-203M5.8	*PNP*	lncRNA	protein_coding	-1.01	-0.79	HPV16+/HPV16-
LINC00925	*POLG*	lncRNA	protein_coding	2.35	0.28	tumors/normal
CYP4F35P	*POTEC*	lncRNA	protein_coding	1.96	-0.42	HPV16+/HPV16-
PSORS1C3	*POU5F1*	lncRNA	protein_coding	1.60	1.07	HPV16+/HPV16-
CDC37L1-AS1	*PPAPDC2*	lncRNA	protein_coding	1.24	-0.03	HPV16+/HPV16-
ERVH-1	*PPARGC1A*	lncRNA	protein_coding	-2.04	-0.32	HPV16+/HPV16-
ACAP2-IT1	*PPP1R2*	lncRNA	protein_coding	1.15	0.20	HPV16+/HPV16-
RP11-657O9.1	*PPP2R3A*	lncRNA	protein_coding	-4.36	-0.35	HPV16+/HPV16-
RP11-15I11.3	*PPP2R5A*	lncRNA	protein_coding	-4.28	-0.29	HPV16+/HPV16-
RP11-248M19.1	*PQLC1*	lncRNA	protein_coding	1.15	0.01	HPV16+/HPV16-
RP11-430H10.3	*PRDM11*	lncRNA	protein_coding	-1.92	-0.36	HPV16+/HPV16-
CTA-520D8.2	*PRDM2*	lncRNA	protein_coding	-3.64	0.24	HPV16+/HPV16-
RP11-127B20.2	*PRDM8*	lncRNA	protein_coding	2.23	-0.08	HPV16+/HPV16-
RP5-884M6.1	*PRKAR2B*	lncRNA	protein_coding	-1.95	0.70	HPV16+/HPV16-
RP11-619A14.3	*PRKRIR*	lncRNA	protein_coding	-2.08	-0.01	HPV16+/HPV16-
RP11-81A22.5	*PSMB6*	lncRNA	protein_coding	-1.75	-0.13	HPV16+/HPV16-
RP11-387H17.6	*PSMD3*	lncRNA	protein_coding	1.90	0.36	tumors/normal
RP11-245J9.5	*PSMD6*	lncRNA	protein_coding	1.13	0.11	HPV16+/HPV16-

HCG22	*PSORS1C1*	lncRNA	protein_coding	-4.46	2.08	tumors/normal
RP11-498E2.9	*PTGS1*	lncRNA	protein_coding	-2.75	-2.04	HPV16+/HPV16-
RP11-973H7.1	*PTPN2*	lncRNA	protein_coding	1.55	0.43	tumors/normal
RP11-4C20.4	*PTPRE*	lncRNA	protein_coding	-3.68	-1.28	HPV16+/HPV16-
LINC00668	*PTPRM*	lncRNA	protein_coding	-1.44	-0.48	HPV16+/HPV16-
RP11-356J5.12	*PTS*	lncRNA	protein_coding	-1.21	-0.82	HPV16+/HPV16-
RP11-417L19.4	*PUS1*	lncRNA	protein_coding	-1.45	-0.23	HPV16+/HPV16-
RP11-80H18.4	*PXK*	lncRNA	protein_coding	1.90	0.40	HPV16+/HPV16-
RP11-422P24.11	*RAB13*	lncRNA	protein_coding	1.04	0.21	HPV16+/HPV16-
RP11-723O4.9	*RAB7A*	lncRNA	protein_coding	1.81	0.04	HPV16+/HPV16-
RP11-333E1.2	*RABEP1*	lncRNA	protein_coding	2.00	-0.35	HPV16+/HPV16-
RABGAP1L-IT1	*RABGAP1L*	lncRNA	protein_coding	1.19	0.44	tumors/normal
RP11-1038A11.3	*RAD51AP1*	lncRNA	protein_coding	2.38	1.69	tumors/normal
RP4-813D12.3	*RAE1*	lncRNA	protein_coding	1.08	-0.01	HPV16+/HPV16-
RAMP2-AS1	*RAMP2*	lncRNA	protein_coding	1.18	0.63	HPV16+/HPV16-
RP11-167P11.2	*RANBP10*	lncRNA	protein_coding	1.42	-0.37	HPV16+/HPV16-
LINC01160	*RAP1A*	lncRNA	protein_coding	3.40	-0.24	tumors/normal
CTD-2555C10.3	*RCOR1*	lncRNA	protein_coding	-2.91	-0.95	HPV16+/HPV16-
AC015849.16	*RDM1*	lncRNA	protein_coding	2.41	1.70	HPV16+/HPV16-
RP11-573D15.2	*RFC4*	lncRNA	protein_coding	3.05	1.40	tumors/normal
RP11-395N3.2	*RHBDD1*	lncRNA	protein_coding	-4.34	-0.20	HPV16+/HPV16-
LINC00925	*RHCG*	lncRNA	protein_coding	2.35	-2.32	tumors/normal
RP13-726E6.2	*RHOG*	lncRNA	protein_coding	1.54	-0.27	HPV16+/HPV16-
RP11-203M5.8	*RNASE10*	lncRNA	protein_coding	-1.01	-1.49	HPV16+/HPV16-
LINC01214	*RNF13*	lncRNA	protein_coding	-5.07	-0.02	HPV16+/HPV16-

AC092580.4	*RNF144A*	lncRNA	protein_coding	1.86	-0.30	HPV16+/HPV16-
RP11-54O7.18	*RNF223*	lncRNA	protein_coding	2.51	-1.31	tumors/normal
RP11-111M22.4	*RP11-111M22.2*	lncRNA	protein_coding	2.32	0.32	HPV16+/HPV16-
RP11-484N16.1	*RP11-169F17.1*	lncRNA	protein_coding	-2.81	-1.04	HPV16+/HPV16-
CTD-2354A18.1	*RP11-169F17.1*	lncRNA	protein_coding	-1.66	-1.04	HPV16+/HPV16-
RP11-484N16.1	*RP11-41O4.1*	lncRNA	protein_coding	-2.81	0.17	HPV16+/HPV16-
RP4-758J18.13	*RP4-758J18.2*	lncRNA	protein_coding	1.23	0.04	tumors/normal
RP11-493L12.5	*RPAP3*	lncRNA	protein_coding	3.45	0.07	tumors/normal
AC108004.2	*RPH3AL*	lncRNA	protein_coding	4.00	0.60	HPV16+/HPV16-
RP11-391M1.4	*RPL14*	lncRNA	protein_coding	-1.64	-0.87	tumors/normal
RP11-80H18.4	*RPP14*	lncRNA	protein_coding	1.90	0.45	HPV16+/HPV16-
RP11-414C23.1	*RPS4Y1*	lncRNA	protein_coding	2.05	1.85	HPV16+/HPV16-
CTD-3035D6.2	*RPS6KA5*	lncRNA	protein_coding	3.31	-0.16	tumors/normal
RP11-567G11.1	*RTP4*	lncRNA	protein_coding	-4.20	-0.32	HPV16+/HPV16-
RP11-376M2.2	*RUNDC1*	lncRNA	protein_coding	1.11	-0.11	tumors/normal
RP11-100L22.3	*RUNX1T1*	lncRNA	protein_coding	2.92	-0.48	HPV16+/HPV16-
RP11-200A1.1	*RYK*	lncRNA	protein_coding	3.11	0.06	HPV16+/HPV16-
CTC-429L19.3	*S1PR5*	lncRNA	protein_coding	2.46	0.91	HPV16+/HPV16-
RP11-361F15.2	*SASH1*	lncRNA	protein_coding	-1.17	-2.51	tumors/normal
LINC00263	*SCD*	lncRNA	protein_coding	-1.02	-0.08	HPV16+/HPV16-
RP11-333E1.2	*SCIMP*	lncRNA	protein_coding	2.00	0.71	HPV16+/HPV16-
AC011891.5	*SCIN*	lncRNA	protein_coding	-4.04	-1.93	tumors/normal
KB-1440D3.13	*SDF2L1*	lncRNA	protein_coding	3.63	-0.46	HPV16+/HPV16-
RP11-140I16.3	*SDR16C5*	lncRNA	protein_coding	1.70	0.04	HPV16+/HPV16-
RP13-20L14.1	*SECTM1*	lncRNA	protein_coding	1.45	0.18	HPV16+/HPV16-

SNHG18	*SEMA5A*	lncRNA	protein_coding	-1.77	-1.33	tumors/normal
RP11-285E9.6	*SEPT9*	lncRNA	protein_coding	-1.10	-0.49	HPV16+/HPV16-
RP11-635N19.3	*SERPINB5*	lncRNA	protein_coding	-2.00	-1.17	HPV16+/HPV16-
RP11-338H14.1	*SESN3*	lncRNA	protein_coding	2.88	0.28	tumors/normal
LINC00707	*SFMBT2*	lncRNA	protein_coding	-1.94	0.03	HPV16+/HPV16-
CTD-2532K18.2	*SFXN1*	lncRNA	protein_coding	5.97	0.67	tumors/normal
RP11-108L7.15	*SFXN3*	lncRNA	protein_coding	1.10	-1.18	HPV16+/HPV16-
RP11-557H15.3	*SGK1*	lncRNA	protein_coding	-4.49	0.41	HPV16+/HPV16-
CTD-2302E22.2	*SGPP1*	lncRNA	protein_coding	-1.66	0.49	tumors/normal
AC010148.1	*SH3BP4*	lncRNA	protein_coding	1.79	-0.07	tumors/normal
LINC00273	*SHCBP1*	lncRNA	protein_coding	2.76	1.44	tumors/normal
LINC00675	*SHISA6*	lncRNA	protein_coding	-4.38	-3.82	tumors/normal
AC107072.2	*SHROOM3*	lncRNA	protein_coding	-5.84	-3.01	tumors/normal
RP6-114E22.1	*SIPA1L1*	lncRNA	protein_coding	-2.53	-0.09	HPV16+/HPV16-
CTD-2554C21.2	*SIPA1L3*	lncRNA	protein_coding	-3.11	0.57	tumors/normal
RP5-968J1.1	*SIRPG*	lncRNA	protein_coding	-3.18	1.86	HPV16+/HPV16-
AC007386.2	*SLC1A4*	lncRNA	protein_coding	1.18	0.68	tumors/normal
AP006621.6	*SLC25A22*	lncRNA	protein_coding	1.40	-0.32	HPV16+/HPV16-
RP11-119J18.1	*SLC25A48*	lncRNA	protein_coding	-4.27	-2.08	HPV16+/HPV16-
RP11-323C15.2	*SLC2A10*	lncRNA	protein_coding	4.81	-0.56	HPV16+/HPV16-
LINC01279	*SLC35A5*	lncRNA	protein_coding	-1.62	-0.08	tumors/normal
RP11-314C16.1	*SLC35B3*	lncRNA	protein_coding	1.56	-0.04	HPV16+/HPV16-
RP11-38M8.1	*SLC35B4*	lncRNA	protein_coding	2.56	0.18	tumors/normal
CTD-3010D24.3	*SLC39A11*	lncRNA	protein_coding	2.06	0.38	tumors/normal
LINC00885	*SLC51A*	lncRNA	protein_coding	1.88	0.63	HPV16+/HPV16-

RP11-80H8.4	*SLC9A9*	lncRNA	protein_coding	3.38	-0.66	tumors/normal
PKI55	*SMARCAL1*	lncRNA	protein_coding	-1.52	-0.03	tumors/normal
RP11-497H16.9	*SMN2*	lncRNA	protein_coding	1.46	-0.09	HPV16+/HPV16-
RP11-525A16.4	*SMNDC1*	lncRNA	protein_coding	-2.17	-0.28	HPV16+/HPV16-
RP11-417E7.1	*SMOC2*	lncRNA	protein_coding	-4.08	-0.22	HPV16+/HPV16-
AC019117.2	*SNX13*	lncRNA	protein_coding	-2.68	-0.54	HPV16+/HPV16-
RP11-120I21.2	*SNX16*	lncRNA	protein_coding	1.01	-0.14	HPV16+/HPV16-
RP11-890B15.2	*SNX19*	lncRNA	protein_coding	-1.09	-0.91	HPV16+/HPV16-
AP000253.1	*SOD1*	lncRNA	protein_coding	1.20	-0.20	tumors/normal
RP11-820L6.1	*SORL1*	lncRNA	protein_coding	-1.73	-0.59	HPV16+/HPV16-
GS1-166A23.1	*SOSTDC1*	lncRNA	protein_coding	-2.47	-0.94	HPV16+/HPV16-
AC107072.2	*SOWAHB*	lncRNA	protein_coding	-5.84	-1.77	tumors/normal
SOX21-AS1	*SOX21*	lncRNA	protein_coding	1.40	1.82	HPV16+/HPV16-
CASC15	*SOX4*	lncRNA	protein_coding	-1.23	0.39	HPV16+/HPV16-
RP11-153K16.1	*SOX5*	lncRNA	protein_coding	1.96	-0.87	HPV16+/HPV16-
RP11-796E10.1	*SP3*	lncRNA	protein_coding	3.08	0.10	tumors/normal
RP11-700H6.4	*SPAG9*	lncRNA	protein_coding	-2.78	0.54	tumors/normal
AC004951.6	*SPDYE1*	lncRNA	protein_coding	1.15	0.83	HPV16+/HPV16-
RP11-10O22.1	*SPTSSB*	lncRNA	protein_coding	1.20	-3.23	tumors/normal
SPTY2D1-AS1	*SPTY2D1*	lncRNA	protein_coding	-1.05	-0.28	HPV16+/HPV16-
CTA-250D10.23	*SREBF2*	lncRNA	protein_coding	2.30	0.48	HPV16+/HPV16-
RP11-546K22.3	*ST18*	lncRNA	protein_coding	1.03	0.10	HPV16+/HPV16-
CTD-2340E1.2	*ST8SIA4*	lncRNA	protein_coding	1.04	-0.11	tumors/normal
RP1-315G1.3	*STAG2*	lncRNA	protein_coding	1.75	0.35	HPV16+/HPV16-
CTB-33O18.1	*STC2*	lncRNA	protein_coding	-4.17	-1.37	HPV16+/HPV16-

RP11-44N12.5	*STK3*	lncRNA	protein_coding	2.45	-0.55	HPV16+/HPV16-
RP11-128M1.1	*STK35*	lncRNA	protein_coding	-2.09	0.18	tumors/normal
RP11-361F15.2	*STXBP5*	lncRNA	protein_coding	-1.17	-0.72	tumors/normal
RP11-131L12.3	*SUDS3*	lncRNA	protein_coding	1.04	0.30	HPV16+/HPV16-
RP11-239E10.3	*SUSD4*	lncRNA	protein_coding	-3.33	-1.78	tumors/normal
RP11-410K21.2	*SVEP1*	lncRNA	protein_coding	1.15	-0.42	HPV16+/HPV16-
RP4-568C11.4	*SYNDIG1*	lncRNA	protein_coding	1.28	-0.45	HPV16+/HPV16-
RP11-7K24.3	*TAF8*	lncRNA	protein_coding	-1.77	0.16	tumors/normal
RP11-191L9.4	*TBC1D22A*	lncRNA	protein_coding	4.00	0.29	tumors/normal
RP11-388C12.8	*TBCD*	lncRNA	protein_coding	1.54	0.01	tumors/normal
RP5-1057J7.7	*TCEA3*	lncRNA	protein_coding	1.75	2.13	HPV16+/HPV16-
LINC00926	*TCF12*	lncRNA	protein_coding	1.06	-0.10	HPV16+/HPV16-
AC005256.1	*TCF3*	lncRNA	protein_coding	-3.16	0.20	HPV16+/HPV16-
RP11-397A16.1	*TCF4*	lncRNA	protein_coding	4.70	0.35	tumors/normal
RP11-91J19.4	*TDRP*	lncRNA	protein_coding	1.64	-0.69	HPV16+/HPV16-
RP11-372B4.3	*TEAD4*	lncRNA	protein_coding	1.39	-0.61	HPV16+/HPV16-
RP11-802F5.1	*TENM4*	lncRNA	protein_coding	6.29	0.64	tumors/normal
RP11-128M1.1	*TGM3*	lncRNA	protein_coding	-2.09	-5.72	tumors/normal
RP5-1097P24.1	*THBD*	lncRNA	protein_coding	3.03	-0.70	HPV16+/HPV16-
RP11-73K9.3	*TIFA*	lncRNA	protein_coding	1.70	1.20	HPV16+/HPV16-
RP11-553L6.5	*TIGIT*	lncRNA	protein_coding	-1.40	1.96	tumors/normal
RP11-279F6.3	*TLE3*	lncRNA	protein_coding	1.36	0.15	HPV16+/HPV16-
RP11-239E10.2	*TLR5*	lncRNA	protein_coding	3.07	1.14	HPV16+/HPV16-
RP11-1277A3.3	*TMED9*	lncRNA	protein_coding	1.14	-0.20	HPV16+/HPV16-
RP3-462E2.5	*TMEM116*	lncRNA	protein_coding	1.09	0.74	HPV16+/HPV16-

RP11-529E10.7	*TMEM128*	lncRNA	protein_coding	1.32	0.10	HPV16+/HPV16-
RP11-177J6.1	*TMEM165*	lncRNA	protein_coding	1.56	-0.01	HPV16+/HPV16-
RP11-60A8.1	*TMEM171*	lncRNA	protein_coding	-2.40	0.23	tumors/normal
RP5-933K21.3	*TMEM242*	lncRNA	protein_coding	-1.15	-0.51	HPV16+/HPV16-
RP11-218E20.3	*TMX1*	lncRNA	protein_coding	2.36	0.56	tumors/normal
LINC00519	*TMX1*	lncRNA	protein_coding	2.86	0.56	tumors/normal
RP11-108K3.1	*TNFAIP8L3*	lncRNA	protein_coding	2.44	-1.29	tumors/normal
RP3-395M20.8	*TNFRSF14*	lncRNA	protein_coding	1.14	0.86	HPV16+/HPV16-
AC124944.5	*TNK2*	lncRNA	protein_coding	2.51	0.05	HPV16+/HPV16-
RP11-389C8.3	*TNPO1*	lncRNA	protein_coding	1.11	-0.37	HPV16+/HPV16-
AC095067.1	*TNS3*	lncRNA	protein_coding	1.69	0.91	tumors/normal
RP5-1028K7.2	*TNS4*	lncRNA	protein_coding	2.58	1.21	tumors/normal
AC005682.5	*TOMM7*	lncRNA	protein_coding	-1.75	-0.26	HPV16+/HPV16-
RP11-347C18.3	*TP53INP1*	lncRNA	protein_coding	1.34	0.47	HPV16+/HPV16-
SSTR5-AS1	*TPSB2*	lncRNA	protein_coding	1.47	0.15	HPV16+/HPV16-
RP11-129K12.3	*TRANK1*	lncRNA	protein_coding	1.87	0.21	HPV16+/HPV16-
RP11-831A10.1	*TRIM29*	lncRNA	protein_coding	2.02	-0.35	HPV16+/HPV16-
CTC-428G20.3	*TRIM36*	lncRNA	protein_coding	-1.19	0.95	tumors/normal
RP11-100L22.3	*TRIQK*	lncRNA	protein_coding	2.92	-0.16	HPV16+/HPV16-
RP11-304L19.1	*TSC2*	lncRNA	protein_coding	1.86	0.63	HPV16+/HPV16-
LINC01214	*TSC22D2*	lncRNA	protein_coding	-5.07	-1.11	HPV16+/HPV16-
LINC00941	*TSPAN11*	lncRNA	protein_coding	-1.66	-0.74	HPV16+/HPV16-
RP1-93H18.7	*TSPYL1*	lncRNA	protein_coding	-1.45	-0.17	HPV16+/HPV16-
BCYRN1	*TTC7A*	lncRNA	protein_coding	-1.04	-0.13	HPV16+/HPV16-
CTD-3035D6.2	*TTC7B*	lncRNA	protein_coding	3.31	0.32	tumors/normal

RP11-465B22.8	*TTLL10*	lncRNA	protein_coding	1.78	0.54	HPV16+/HPV16-
TTLL11-IT1	*TTLL11*	lncRNA	protein_coding	-3.65	-0.92	HPV16+/HPV16-
RP11-357H14.17	*TTLL6*	lncRNA	protein_coding	5.71	1.16	tumors/normal
RP11-475O6.1	*TTLL7*	lncRNA	protein_coding	-1.18	0.23	tumors/normal
RP11-64C12.6	*TUBB6*	lncRNA	protein_coding	4.13	-1.16	HPV16+/HPV16-
AC005083.1	*TWISTNB*	lncRNA	protein_coding	1.61	-0.29	HPV16+/HPV16-
LINC-ROR	*TXNL1*	lncRNA	protein_coding	-1.31	0.43	HPV16+/HPV16-
AC073043.1	*TYW5*	lncRNA	protein_coding	1.10	0.12	HPV16+/HPV16-
AC104820.2	*UBE2E3*	lncRNA	protein_coding	3.00	-0.07	HPV16+/HPV16-
RP11-701H24.3	*UBE3A*	lncRNA	protein_coding	-3.34	-0.72	tumors/normal
CTB-11I22.2	*UBLCP1*	lncRNA	protein_coding	2.59	-0.03	tumors/normal
SPTY2D1-AS1	*UEVLD*	lncRNA	protein_coding	-1.05	0.03	HPV16+/HPV16-
RP11-417L19.4	*ULK1*	lncRNA	protein_coding	-1.45	0.02	HPV16+/HPV16-
RP11-71H17.9	*UMPS*	lncRNA	protein_coding	-1.32	0.26	HPV16+/HPV16-
RP11-351J23.1	*UNC93A*	lncRNA	protein_coding	-5.59	-0.94	tumors/normal
RP5-903G2.2	*UNC93B1*	lncRNA	protein_coding	1.99	0.25	HPV16+/HPV16-
LINC00478	*USP25*	lncRNA	protein_coding	1.27	-0.07	HPV16+/HPV16-
RP11-338N10.3	*VAMP3*	lncRNA	protein_coding	1.87	-0.13	HPV16+/HPV16-
AC068831.16	*VPS33B*	lncRNA	protein_coding	-1.18	-0.04	HPV16+/HPV16-
RP3-460G2.2	*VTA1*	lncRNA	protein_coding	-1.71	-0.36	HPV16+/HPV16-
RP11-762H8.4	*WDR61*	lncRNA	protein_coding	-2.01	-0.34	tumors/normal
CTD-2302E22.2	*WDR89*	lncRNA	protein_coding	-1.66	0.21	tumors/normal
RP11-619A14.3	*WNT11*	lncRNA	protein_coding	-2.08	-2.56	HPV16+/HPV16-
ESRG	*WNT5A*	lncRNA	protein_coding	-1.92	-1.22	HPV16+/HPV16-
AF131215.9	*XKR6*	lncRNA	protein_coding	1.13	1.66	HPV16+/HPV16-

RP11-23B15.1	*XPA*	lncRNA	protein_coding	1.80	0.38	HPV16+/HPV16-
PKI55	*XRCC5*	lncRNA	protein_coding	-1.52	-0.26	tumors/normal
AC093850.2	*XRCC5*	lncRNA	protein_coding	6.29	-0.26	tumors/normal
RP11-620J15.3	*XRCC6BP1*	lncRNA	protein_coding	1.04	0.29	HPV16+/HPV16-
RP5-1097P24.1	*XRN2*	lncRNA	protein_coding	3.03	0.07	HPV16+/HPV16-
KB-1440D3.13	*YPEL1*	lncRNA	protein_coding	3.63	0.77	HPV16+/HPV16-
CTD-2555O16.3	*ZBTB25*	lncRNA	protein_coding	2.38	0.05	HPV16+/HPV16-
RP5-933K21.3	*ZDHHC14*	lncRNA	protein_coding	-1.15	-0.12	HPV16+/HPV16-
RP11-242F4.2	*ZDHHC2*	lncRNA	protein_coding	1.21	0.52	HPV16+/HPV16-
LINC00539	*ZDHHC20*	lncRNA	protein_coding	1.17	0.01	HPV16+/HPV16-
ZDHHC20-IT1	*ZDHHC20*	lncRNA	protein_coding	1.18	0.01	HPV16+/HPV16-
TEX41	*ZEB2*	lncRNA	protein_coding	-1.08	-0.37	HPV16+/HPV16-
RP11-863P13.3	*ZFPM1*	lncRNA	protein_coding	1.82	0.38	tumors/normal
RP11-414C23.1	*ZFY*	lncRNA	protein_coding	2.05	1.85	HPV16+/HPV16-
RP11-44N11.1	*ZHX2*	lncRNA	protein_coding	2.37	-0.29	tumors/normal
RP11-323C15.2	*ZMYND8*	lncRNA	protein_coding	4.81	-0.17	HPV16+/HPV16-
RP11-571E6.3	*ZNF157*	lncRNA	protein_coding	1.09	0.05	tumors/normal
AC069278.4	*ZNF180*	lncRNA	protein_coding	1.15	0.63	HPV16+/HPV16-
AC069278.4	*ZNF235*	lncRNA	protein_coding	1.15	0.35	HPV16+/HPV16-
CTC-559E9.5	*ZNF253*	lncRNA	protein_coding	-1.16	-0.69	tumors/normal
AC003005.2	*ZNF304*	lncRNA	protein_coding	1.71	-0.64	tumors/normal
RP11-457M11.5	*ZNF322*	lncRNA	protein_coding	1.92	0.23	HPV16+/HPV16-
RP11-42I10.1	*ZNF423*	lncRNA	protein_coding	-1.14	-0.74	HPV16+/HPV16-
AC074138.3	*ZNF461*	lncRNA	protein_coding	1.36	0.41	HPV16+/HPV16-
ZNF667-AS1	*ZNF471*	lncRNA	protein_coding	1.91	0.65	HPV16+/HPV16-

AC003005.2	*ZNF549*	lncRNA	protein_coding	1.71	0.09	tumors/normal
AC074138.3	*ZNF567*	lncRNA	protein_coding	1.36	0.29	HPV16+/HPV16-
CTD-2554C21.2	*ZNF573*	lncRNA	protein_coding	-3.11	-0.01	tumors/normal
CTD-2619J13.13	*ZNF584*	lncRNA	protein_coding	-2.26	-0.13	tumors/normal
CTD-2619J13.14	*ZNF584*	lncRNA	protein_coding	2.49	-0.13	tumors/normal
RP11-391M1.4	*ZNF619*	lncRNA	protein_coding	-1.64	0.04	tumors/normal
RP4-665J23.4	*ZNF644*	lncRNA	protein_coding	1.44	0.15	tumors/normal
ZNF667-AS1	*ZNF667*	lncRNA	protein_coding	1.91	0.98	HPV16+/HPV16-
CTD-2245F17.9	*ZNF677*	lncRNA	protein_coding	3.02	-0.36	tumors/normal
CTD-2006C1.12	*ZNF69*	lncRNA	protein_coding	1.15	-0.17	HPV16+/HPV16-
KB-1410C5.5	*ZNF706*	lncRNA	protein_coding	-1.06	-0.39	HPV16+/HPV16-
RP11-1136G11.8	*ZNF740*	lncRNA	protein_coding	1.77	0.56	HPV16+/HPV16-
AC005276.1	*ZNF800*	lncRNA	protein_coding	-1.78	-0.41	HPV16+/HPV16-
CTD-2245F17.9	*ZNF845*	lncRNA	protein_coding	3.02	-0.29	tumors/normal
CTC-436P18.3	*ZSWIM6*	lncRNA	protein_coding	1.01	0.31	HPV16+/HPV16-
ABCA17P	*ABCA3*	pseudogene	protein_coding	4.99	2.18	HPV16+/HPV16-
RP4-800M22.1	*ADH5*	pseudogene	protein_coding	-1.17	0.49	HPV16+/HPV16-
TSSC2	*ALG1L2*	pseudogene	protein_coding	2.41	1.01	tumors/normal
TCAM1P	*ANKRD9*	pseudogene	protein_coding	2.07	0.06	tumors/normal
CYP2AB1P	*APOL4*	pseudogene	protein_coding	-1.24	0.31	HPV16+/HPV16-
ATP5G1P4	*ATP5G1*	pseudogene	protein_coding	3.77	-0.46	tumors/normal
BMS1P10	*BMS1*	pseudogene	protein_coding	-2.36	-0.18	tumors/normal
BMS1P8	*BMS1*	pseudogene	protein_coding	-2.25	-0.18	tumors/normal
RP11-583F2.1	*BPTF*	pseudogene	protein_coding	1.21	-0.11	tumors/normal
RP11-564D11.3	*C10orf88*	pseudogene	protein_coding	2.39	0.09	tumors/normal

SLC22A20	*C15orf40*	pseudogene	protein_coding	3.04	1.02	tumors/normal
CTD-2014N11.1	*C15orf43*	pseudogene	protein_coding	2.28	0.20	HPV16+/HPV16-
RP11-583F2.1	*C2CD3*	pseudogene	protein_coding	1.21	2.15	tumors/normal
TSSC2	*C2CD3*	pseudogene	protein_coding	2.41	2.15	tumors/normal
RP11-154P18.2	*C2orf27A*	pseudogene	protein_coding	2.34	0.16	HPV16+/HPV16-
CTD-2124B8.2	*C2orf49*	pseudogene	protein_coding	1.07	-0.47	HPV16+/HPV16-
RP11-578O24.2	*C4orf46*	pseudogene	protein_coding	1.54	0.68	HPV16+/HPV16-
KRT42P	*CAMKK1*	pseudogene	protein_coding	3.87	-0.02	HPV16+/HPV16-
CASP1P2	*CASP1*	pseudogene	protein_coding	-1.71	-1.07	HPV16+/HPV16-
CD8BP	*CD8B*	pseudogene	protein_coding	5.12	3.55	HPV16+/HPV16-
ABCA17P	*CEP41*	pseudogene	protein_coding	4.99	-0.41	HPV16+/HPV16-
AC008072.1	*CHML*	pseudogene	protein_coding	1.38	0.16	tumors/normal
COX5BP6	*COX5B*	pseudogene	protein_coding	2.58	0.36	HPV16+/HPV16-
CROCCP4	*CROCC*	pseudogene	protein_coding	2.79	0.96	tumors/normal
CYCSP6	*CYCS*	pseudogene	protein_coding	-5.06	-0.27	HPV16+/HPV16-
RP11-22B23.1	*DDX11*	pseudogene	protein_coding	1.27	1.66	tumors/normal
DDX12P	*DDX11*	pseudogene	protein_coding	1.73	1.66	tumors/normal
EIF4HP2	*EIF4H*	pseudogene	protein_coding	1.16	-0.11	HPV16+/HPV16-
ESPNP	*ESPN*	pseudogene	protein_coding	1.02	0.74	HPV16+/HPV16-
FABP5P1	*FABP5*	pseudogene	protein_coding	-2.33	-1.87	HPV16+/HPV16-
FAM27E3	*FAM182B*	pseudogene	protein_coding	1.03	1.86	HPV16+/HPV16-
WTAPP1	*FAM227B*	pseudogene	protein_coding	-2.13	-0.15	HPV16+/HPV16-
RP11-12A20.6	*FAM27C*	pseudogene	protein_coding	1.05	0.79	HPV16+/HPV16-
RP11-157L3.10	*FAM27C*	pseudogene	protein_coding	2.41	0.79	HPV16+/HPV16-
FAM86HP	*FAM86A*	pseudogene	protein_coding	1.05	0.13	HPV16+/HPV16-

FAM86HP	*FAM86B1*	pseudogene	protein_coding	1.05	0.86	HPV16+/HPV16-
FAM96AP2	*FAM96A*	pseudogene	protein_coding	-2.83	0.17	HPV16+/HPV16-
LINC01296	*FAM98B*	pseudogene	protein_coding	2.42	-0.12	tumors/normal
DUXAP10	*FAM98B*	pseudogene	protein_coding	2.49	-0.12	tumors/normal
FCF1P2	*FCF1*	pseudogene	protein_coding	1.13	-0.45	HPV16+/HPV16-
AC114737.3	*FDPS*	pseudogene	protein_coding	1.87	0.42	HPV16+/HPV16-
AC006014.8	*FKBP6*	pseudogene	protein_coding	3.60	3.04	HPV16+/HPV16-
RP11-159J3.1	*GAPDH*	pseudogene	protein_coding	-1.85	0.08	HPV16+/HPV16-
RP11-52B19.10	*GCNT3*	pseudogene	protein_coding	1.15	0.10	HPV16+/HPV16-
RP11-490K7.4	*GTF2A2*	pseudogene	protein_coding	1.69	0.04	HPV16+/HPV16-
GUSBP2	*GUSB*	pseudogene	protein_coding	1.07	0.74	HPV16+/HPV16-
RP11-1023L17.1	*GUSB*	pseudogene	protein_coding	1.14	0.74	HPV16+/HPV16-
GUSBP3	*GUSB*	pseudogene	protein_coding	1.22	-0.06	tumors/normal
GUSBP4	*GUSB*	pseudogene	protein_coding	2.28	0.74	HPV16+/HPV16-
RP11-4M23.4	*H3F3A*	pseudogene	protein_coding	1.83	0.96	HPV16+/HPV16-
HERC2P2	*HERC2*	pseudogene	protein_coding	1.27	0.42	HPV16+/HPV16-
RPL12P38	*HMGN2*	pseudogene	protein_coding	1.05	1.34	HPV16+/HPV16-
HMGN2P8	*HMGN2*	pseudogene	protein_coding	1.23	1.34	HPV16+/HPV16-
HMGN2P17	*HMGN2*	pseudogene	protein_coding	1.27	1.34	HPV16+/HPV16-
HMGN2P18	*HMGN2*	pseudogene	protein_coding	2.22	1.34	HPV16+/HPV16-
HMGN2P15	*HMGN2*	pseudogene	protein_coding	4.30	1.34	HPV16+/HPV16-
HNRNPA1P21	*HNRNPA1*	pseudogene	protein_coding	2.03	0.09	tumors/normal
RP11-416L21.2	*IFITM3*	pseudogene	protein_coding	-2.83	-0.87	HPV16+/HPV16-
IFITM9P	*IFITM3*	pseudogene	protein_coding	-1.87	-0.87	HPV16+/HPV16-
FTLP10	*IFNLR1*	pseudogene	protein_coding	1.39	-0.87	HPV16+/HPV16-

TMPRSS11BNL	*IFNLR1*	pseudogene	protein_coding	2.71	-0.87	HPV16+/HPV16-
RP11-529F4.1	*IFT122*	pseudogene	protein_coding	1.42	0.46	HPV16+/HPV16-
ISCA1P4	*ISCA1*	pseudogene	protein_coding	2.38	-0.16	HPV16+/HPV16-
RP11-480I12.5	*KIAA1683*	pseudogene	protein_coding	1.37	0.41	HPV16+/HPV16-
RP11-213G2.3	*KIF27*	pseudogene	protein_coding	1.34	0.84	HPV16+/HPV16-
MAGOH2	*MAGOH*	pseudogene	protein_coding	1.52	0.31	HPV16+/HPV16-
RP11-551L14.1	*MAN2A2*	pseudogene	protein_coding	2.80	-0.70	tumors/normal
WTAPP1	*MMP1*	pseudogene	protein_coding	-2.13	-4.33	HPV16+/HPV16-
RP11-632K20.2	*MPHOSPH10*	pseudogene	protein_coding	1.48	-0.12	HPV16+/HPV16-
RP11-83M16.3	*MRPL39*	pseudogene	protein_coding	-2.68	0.30	HPV16+/HPV16-
MT1L	*MT1E*	pseudogene	protein_coding	-1.53	-2.41	HPV16+/HPV16-
MT1JP	*MT1M*	pseudogene	protein_coding	-2.14	-0.16	HPV16+/HPV16-
MTX1P1	*MTX1*	pseudogene	protein_coding	1.38	1.10	tumors/normal
NBEAP3	*NBEA*	pseudogene	protein_coding	3.24	-2.01	tumors/normal
NPM1P25	*NPM1*	pseudogene	protein_coding	1.46	0.25	HPV16+/HPV16-
NUDT16P1	*NUDT16*	pseudogene	protein_coding	1.04	0.25	HPV16+/HPV16-
RP11-210K20.3	*PAK4*	pseudogene	protein_coding	2.48	-0.12	tumors/normal
RP11-64B16.2	*PDAP1*	pseudogene	protein_coding	-3.67	0.35	tumors/normal
WI2-1896O14.1	*PDE4DIP*	pseudogene	protein_coding	-3.65	-1.80	tumors/normal
ESPNP	*PDE4DIP*	pseudogene	protein_coding	1.02	0.54	HPV16+/HPV16-
C6orf183	*PHACTR4*	pseudogene	protein_coding	-1.28	0.42	HPV16+/HPV16-
POLR2KP1	*POLR2K*	pseudogene	protein_coding	1.82	0.11	HPV16+/HPV16-
PPIAP26	*PPIA*	pseudogene	protein_coding	-2.32	-0.03	HPV16+/HPV16-
AC005517.3	*PPIA*	pseudogene	protein_coding	1.17	-0.03	HPV16+/HPV16-
PPP1R11P1	*PPP1R11*	pseudogene	protein_coding	3.35	-2.37	tumors/normal

XXbac-B444P24.10	*PRODH*	pseudogene	protein_coding	1.33	1.44	HPV16+/HPV16-
RP11-430K21.2	*PRRC2A*	pseudogene	protein_coding	3.37	0.45	tumors/normal
PSPC1P1	*PSPC1*	pseudogene	protein_coding	-2.71	0.82	HPV16+/HPV16-
RP11-10O17.1	*RILPL2*	pseudogene	protein_coding	1.11	0.10	tumors/normal
RP11-677M14.5	*RNF181*	pseudogene	protein_coding	1.08	0.01	HPV16+/HPV16-
RP11-251G23.2	*RNF181*	pseudogene	protein_coding	1.08	0.01	HPV16+/HPV16-
RP11-834C11.7	*RP11-160N1.10*	pseudogene	protein_coding	3.10	2.50	tumors/normal
IGHV3OR16-7	*RP11-812E19.9*	pseudogene	protein_coding	2.11	3.04	HPV16+/HPV16-
IGHV3OR16-15	*RP11-812E19.9*	pseudogene	protein_coding	2.82	3.04	HPV16+/HPV16-
RP11-1166P10.8	*RP11-812E19.9*	pseudogene	protein_coding	3.25	3.04	HPV16+/HPV16-
IGHV3-71	*RP11-812E19.9*	pseudogene	protein_coding	3.35	3.04	HPV16+/HPV16-
AC010677.5	*RPL23*	pseudogene	protein_coding	-4.87	-0.15	HPV16+/HPV16-
RPL29P19	*RPL29*	pseudogene	protein_coding	4.62	-0.97	tumors/normal
RP11-70F11.2	*RPL35A*	pseudogene	protein_coding	4.57	0.02	tumors/normal
RPL37P2	*RPL37*	pseudogene	protein_coding	-2.61	-0.07	HPV16+/HPV16-
RP11-17A4.1	*RPL37*	pseudogene	protein_coding	-1.94	-0.07	tumors/normal
RP11-54C4.1	*RPLP1*	pseudogene	protein_coding	1.32	0.28	HPV16+/HPV16-
RP1-89D4.1	*RPS24*	pseudogene	protein_coding	2.17	0.36	HPV16+/HPV16-
RPS27P23	*RPS27*	pseudogene	protein_coding	-2.97	-0.45	tumors/normal
RP11-10G12.1	*RPS27*	pseudogene	protein_coding	-2.34	-0.45	tumors/normal
RP11-51O6.1	*RPS27A*	pseudogene	protein_coding	-1.79	-0.25	tumors/normal
SDHAP1	*SDHA*	pseudogene	protein_coding	1.03	0.35	HPV16+/HPV16-
SMG1P7	*SEC11A*	pseudogene	protein_coding	3.26	0.17	tumors/normal
RP11-1277A3.2	*SIMC1*	pseudogene	protein_coding	2.28	1.03	HPV16+/HPV16-
AC027612.6	*SLC2A9*	pseudogene	protein_coding	-1.43	-1.36	HPV16+/HPV16-

SLC9A3P2	*SLC9A3*	pseudogene	protein_coding	4.78	-0.20	tumors/normal
RP11-583F2.1	*SMAD2*	pseudogene	protein_coding	1.21	-0.27	tumors/normal
SMG1P7	*SMG1*	pseudogene	protein_coding	3.26	0.43	tumors/normal
SMPD4P1	*SMPD4*	pseudogene	protein_coding	4.34	0.57	tumors/normal
RP11-1079J22.1	*SNRPD2*	pseudogene	protein_coding	-6.56	0.15	HPV16+/HPV16-
RP11-71B7.1	*SOWAHC*	pseudogene	protein_coding	1.44	-1.06	HPV16+/HPV16-
RP11-166O4.1	*SPDYE1*	pseudogene	protein_coding	1.13	0.83	HPV16+/HPV16-
CH17-52D20.3	*SPDYE3*	pseudogene	protein_coding	1.36	1.03	HPV16+/HPV16-
WTAPP1	*SSBP2*	pseudogene	protein_coding	-2.13	1.35	HPV16+/HPV16-
SUGT1P	*SUGT1*	pseudogene	protein_coding	-1.28	-0.83	HPV16+/HPV16-
SVILP1	*SVIL*	pseudogene	protein_coding	-1.80	-1.14	tumors/normal
RP11-1094M14.8	*TAF5L*	pseudogene	protein_coding	1.64	-0.02	HPV16+/HPV16-
MYO15B	*TBC1D10B*	pseudogene	protein_coding	1.13	0.10	HPV16+/HPV16-
TCEB2P2	*TCEB2*	pseudogene	protein_coding	-3.53	-0.11	HPV16+/HPV16-
RP4-631H13.6	*TMA7*	pseudogene	protein_coding	2.47	0.53	HPV16+/HPV16-
RP11-134G8.2	*TNNI1*	pseudogene	protein_coding	-2.20	-1.97	HPV16+/HPV16-
RP11-480I12.5	*TUBA4A*	pseudogene	protein_coding	1.37	-1.53	HPV16+/HPV16-
RP11-480I12.5	*TUBG1*	pseudogene	protein_coding	1.37	0.15	HPV16+/HPV16-
UBA52P8	*UBA52*	pseudogene	protein_coding	-1.43	0.18	HPV16+/HPV16-
UBA52P6	*UBA52*	pseudogene	protein_coding	3.16	0.18	HPV16+/HPV16-
RP11-726G1.1	*UBXN8*	pseudogene	protein_coding	1.23	0.10	HPV16+/HPV16-
UNGP3	*UNG*	pseudogene	protein_coding	-1.73	0.84	HPV16+/HPV16-
UNGP1	*UNG*	pseudogene	protein_coding	1.13	0.84	HPV16+/HPV16-
RP11-169K16.7	*UQCRH*	pseudogene	protein_coding	1.73	0.57	HPV16+/HPV16-
KRT42P	*USP6*	pseudogene	protein_coding	3.87	-0.15	HPV16+/HPV16-

SLC25A5P1	*WBP2NL*	pseudogene	protein_coding	1.18	0.70	HPV16+/HPV16-
WTAPP1	*WTAP*	pseudogene	protein_coding	-2.13	0.09	HPV16+/HPV16-
YWHAEP7	*YWHAE*	pseudogene	protein_coding	2.25	0.03	HPV16+/HPV16-
ZNF204P	*ZNF461*	pseudogene	protein_coding	-1.25	-0.23	tumors/normal
CTD-3018O17.5	*ZNF766*	pseudogene	protein_coding	-3.74	0.59	tumors/normal
