# Supplementary material for: Non-coding RNAs profiling in head and neck cancers
Source: NPJ Genom Med. 2016 Jan 13;1:15004–. doi: 10.1038/npjgenmed.2015.4 (PMC5685291; doi:10.1038/npjgenmed.2015.4)
Supplement: Supplemental Table 9 [file npjgenmed20154-s9.pdf]

Supplemental table 9: Target's biotype distribution of differentially expressed long non-coding RNA.

"Target" genes biotype	asRNA targets	Other lncRNA targets
Protein coding	548	709
antisense	-	90
lincRNA	30	227
Sense intronic	23	19
Sense overlapping	1	3
Processed transcript	8	14
Processed pseudogene	19	32
Unprocessed pseudogene	2	8
Transcribed processed pseudogene	4	8
Transcribed unprocessed pseudogene	2	17
snoRNA	6	2
Misc RNA	12	9
miRNA	5	21
unknown	91	21
total	751	1180
