# Supplementary material for: Non-coding RNAs profiling in head and neck cancers
Source: NPJ Genom Med. 2016 Jan 13;1:15004–. doi: 10.1038/npjgenmed.2015.4 (PMC5685291; doi:10.1038/npjgenmed.2015.4)
Supplement: Supplemental Table 10 [file npjgenmed20154-s10.pdf]

Supplemental table 10: Protein coding parent genes with multiple differentially expressed pseudogenes

Comparison in which pseudogene is differentially expressed	Pseudogene (other ncRNA)	Protein coding parent gene	Direction of changes in expression in comparison from A column for genes in B and C columns	Parent gene description	Parent gene function (RefSeq)
tumors/controls	*DDX12P, RP11-22B23.1, (DDX11-AS1)*	*DDX11*	UP,UP,(UP),UP	Homo sapiens DEAD/H (Asp-Glu-Ala-Asp/His) box helicase 11	Alteration of RNA secondary structure such as translation initiation, nuclear and mitochondrial splicing, and ribosome and spliceosome assembly
tumors/controls	*LINC01296, DUXAP10*	*FAM98B*	UP, UP, DOWN	Homo sapiens family with sequence similarity 98, member B	unknown function
tumors/controls	*RPS27P23, RP11-10G12.1,*	*RPS27*	DOWN, DOWN, DOWN	Homo sapiens ribosomal protein S27	Ribosomal component of the 40S subunit
tumors/controls	*BMS1P10, BMS1P8*	*BMS1*	DOWN, DOWN, DOWN	Homo sapiens BMS1 ribosome biogenesis factor	Likely encodes a ribosome assembly protein
tumors/controls	*RP11-583F2.1, TSSC2*	*C2CD3*	UP,UP,UP	Homo sapiens C2 calcium-dependent domain containing 3	A protein that functions as a regulator of centriole elongation
HPV16+/HPV16-	*GUSBP2, RP11-1023L17.1, GUSBP3, GUSBP4*	*GUSB*	UP, UP, UP, UP, UP	Homo sapiens glucuronidase, beta	This gene encodes a hydrolase that degrades glycosaminoglycans, including heparan sulfate, dermatan sulfate, and chondroitin-4,6-sulfate
HPV16+/HPV16-	*RPL12P38, HMGN2P8, HMGN2P17, HMGN2P18, HMGN2P15*	*HMGN2*	UP, UP, UP, UP, UP, UP, UP	Homo sapiens high mobility group nucleosomal binding domain 2	Binds nucleosomal DNA and is associated with transcriptionally active chromatin, may help to maintain an open chromatin configuration around transcribable genes, has also been found to have antimicrobial activity against bacteria, viruses and fungi.
HPV16+/HPV16-	*RP11-416L21.2, IFITM9P*	*IFITM3*	DOWN, DOWN, DOWN	Homo sapiens interferon induced transmembrane protein 3	An interferon-induced membrane protein that helps confer immunity to influenza A H1N1 virus, West Nile virus, and dengue virus
HPV16+/HPV16-	*TMPRSS11BNL, FTLP10*	*IFNLR1*	UP, UP, DOWN	Homo sapiens interferon, lambda receptor 1	The protein encoded by this gene belongs to the class II cytokine receptor family. This protein forms a receptor complex with interleukin 10 receptor, beta (IL10RB). The receptor complex has been shown to interact with three closely related cytokines, including interleukin 28A (IL28A), interleukin 28B (IL28B), and interleukin 29 (IL29). The expression of all three cytokines can be induced by viral infection. The cells overexpressing this protein have been found to have enhanced responses to IL28A and IL29, but decreased response to IL28B
HPV16+/HPV16-	*PPIAP26, AC005517.3*	*PPIA*	DOWN, UP, DOWN	Homo sapiens peptidylprolyl isomerase A (cyclophilin A)	This gene encodes a member of the peptidyl-prolyl cis-trans isomerase (PPIase) family. PPIases catalyze the cis-trans isomerization of proline imidic peptide bonds in oligopeptides and accelerate the folding of proteins. The encoded protein is a cyclosporin binding-protein and may play a role in cyclosporin A-mediated immunosuppression. The protein can also interact with several HIV proteins, including p55 gag, Vpr, and capsid protein, and has been shown to be necessary for the formation of infectious HIV virions.
HPV16+/HPV16-	*RP11-677M14.5, RP11-251G23.2*	*RNF181*	UP, UP, UP	Homo sapiens ring finger protein 181	Binds the integrin alpha-Iib/beta-3 complex and has E3 ubiquitin ligase activity
HPV16+/HPV16-	*IGHV3OR16-7, IGHV3OR16-15, RP11-1166P10.8, IGHV3-71*	*RP11-812E19.9*	UP, UP, UP, UP, UP	Putative protein coding gene	unknown function
HPV16+/HPV16-	*UBA52P6, UBA52P8*	*UBA52*	UP, DOWN, UP	Homo sapiens ubiquitin A-52 residue ribosomal protein fusion product 1	Ubiquitin is a highly conserved nuclear and cytoplasmic protein that has a major role in targeting cellular proteins for degradation by the 26S proteosome. It is also involved in the maintenance of chromatin structure, the regulation of gene expression, and the stress response. Ubiquitin is synthesized as a precursor protein consisting of either polyubiquitin chains or a single ubiquitin moiety fused to an unrelated protein. This gene encodes a fusion protein consisting of ubiquitin at the N terminus and ribosomal protein L40 at the C terminus, a C-terminal extension protein (CEP). 60S ribosomal protein L40: component of the 60S subunit of the ribosome. Ribosomal protein L40 is essential for translation of a subset of cellular transcripts, and especially for cap-dependent translation of vesicular stomatitis virus mRNAs.
HPV16+/HPV16-	*UNGP1, UNGP3*	*UNG*	UP, DOWN, UP	Homo sapiens uracil-DNA glycosylase (UNG)	DNA repair. Excises uracil residues from the DNA which can arise as a result of misincorporation of dUMP residues by DNA polymerase or due to deamination of cytosine. Hydrolyzes single-stranded DNA or mismatched double-stranded DNA and polynucleotides, releasing free uracil.
