# Supplementary material for: Non-coding RNAs profiling in head and neck cancers
Source: NPJ Genom Med. 2016 Jan 13;1:15004–. doi: 10.1038/npjgenmed.2015.4 (PMC5685291; doi:10.1038/npjgenmed.2015.4)
Supplement: Supplemental Table 11 [file npjgenmed20154-s11.pdf]

Supplemental table 11: Metacore network analysis using parent genes of differentially expressed pseudogenes in HPV16+ tumors relative to HPV- tumors

#	Network	GO processes	Total nodes	Seed nodes	Pathways	p-Value	zScore	gScore
1	*Elk-1, FOXO3A, CDC25C, Bcl-XL, Bax*	cell cycle (46.0%; 8.424e-38), cell cycle process (41.3%; 1.187e-36), cellular response to DNA damage stimulus (31.3%; 6.035e-30), cell division (28.7%; 1.604e-26), organelle fission (26.0%; 2.161e-26)	156	80	0	7.140E-215	200.9	200.9
2	*Cyclin D1, VEGF-A, HIF1A, DLL4, Jagged2*	Notch receptor processing (11.4%; 4.379e-09), Notch signaling pathway (18.2%; 4.588e-09), tube development (25.0%; 7.533e-07), Notch signaling involved in heart development (6.8%; 1.400e-06), morphogenesis of an epithelium (20.5%; 1.441e-06)	49	35	0	1.290E-96	156.83	156.83
3	*G3P2, SIAH1, Norepinephrine extracellular region, Insulin processed, IGF-1*	fibroblast growth factor receptor signaling pathway (23.3%; 4.653e-16), cellular response to fibroblast growth factor stimulus (23.3%; 3.603e-15), epidermal growth factor receptor signaling pathway (23.3%; 4.557e-15), response to hormone (43.3%; 5.036e-15), response to fibroblast growth factor (23.3%; 5.116e-15)	69	37	0	3.620E-96	142.81	142.81
4	*COX Vb, BMP7, MKL1, MKL2(MRTF-B), AMPK alpha subunit*	positive regulation of macromolecule metabolic process (55.6%; 2.038e-12), viral life cycle (20.4%; 7.222e-12), positive regulation of gene expression (46.3%; 8.384e-12), positive regulation of metabolic process (59.3%; 5.106e-11), nucleic acid metabolic process (63.0%; 6.490e-11)	58	30	0	8.000E-77	125.69	125.69
5	*Troponin I, slow skeletal, MEF2C, AMPK alpha subunit, MKL2(MRTF-B), TNF-alpha*	positive regulation of muscle tissue development (17.5%; 3.359e-11), regulation of muscle tissue development (22.5%; 4.231e-11), nucleic acid metabolic process (70.0%; 7.528e-11), muscle cell differentiation (27.5%; 1.359e-10), positive regulation of gene expression (50.0%; 1.788e-10)	44	24	0	2.480E-62	116.11	116.11
6	*FDPS, Cholesterol cytoplasm, Insulin processed, TNF-alpha, SREBP1 (nuclear)*	regulation of steroid biosynthetic process (25.0%; 3.411e-08), regulation of steroid metabolic process (25.0%; 1.754e-07), regulation of alcohol biosynthetic process (20.0%; 1.048e-06), regulation of lipid biosynthetic process (25.0%; 1.278e-06), positive regulation of steroid biosynthetic process (15.0%; 2.384e-06)	29	17	0	2.560E-45	102.59	102.59
7	*CD8, CD80, CD86, IL-10, IL-10 receptor*	T cell costimulation (66.7%; 6.474e-13), lymphocyte costimulation (66.7%; 6.900e-13), regulation of T cell activation (77.8%; 2.260e-11), regulation of immune effector process (77.8%; 5.690e-11), positive regulation of interleukin-2 biosynthetic process (44.4%; 6.014e-11)	9	1	0	9.100E-03	10.37	10.37
8	*CD8 beta, Immunoproteasome (11S regulator), 26S proteasome (19S regulator), Antigen extracellular region, 26S proteasome (20S core)*	antigen processing and presentation of exogenous peptide antigen via MHC class I, TAP-dependent (60.0%; 4.625e-12), antigen processing and presentation of exogenous peptide antigen via MHC class I (60.0%; 6.658e-12), antigen processing and presentation of peptide antigen via MHC class I (60.0%; 4.561e-11), regulation of cellular amino acid metabolic process (50.0%; 9.524e-11), negative regulation of ubiquitin-protein ligase activity involved in mitotic cell cycle (50.0%; 1.444e-10)	11	1	0	1.110E-02	9.36	9.36
