# Supplementary material for: Non-coding RNAs profiling in head and neck cancers
Source: NPJ Genom Med. 2016 Jan 13;1:15004–. doi: 10.1038/npjgenmed.2015.4 (PMC5685291; doi:10.1038/npjgenmed.2015.4)
Supplement: Supplemental Table 12 [file npjgenmed20154-s12.pdf]

Supplemental table 12: Enrichment by GO processes for the parent genes of the differentially expressed pseudogenes

#	Processes	Total	p-value	FDR	In Data	Network Objects
1	viral process	882	5.251E-13	6.463E-10	23	*Nucleophosmin, UBC, RPLP1, RPS24, RP40, CD8 beta, Cyclophilin A, RPL17, RPL37, CD8, NPM/ALK fusion protein, RPS27A, Ubiquitin, UNG1, RPB7.0, 14-3-3 epsilon, TFIIA gamma chain, FDPS, 14-3-3, MMP-1, RPL23, eIF4H, Elongin B*
2	multi-organism cellular process	888	6.040E-13	6.463E-10	23	*Nucleophosmin, UBC, RPLP1, RPS24, RP40, CD8 beta, Cyclophilin A, RPL17, RPL37, CD8, NPM/ALK fusion protein, RPS27A, Ubiquitin, UNG1, RPB7.0, 14-3-3 epsilon, TFIIA gamma chain, FDPS, 14-3-3, MMP-1, RPL23, eIF4H, Elongin B*
3	symbiosis, encompassing mutualism through parasitism	995	6.199E-12	3.317E-09	23	*Nucleophosmin, UBC, RPLP1, RPS24, RP40, CD8 beta, Cyclophilin A, RPL17, RPL37, CD8, NPM/ALK fusion protein, RPS27A, Ubiquitin, UNG1, RPB7.0, 14-3-3 epsilon, TFIIA gamma chain, FDPS, 14-3-3, MMP-1, RPL23, eIF4H, Elongin B*
4	interspecies interaction between organisms	995	6.199E-12	3.317E-09	23	*Nucleophosmin, UBC, RPLP1, RPS24, RP40, CD8 beta, Cyclophilin A, RPL17, RPL37, CD8, NPM/ALK fusion protein, RPS27A, Ubiquitin, UNG1, RPB7.0, 14-3-3 epsilon, TFIIA gamma chain, FDPS, 14-3-3, MMP-1, RPL23, eIF4H, Elongin B*
5	G2/M transition of mitotic cell cycle	197	4.430E-10	1.580E-07	11	*Tubulin gamma 1, UBC, TSGA14, RP40, Tubulin alpha, RPS27A, Ubiquitin, Tubulin alpha-4A, 14-3-3 epsilon, Tubulin gamma, 14-3-3*
6	cell cycle G2/M phase transition	197	4.430E-10	1.580E-07	11	*Tubulin gamma 1, UBC, TSGA14, RP40, Tubulin alpha, RPS27A, Ubiquitin, Tubulin alpha-4A, 14-3-3 epsilon, Tubulin gamma, 14-3-3*
7	viral gene expression	135	3.936E-09	1.203E-06	9	*UBC, RPLP1, RPS24, RP40, RPL17, RPL37, RPS27A, Ubiquitin, RPL23*
8	multi-organism metabolic process	145	7.387E-09	1.976E-06	9	*UBC, RPLP1, RPS24, RP40, RPL17, RPL37, RPS27A, Ubiquitin, RPL23*
9	translational termination	186	6.426E-08	1.244E-05	9	*MRPL23, RPLP1, RPS24, MRPL39, RP40, RPL17, RPL37, RPS27A, RPL23*
10	mRNA metabolic process	769	6.883E-08	1.244E-05	16	*NUDT16, UBC, RPLP1, RPS24, RP40, RPL17, RPL37, SNRPD2 (SMD2), RPS27A, Ubiquitin, Mago nashi, RPB7.0, 14-3-3, FLJ10292, RPL23, WTAP*

11	translational initiation	251	7.170E-08	1.244E-05	10	*MRPL23, RPLP1, RPS24, MRPL39, RP40, RPL17, RPL37, RPS27A, RPL23, eIF4H*
12	viral life cycle	251	7.170E-08	1.244E-05	10	*UBC, RPLP1, RPS24, RP40, Cyclophilin A, RPL17, RPL37, RPS27A, Ubiquitin, RPL23*
13	nuclear-transcribed mRNA catabolic process, nonsense-mediated decay	135	7.555E-08	1.244E-05	8	*RPLP1, RPS24, RP40, RPL17, RPL37, RPS27A, Mago nashi, RPL23*
14	nucleotide-binding domain, leucine rich repeat containing receptor signaling pathway	59	1.397E-07	2.135E-05	6	*UBC, SUGT1, RP40, Caspase-1, RPS27A, Ubiquitin*
15	multi-organism process	3406	1.541E-07	2.199E-05	34	*G3P2, Nucleophosmin, UBC, RPLP1, HERC2, BGLR, RPS24, FKBP6, RP40, CD8 beta, Caspase-1, Cyclophilin A, RPL17, RPL37, CD8, NPM/ALK fusion protein, WBP2NL, RPS27A, IL28RA, Ubiquitin, UNG1, Mago nashi, RPB7.0, 14-3-3 epsilon, REP8, TFIIA gamma chain, ADHX, IFITM3, FDPS, 14-3-3, MMP-1, RPL23, eIF4H, Elongin B*
16	organic substance catabolic process	2473	2.437E-07	3.079E-05	28	*G3P2, USP6, Tubulin gamma 1, MMP-13, NUDT16, UBC, Cytochrome c, RPLP1, HERC2, BGLR, KIF27, RPS24, RP40, Tubulin alpha, RPL17, RPL37, RPS27A, Ubiquitin, UNG1, Mago nashi, Tubulin alpha-4A, Tubulin gamma, Proline oxidase 1, REP8, Fatty acid-binding protein, ADHX, 14-3-3, RPL23*
17	virion assembly	34	2.446E-07	3.079E-05	5	*UBC, RP40, Cyclophilin A, RPS27A, Ubiquitin*
18	cellular protein complex disassembly	228	3.629E-07	4.315E-05	9	*MRPL23, RPLP1, RPS24, MRPL39, RP40, RPL17, RPL37, RPS27A, RPL23*
19	mRNA catabolic process	231	4.051E-07	4.563E-05	9	*NUDT16, RPLP1, RPS24, RP40, RPL17, RPL37, RPS27A, Mago nashi, RPL23*
20	viral protein processing	16	4.378E-07	4.684E-05	4	*UBC, RP40, RPS27A, Ubiquitin*
21	SRP-dependent cotranslational protein targeting to membrane	118	5.093E-07	5.190E-05	7	*RPLP1, RPS24, RP40, RPL17, RPL37, RPS27A, RPL23*
22	cotranslational protein targeting to membrane	120	5.710E-07	5.554E-05	7	*RPLP1, RPS24, RP40, RPL17, RPL37, RPS27A, RPL23*
23	viral transcription	123	6.751E-07	6.281E-05	7	*RPLP1, RPS24, RP40, RPL17, RPL37, RPS27A, RPL23*
24	translational elongation	247	7.096E-07	6.327E-05	9	*MRPL23, RPLP1, RPS24, MRPL39, RP40, RPL17, RPL37, RPS27A, RPL23*

25	protein targeting to ER	127	8.383E-07	6.991E-05	7	*RPLP1, RPS24, RP40, RPL17, RPL37, RPS27A, RPL23*
26	catabolic process	2792	8.494E-07	6.991E-05	29	*G3P2, USP6, Tubulin gamma 1, MMP-13, NUDT16, UBC, Cytochrome c, RPLP1, HERC2, BGLR, KIF27, RPS24, RP40, Tubulin alpha, RPL17, RPL37, RPS27A, Ubiquitin, UNG1, Mago nashi, Tubulin alpha-4A, Tubulin gamma, Proline oxidase 1, REP8, Fatty acid-binding protein, ADHX, 14-3-3, MMP-1, RPL23*
27	cellular macromolecule catabolic process	931	8.961E-07	7.102E-05	16	*USP6, NUDT16, UBC, Cytochrome c, RPLP1, HERC2, RPS24, RP40, RPL17, RPL37, RPS27A, Ubiquitin, UNG1, Mago nashi, REP8, RPL23*
28	cellular component organization or biogenesis	6225	9.313E-07	7.118E-05	47	*PAF65B, G3P2, Tubulin gamma 1, MMP-13, MPP10, Nucleophosmin, NUDT16, UBC, MRPL23, Cytochrome c, RPLP1, KIF27, Histone H3, TSGA14, SUGT1, RPS24, ISCA1, MRPL39, Myomegalin, FKBP6, FLJ22875, RP40, Histone H3.3, Tubulin alpha, Cyclophilin A, IFT122, RPL17, RPL37, SNRPD2 (SMD2), NPM/ALK fusion protein, WBP2NL, Espin, RPS27A, Connexin 43, Ubiquitin, Tubulin alpha-4A, Phactr4, 14-3-3 epsilon, Tubulin gamma, TFIIA gamma chain, Fatty acid-binding protein, UQCRH, C14orf111, 14-3-3, MMP-1, RPL23, Elongin B*
29	protein complex subunit organization	1880	1.076E-06	7.504E-05	23	*Tubulin gamma 1, Nucleophosmin, MRPL23, RPLP1, Histone H3, RPS24, MRPL39, Myomegalin, RP40, Histone H3.3, Tubulin alpha, RPL17, RPL37, NPM/ALK fusion protein, Espin, RPS27A, Connexin 43, Tubulin alpha-4A, Tubulin gamma, UQCRH, 14-3-3, RPL23, Elongin B*
30	establishment of protein localization to endoplasmic reticulum	132	1.088E-06	7.504E-05	7	*RPLP1, RPS24, RP40, RPL17, RPL37, RPS27A, RPL23*
31	mitotic cell cycle phase transition	423	1.103E-06	7.504E-05	11	*Tubulin gamma 1, UBC, TSGA14, RP40, Tubulin alpha, RPS27A, Ubiquitin, Tubulin alpha-4A, 14-3-3 epsilon, Tubulin gamma, 14-3-3*
32	protein complex disassembly	261	1.122E-06	7.504E-05	9	*MRPL23, RPLP1, RPS24, MRPL39, RP40, RPL17, RPL37, RPS27A, RPL23*
33	cell cycle phase transition	427	1.208E-06	7.837E-05	11	*Tubulin gamma 1, UBC, TSGA14, RP40, Tubulin alpha, RPS27A, Ubiquitin, Tubulin alpha-4A, 14-3-3 epsilon, Tubulin gamma, 14-3-3*
34	RNA catabolic process	268	1.397E-06	8.791E-05	9	*NUDT16, RPLP1, RPS24, RP40, RPL17, RPL37, RPS27A, Mago nashi, RPL23*
35	macromolecular complex disassembly	271	1.531E-06	9.361E-05	9	*MRPL23, RPLP1, RPS24, MRPL39, RP40, RPL17, RPL37, RPS27A, RPL23*

36	macromolecule catabolic process	1105	1.783E-06	1.060E-04	17	*USP6, NUDT16, UBC, Cytochrome c, RPLP1, HERC2, BGLR, RPS24, RP40, RPL17, RPL37, RPS27A, Ubiquitin, UNG1, Mago nashi, REP8, RPL23*
37	regulation of transcription from RNA polymerase II promoter in response to hypoxia	53	2.375E-06	1.374E-04	5	*UBC, RP40, RPS27A, Ubiquitin, Elongin B*
38	organic cyclic compound catabolic process	1266	2.584E-06	1.455E-04	18	*Tubulin gamma 1, NUDT16, Cytochrome c, RPLP1, KIF27, RPS24, RP40, Tubulin alpha, RPL17, RPL37, RPS27A, UNG1, Mago nashi, Tubulin alpha-4A, Tubulin gamma, Proline oxidase 1, 14-3-3, RPL23*
39	nuclear-transcribed mRNA catabolic process	216	2.696E-06	1.479E-04	8	*RPLP1, RPS24, RP40, RPL17, RPL37, RPS27A, Mago nashi, RPL23*
40	protein localization to endoplasmic reticulum	155	3.183E-06	1.703E-04	7	*RPLP1, RPS24, RP40, RPL17, RPL37, RPS27A, RPL23*
41	cellular component disassembly	576	3.485E-06	1.819E-04	12	*MMP-13, MRPL23, Cytochrome c, RPLP1, RPS24, MRPL39, RP40, RPL17, RPL37, RPS27A, MMP-1, RPL23*
42	cellular component organization	6077	3.618E-06	1.843E-04	45	*PAF65B, G3P2, Tubulin gamma 1, MMP-13, Nucleophosmin, NUDT16, UBC, MRPL23, Cytochrome c, RPLP1, KIF27, Histone H3, TSGA14, SUGT1, RPS24, ISCA1, MRPL39, Myomegalin, FKBP6, FLJ22875, RP40, Histone H3.3, Tubulin alpha, Cyclophilin A, IFT122, RPL17, RPL37, SNRPD2 (SMD2), NPM/ALK fusion protein, WBP2NL, Espin, RPS27A, Connexin 43, Ubiquitin, Tubulin alpha-4A, Phactr4, 14-3-3 epsilon, Tubulin gamma, TFIIA gamma chain, Fatty acid-binding protein, UQCRH, 14-3-3, MMP-1, RPL23, Elongin B*
43	heterocycle catabolic process	1203	5.576E-06	2.775E-04	17	*Tubulin gamma 1, NUDT16, Cytochrome c, RPLP1, KIF27, RPS24, RP40, Tubulin alpha, RPL17, RPL37, RPS27A, UNG1, Mago nashi, Tubulin alpha-4A, Tubulin gamma, Proline oxidase 1, RPL23*
44	cellular catabolic process	2402	6.143E-06	2.988E-04	25	*USP6, Tubulin gamma 1, MMP-13, NUDT16, UBC, Cytochrome c, RPLP1, HERC2, KIF27, RPS24, RP40, Tubulin alpha, RPL17, RPL37, RPS27A, Ubiquitin, UNG1, Mago nashi, Tubulin alpha-4A, Tubulin gamma, Proline oxidase 1, REP8, Fatty acid-binding protein, ADHX, RPL23*

45	Notch receptor processing	32	8.232E-06	3.915E-04	4	*UBC, RP40, RPS27A, Ubiquitin*
46	cellular protein metabolic process	3917	1.222E-05	5.621E-04	33	*PAF65B, G3P2, USP6, MMP-13, FAM86A, UBC, MRPL23, FKBP10, RPLP1, HERC2, CaMKK, TSGA14, CaMKK1, RPS24, MRPL39, FKBP6, RP40, Tubulin alpha, RNF181, Cyclophilin A, RPL17, RPL37, NPM/ALK fusion protein, RPS27A, Ubiquitin, Tubulin alpha-4A, REP8, ADHX, GCNT3, MMP-1, RPL23, eIF4H, Elongin B*
47	nucleobase-containing compound catabolic process	1143	1.234E-05	5.621E-04	16	*Tubulin gamma 1, NUDT16, Cytochrome c, RPLP1, KIF27, RPS24, RP40, Tubulin alpha, RPL17, RPL37, RPS27A, UNG1, Mago nashi, Tubulin alpha-4A, Tubulin gamma, RPL23*
48	macromolecular complex subunit organization	2687	1.411E-05	6.289E-04	26	*PAF65B, Tubulin gamma 1, Nucleophosmin, MRPL23, RPLP1, Histone H3, RPS24, MRPL39, Myomegalin, RP40, Histone H3.3, Tubulin alpha, RPL17, RPL37, SNRPD2 (SMD2), NPM/ALK fusion protein, Espin, RPS27A, Connexin 43, Tubulin alpha-4A, Tubulin gamma, TFIIA gamma chain, UQCRH, 14-3-3, RPL23, Elongin B*
49	cellular component biogenesis	2534	1.567E-05	6.652E-04	25	*Tubulin gamma 1, MPP10, Nucleophosmin, KIF27, Histone H3, TSGA14, RPS24, ISCA1, Myomegalin, FLJ22875, Histone H3.3, Tubulin alpha, IFT122, SNRPD2 (SMD2), NPM/ALK fusion protein, WBP2NL, Espin, Connexin 43, Tubulin alpha-4A, Tubulin gamma, TFIIA gamma chain, UQCRH, C14orf111, 14-3-3, Elongin B*
50	negative regulation of chromosome condensation	2	1.616E-05	6.652E-04	2	*Histone H3, Histone H3.3*
