# Supplementary material for: Non-coding RNAs profiling in head and neck cancers
Source: NPJ Genom Med. 2016 Jan 13;1:15004–. doi: 10.1038/npjgenmed.2015.4 (PMC5685291; doi:10.1038/npjgenmed.2015.4)
Supplement: Supplemental Material [file npjgenmed20154-s13.pdf]

Supplemental materials

Human gene expression quantification

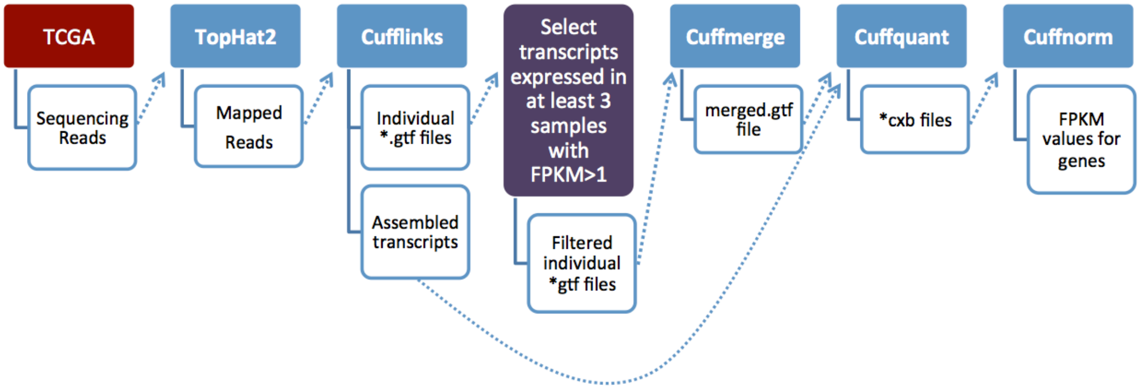

Supplemental figure 1.: **The Cufflinks RNA-seq workflow.**
Blue boxes illustrate conventional data processing steps of Cufflinks suite.

Tophat2 (v2.2.1)[1] and Cufflinks (v2.2.1)[2] were used for human transcripts assembly and quantification. We utilized Cuffmerge[3] to integrate assembled transcripts and Cuffquant[2] to obtain FPKM values. To reduce noise from ultra-low expressed transcripts and avoid memory overflow with Cuffmerge, we only considered transcripts that were expressed in at least three samples with FPKM > 1 and isoform frequency > 0.05.

Below are relevant parameters that were used in Cufflinks RNA-seq workflow:

> tophat2 -p 16 --no-coverage-search -T --transcriptome-index=Homo_sapiens.GRCh38.76 --min-anchor 4 -o output_dir /bowtie2-index/Homo_sapiens.GRCh38.dna.fa input_file_1.fq input_file_2.fq

> cufflinks -p 16 --no-faux-reads --output-dir out_cufflinks --min-isoform-fraction 0.05 --pre-mrna-fraction 0.00 --GTF-guide /ensembl/human/76_nonmasked/Homo_sapiens.GRCh38.76.gtf --mask-file /mtt-rRNA.gtf input_dir/accepted_hits.bam

> cuffmerge -p 16 -o cuffmerge_out_gtf -g /ensembl/human/76_nonmasked/Homo_sapiens.GRCh38.76.gtf -s 76_nonmasked/bowtie2-index/Homo_sapiens.GRCh38.dna.fa.fa cufflinks.gtf.list

> cuffquant -p 16 --mask-file mtt-rRNA.gtf --output-dir output_dir merged.gtf accepted_hits.bam

> cuffnorm --output-dir path_to_sample/cuffnorm -p 16 merged.gtf path_to_sample/abundances.cxb path_to_reference_sample/abundances.cxb

Average time required for RNA-seq data analysis was 4.5 hours per sample. Analysis was parallelized in a high performance cluster environment.
On average, each HNSC sample had 11,757 genes detected with FPKM > 1. Most transcripts were detected in one sample only and may represent false positive assemblies. The average number of genes per sample expressed in at least 3 samples was considerably smaller: N=3,137. On average, 71% of detected isoforms and 60% of genes were novel and not annotated by Ensembl, according to the Cufflinks workflow (Supplemental figure 2). 19,716 genes were expressed with FPKM > 1 in at least 50% of one of the four groups tested ($HPV16^+$, HPV^{low}, HPV^- tumors, and normal tissue). These 19,716 genes were further used for differential expression testing. 17,757 out of 19,716 expressed genes were previously annotated by Ensembl and the remaining genes were assembled by our pipeline *de novo*.

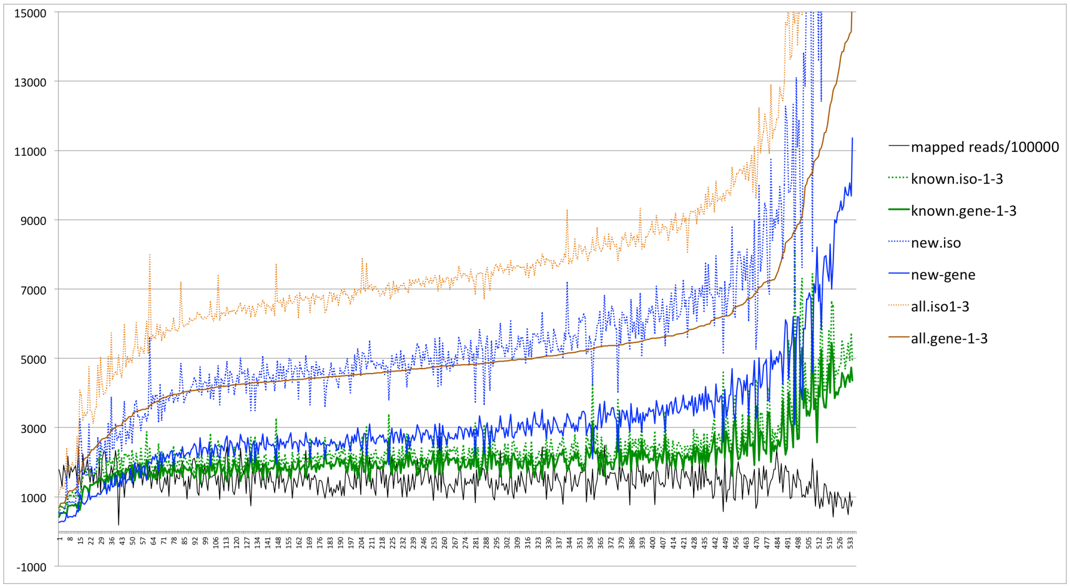

Supplemental figure 2.: **Average number of genes and isoforms per sample**. Only genes expressed in at least 3 samples with FPKM>1 are shown. 71% of isoforms and 60% of genes were novel and not annotated by Ensembl. Legend explanation: "known" – annotated entities, "new" – de-novo assemblies from Cufflinks, "iso" – isoforms, "gene" – genes as defined by Gencode, "1-3" – refers to FPKM>1 and expression in at least in 3 samples. Extremely high or low numbers of novel and known transcripts were not associated with case/control or HPV$^{+/-}$ status (data not shown). Samples with lower total number of reads had higher number of identified transcripts. This bias is probably due to increased false positive detection.

Virus detection and quantification

We have identified two clearly distinct groups of tumors, namely, low and high viral expression (Supplemental figure 3, Supplemental table 1). Low virus expressing group had less than 10^4 nucleotides (<200 reads) aligning to the viral genome while high virus expressing group had greater than 10^5 HPV nucleotides (>2000 reads). Tumors with high HPV16 expression (N=54) were further denoted as HPV16$^+$. Tumor samples with 10^2-10^3 detected HPV nucleotides (2-20 reads) regardless of the type were pooled into HPV^{low} group (N=52) (Supplemental table 1). Tumors without HPV were further referenced as HPV$^-$ (N=289).

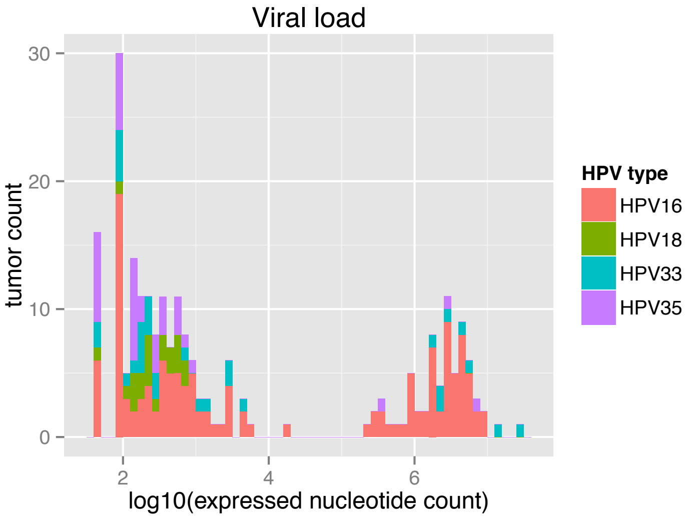

Supplemental figure 3.: **HPV load in 537 HNSC samples from TCGA.**
Viral load (expressed nucleotide count) was calculated by multiplying number of aligned reads by the read length (48)

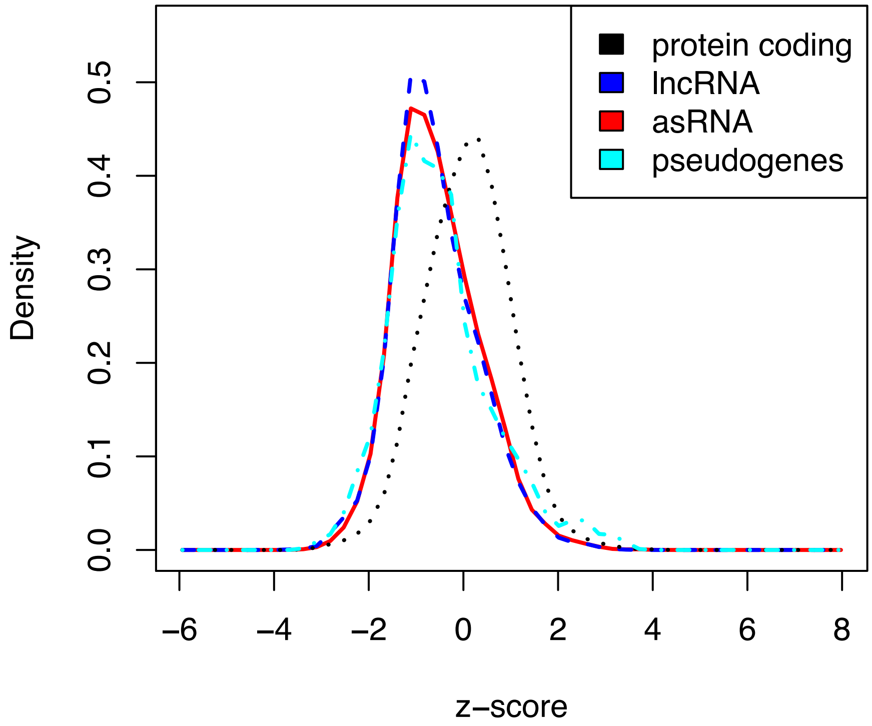

Supplemental Figure 4: **Expression level density plot (z-score) of four most common RNA biotypes.**

Biotypes

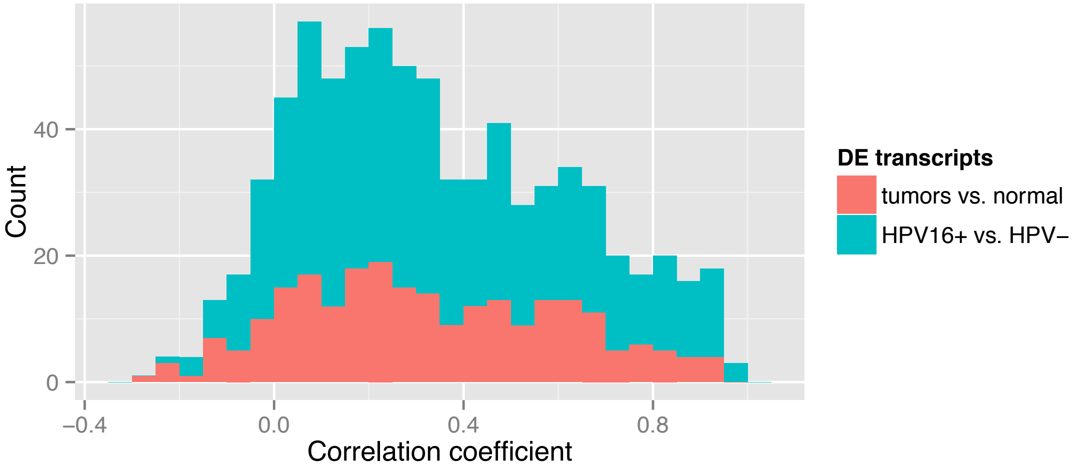

***Supplemental figure 5.:* Distribution of correlation in expression levels between 532 differentially expressed asRNA and their 748 potential targets over all samples.** Here, we separated differentially expressed (DE) antisense genes (asRNA) into two categories: (1) all DE asRNA between tumor and controls independent from the HPV status are called "tumors vs. normal" DE genes (n=179); (2) remaining DE asRNA, specific to the HPV16 status are called "$HPV16^+$ vs. $HPV16^-$" DE genes (n=353).

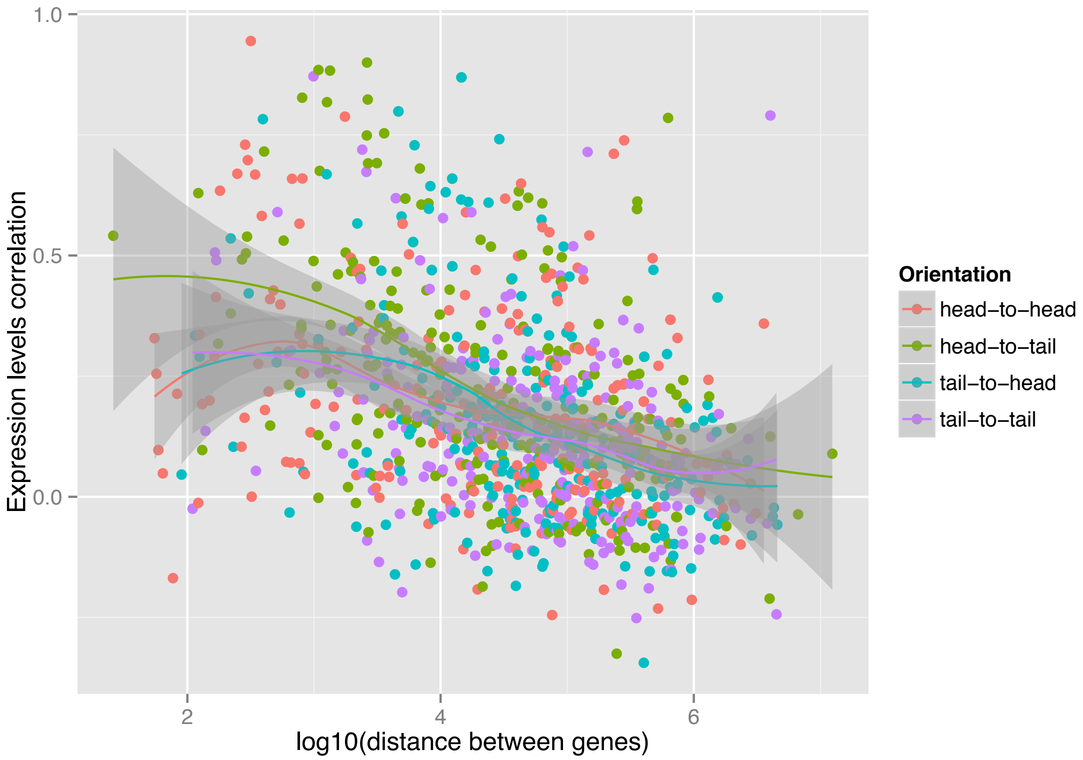

***Supplemental figure 6*.: Co-expression of lncRNA and it's nearest neighbors.**
Each dot represents Pearson correlation between expression levels in a pair of non-overlapping lncRNA (N=590) and it's "target" gene. All studied HNSC tumors and control samples were used to calculate the correlation. Pairs of genes are stratified by transcription orientation in four groups: "head-to-head", "head-to-tail", "tail-to-head" and "tail-to-tail". Lines correspond to the Loess fitted curves for each orientation type. In the distance range 25-10,000 nucleotides, the correlation in the "head-to-tail" pairs is higher than in other orientation types.

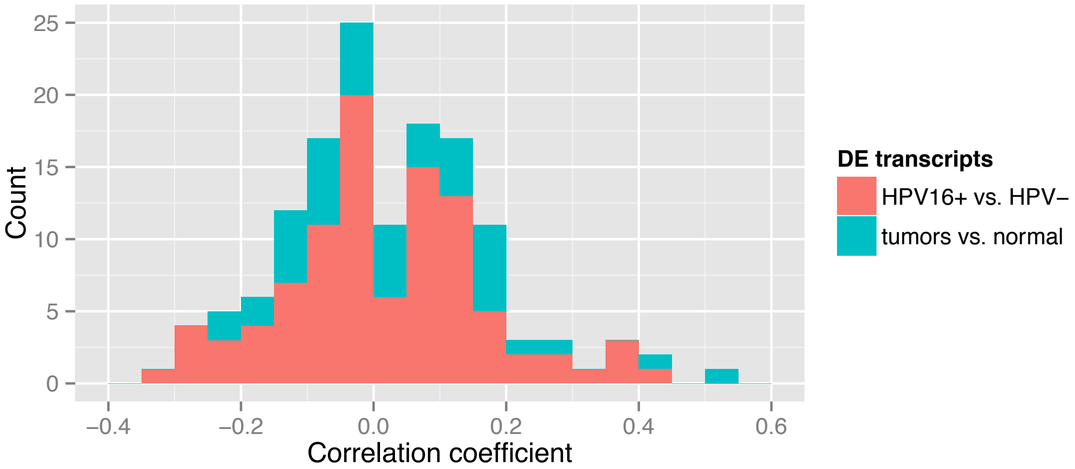

***Supplemental figure 7.:* Correlation between pseudogenes and parent genes expression**

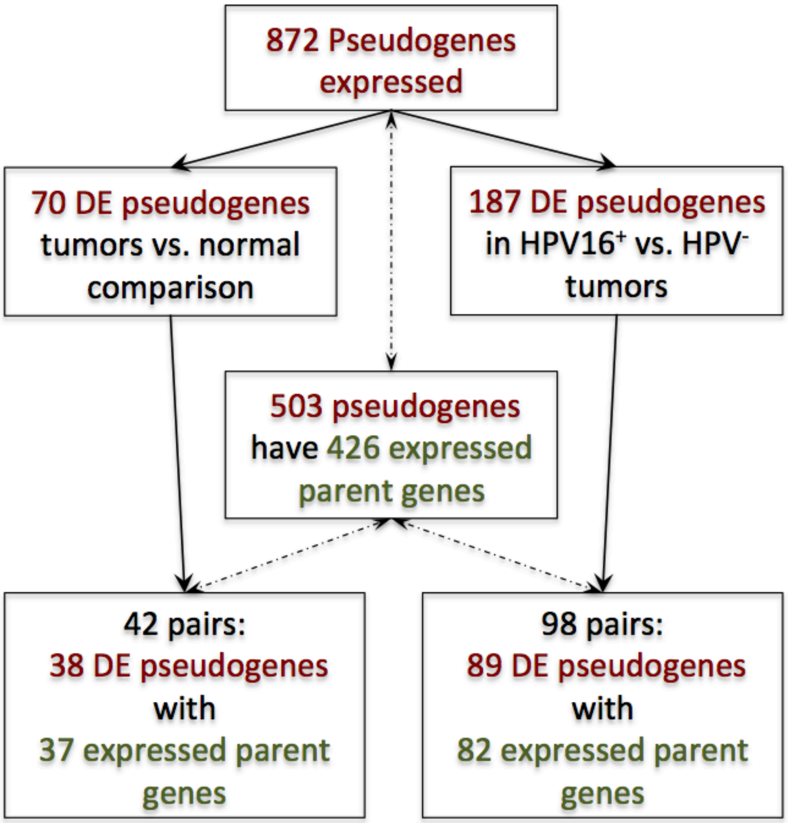

Supplemental figure 8.: **Expressed pseudogenes with protein-coding "parents".** "DE" – differentially expressed genes. Solid arrows identify selecting of DE pseudogene subsets. Dotted arrows reflect BLAST search for protein coding parent genes. Pseudogene-parent pairs were reconstructed at transcript level. Only one best mRNA hit found by BLAST was accepted as parent gene for pseudogenes of interest. Not all pseudogenes had identifiable parents. We also identified multiple pseudogenes for 15 parent genes.

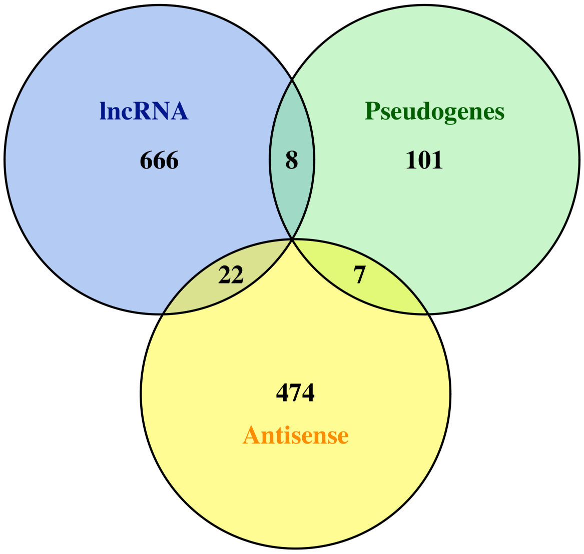

***Supplemental figure 9*.: Number of unique mRNA genes assigned as targets for three largest differentially expressed types of ncRNA.**

References

1 Kim, D. *et al.* TopHat2: accurate alignment of transcriptomes in the presence of insertions, deletions and gene fusions. *Genome Biol* **14**, R36, doi:10.1186/gb-2013-14-4-r36 (2013).

2 Pollier, J., Rombauts, S. & Goossens, A. Analysis of RNA-Seq data with TopHat and Cufflinks for genome-wide expression analysis of jasmonate-treated plants and plant cultures. *Methods Mol Biol* **1011**, 305-315, doi:10.1007/978-1-62703-414-2_24 (2013).

3 Trapnell, C. *et al.* Differential gene and transcript expression analysis of RNA-seq experiments with TopHat and Cufflinks. *Nat Protoc* **7**, 562-578, doi:10.1038/nprot.2012.016 (2012).
